# Polyhydric Stigmastane-Type Steroids Derivative from *Vernonia amygdalina* and Their Anti-Neuroinflammatory Activity

**DOI:** 10.3390/ph15091160

**Published:** 2022-09-19

**Authors:** Xiangzhong Liu, Mi Zhou, Shoulun He, Qiannan Xu, Chunchun Du, Honghong Zhu, Ting Lin, Guanghui Wang, Wenjing Tian, Haifeng Chen

**Affiliations:** Fujian Provincial Key Laboratory of Innovative Drug Target, School of Pharmaceutical Sciences, Xiamen University, Xiamen 361005, China

**Keywords:** *Vernonia amygdalina* Del., steroid, structures elucidation, anti-neuroinflammatory activity, BV-2 microglia cells

## Abstract

*Vernonia amygdalina* Del. is a traditional medicinal plant and vegetable originating from tropical Africa. The phytochemical investigation of *V. amygdalina* led to eight undescribed polyhydric stigmastane-type steroids, vernonin M–T (**1**–**8**). Their gross structures and stereochemistry were elucidated by HR-ESI-MS, 1D and 2D NMR spectra, X-ray diffraction, quantum chemical computation of the ECD spectrum, and the in situ dimolybdenum CD method. The anti-neuroinflammatory activity of the isolated compounds was performed in BV-2 microglia cells. As a result, compound **1** displayed a notable anti-neuroinflammatory effect via suppressing the LPS-induced IκB degradation and restricting the activation of the PI3K/AKT and p38 MAPK pathways.

## 1. Introduction

Natural products are great treasures for the discovery of lead compounds in drug innovation [1]. *Vernonia amygdalina* Delile, belonging to the Compositae family, is a shrub originating from tropical Africa and is widely distributed throughout the southeast coastal area of China [2]. *V. amygdalina* contains abundant bioactive constituents, such as sesquiterpene lactone, steroids, flavone, and so on [3]. The extract of *V. amygdalina* is reported to possess anticancer [4,5,6], antioxidant [7,8], antimalarial [9], antidiabetic [10], anthelmintic [11] and antibacterial [12] bioactivities. In addition, the leaves of *V. amygdalina* are used as a conventional vegetable in Nigeria [13]. *V. amygdalina* can also be used as insect antifeedants and applied in the agricultural industry [14,15]. Recent research has uncovered some undescribed steroid saponins from *V. amygdalina*, and their cytotoxicity [16,17], anti-inflammatory [18], and a-amylase and a-glucosidase inhibitory activities [19] were reported. The diverse bioactivities and chemical components of *V. amygdalina* intrigue our interest to further mine and probe more bioactivity constituents.

Neuroinflammation plays a pivotal role in the pathogenesis of neurodegenerative diseases [20]. Microglia cells play an important role in regulating neuronal function [21]. Excessive activation of microglia cells leads to the release of a succession of inflammatory cytokines [22,23]. There have been reports that activating the NF-κB (nuclear factor-κB), PI3K/AKT (the phosphatidylinositol 3 kinase/AKT), or MAPK (mitogen-activated protein kinase) signaling pathways in microglia cells could accelerate the pathogenetic process of neuroinflammation [24,25,26,27]. Thus, it is a practicable strategy to find small molecules that inhibit the NF-κB, PI3K/AKT, and MAPK signaling pathways to restrict the pathogenetic process of neuroinflammation for the prevention and treatment of neurodegenerative diseases.

**Figure 1:**
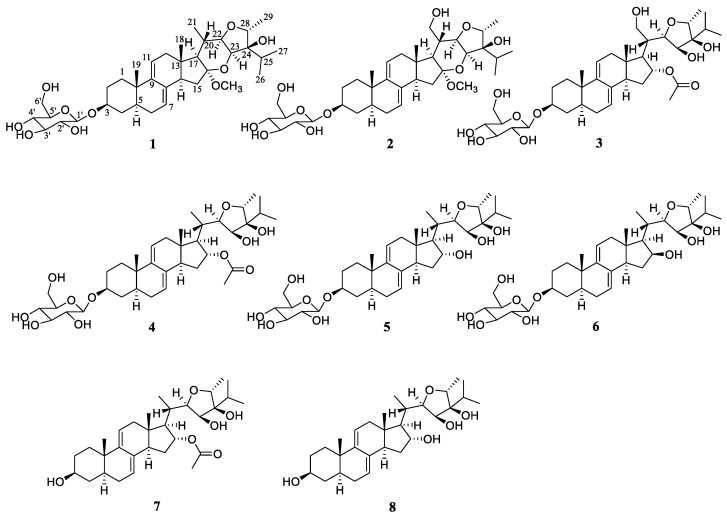
Structures of **1**–**8** isolated from *V. amygdalina*.

In this study, continuous phytochemical and bioactivity screenings of *V. amygdalina* led to eight undescribed polyhydric stigmastane-type steroids (Figure 1). All the isolated compounds were evaluated for their anti-neuroinflammatory activity in BV-2 microglia cells. Among them, compound **1** exhibited obviously anti-neuroinflammatory effects by blocking the degradation of IκB as well as inhibiting the PI3K/AKT and p38 MAPK pathways. Herein, the isolation, structural elucidation, and biological evaluations of **1**–**8** were reported.

## 2. Results and Discussion

Compound **1** was obtained as needle crystals. Its molecular formula was assigned as C_36_H_56_O_10_ by the HR-ESI-MS data with nine indices of hydrogen deficiency. The ^1^H NMR spectrum of **1** (Table 1) indicated the presence of two olefinic protons (*δ*_H_ 5.42 (1H, br s) and 5.45 (1H, br d, *J* = 5.8 Hz)), four methyl doublets (*δ*_H_ 1.24 (3H, d, *J* = 6.7 Hz), 1.21 (3H, d, *J* = 6.7 Hz), 1.15 (3H, d, *J* = 6.3 Hz), and 1.07 (3H, d, *J* = 6.5 Hz), two methyl singlets (*δ*_H_ 0.60 (3H, s) and 0.81 (3H, s)), and one methoxy (*δ*_H_ 3.35 (3H, s)). The ^13^C NMR data (Table 1) combined with DEPT-135 and HSQC spectra revealed 36 carbon resonances, including seven methyl groups, seven methylene carbon signals, sixteen methine carbon signals, and six quaternary carbon signals. Furthermore, four olefinic carbons (*δ*_C_ 144.5, 135.2, 121.6, and 119.0) could be observed at low field region. Seven methyl groups (*δ*_C_ 50.5, 19.9, 18.2, 18.2, 17.9, 17.3, and 13.7) could be assigned at the high-field region. The chemical shifts ranged from *δ*_C_ 63.3 to 111.0 were deduced as oxygenated carbon atoms. In addition, a series of characteristic carbon signals at *δ*_C_ 102.8, 79.1, 78.9, 75.8, 72.1, and 63.3 were assigned as sugar. To compare the NMR data of **1** to the reported structures [28], compound **1** could be inferred as a stigmastane-type steroid saponin.

**Figure 2:**
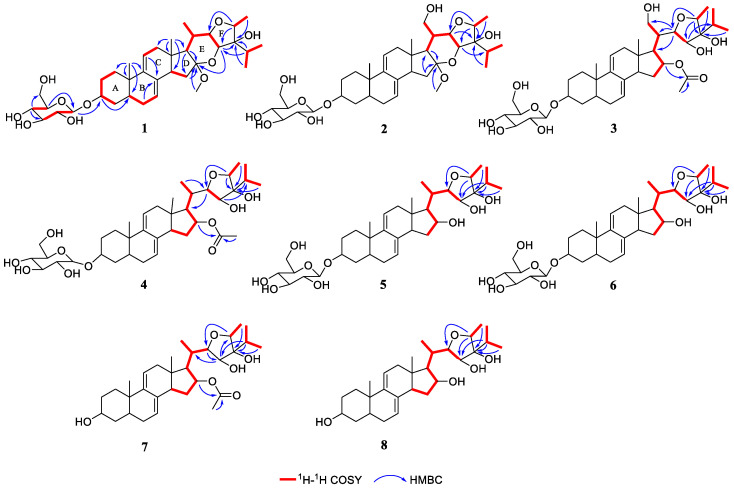
Key ^1^H-^1^H COSY and HMBC correlations of compounds **1**–**8**.

Further structural elucidation of **1** was performed by inspection of its 2D NMR spectra (Figure 2). The ^1^H-^1^H COSY cross peaks of H_2_-1/H_2_-2/H-3/H_2_-4, H-5/H_2_-6/H-7, H-11/H_2_-12, and H-14/H_2_-15 showed the presence of four spin systems, which could be connected by the HMBC correlations from H_3_-19 to C-1, C-5, C-9 and C-10; from H-3 to C-5; from H_2_-6 to C-8; from H-11 to C-8; from H_3_-18 to C-12, C-13, C-14 and C-17; from H_2_-15 to C-16; and from H-17 to C-16 to establish a stigmastane-type steroid core (ring A to D). The ^1^H-^1^H COSY cross peaks of H-1′/H-2′/H-3′ and H-4′/H-5′ and the HMBC correlations from H-4′ to C-3′, C-6′ and from H-5′ to C-1′ combined with the coupling constant of an anomeric proton (*J* = 7.7 Hz) suggested the presence of a *β*-glucose. The sugar moiety was linked to C-3 by the HMBC correlation between H-1′ and C-3. Besides, a methoxy group was linked to C-16 by the HMBC correlation between OCH_3_-16 and C-16. The above structural fragments occupied seven degrees of unsaturation. Therefore, two additional ring systems should exist in the side chain. The ^1^H-^1^H COSY correlations of H-17/H-2/H-20/H_3_-21, H-20/H-22/H-23, H_3_-26/H-25/H_3_-27, and H-28/H_3_-29 together with the HMBC correlations from H-23 to C-16, C-24; from H-25 to C-23, C-24; from H_3_-26 to C-24; from H-28 to C-22, C-23; and from H_3_-29 to C-24 suggested the presence of ring E and ring F.

**Figure 3:**
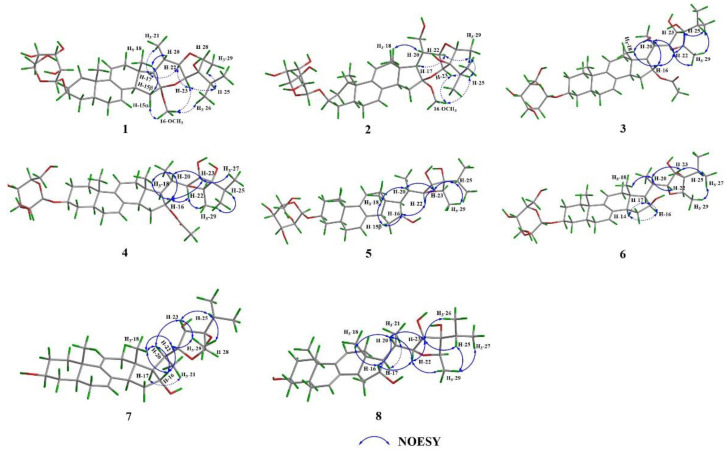
Key NOESY correlations of compounds **1**–**8**.

**Figure 4:**
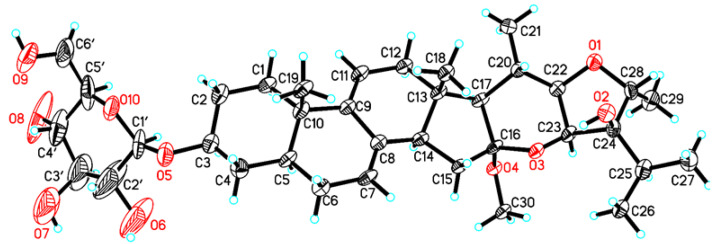
X-ray ORTEP drawing of **1**.

**Figure 5:**
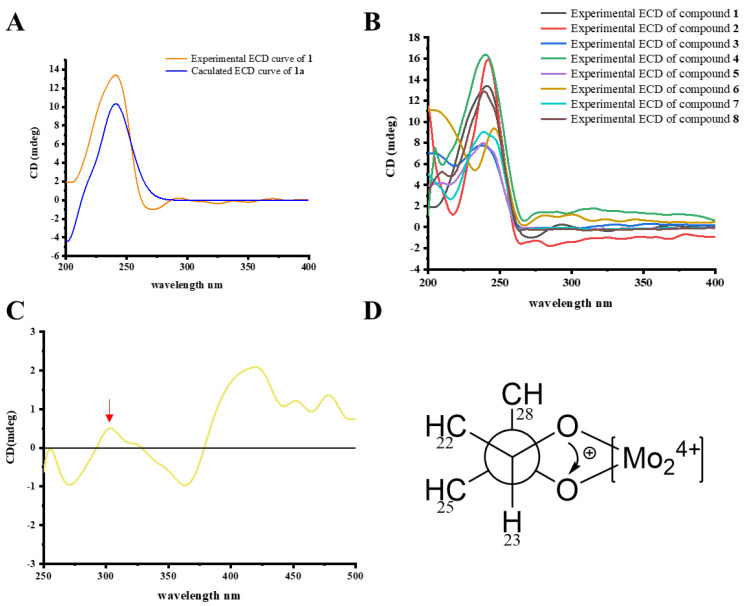
(**A**) Experimental and calculated ECD spectra of **1**. (**B**) Experimental ECD spectra of **1**–**8**. (**C**) ICD spectrum of the Mo_2_^4+^complex of **7** in DMSO. (**D**) Conformations of the Mo_2_^4+^ complex of **3**.

The relative stereochemistry of **1** was assigned based on a NOESY experiment (Figure 3). The NOESY cross peaks of H_3_-18/H-20 indicated the same orientation of H_3_-18 and H-20. The boat conformation of ring E was confirmed by the NOESY correlations of H-15*α*/16-OCH_3_, 16-OCH_3_/H-23, H_3_-21/H-17, H-17/H-22, and H-22/H-23. The NOESY correlations of 16-OCH_3_/H_3_-26 and H_3_-29/H-25, H-25/H-23 suggested that H_3_-29 and isopropyl segment [–CH_3_ (26)–CH (25)–CH_3_ (27)–] were at the same site of ring F. A suitable crystal was obtained from the methanol solution for the X-ray diffraction experiment and the result confirmed the relative configuration of **1** (Figure 4). However, the Flack parameter = 0.40(6) was unable to establish the absolute configuration of **1**. Hence, the quantum chemical computation of electronic circular dichroism (ECD) spectrum was used for determining the absolute configuration of **1**. Based on the similar curve tendency between the experimental ECD spectrum of **1** and the calculated ECD spectrum of (3*S*, 5*S*, 10*S*, 13*S*, 14*R*, 16*S*, 17*R*, 20*S*, 22*R*, 23*S*, 24*R*, 28*R*)-**1a** (Figure 5A), the sugar could be assigned as *β*-D-glucose, and the absolute configuration of **1** was assigned as 3*S*, 5*S*, 10*S*, 13*S*, 14*R*, 16*S*, 17*R*, 20*S*, 22*R*, 23*S*, 24*R*, 28*R*.

The molecular formula of compound **2** was assigned as C_36_H_56_O_11_ from its HR-ESI-MS data. To closely compare the NMR data (Table 1) between **1** and **2**, there was a subtle difference in the gross structure except for the existence of an extra hydroxy group at C-21 of **2**. The ^1^H-^1^H COSY cross peaks of H-17/H-20/H_2_-21 combined with the chemical shift of C-21 (*δ*_C_ 60.2), further confirming the deduction. The similar NOESY correlations and the ECD curves between **1** and **2** (Figure 5B) suggested that they have the same relative and absolute configurations. Thus, the absolute configuration of **2** was deduced as 3*S*, 5*S*, 10*S*, 13*S*, 14*R*, 16*S*, 17*R*, 20*R*, 22*R*, 23*S*, 24*R*, 28*R*.

Compounds **3** and **4** were isolated as white amorphous solids. Their molecular formula were identified as C_37_H_58_O_12_ and C_37_H_58_O_11_ by the positive ion peak at *m/z* 717.3817 [M + Na]^+^ (calculated for C_37_H_58_O_12_Na, 717.3820) and *m/z* 701.3868 [M + Na]^+^ (calculated for C_37_H_58_O_11_Na, 701.3871), respectively. An extensive comparison the NMR data of **2** and **3** suggested that they had the same core structure. The side chain fragment of **3** was elucidated by its 2D NMR spectra. The ^1^H-^1^H COSY cross peaks of H_2_-15/H-16/H-17/H-20/H_2_-21, H-22/H-23, H_3_-26/H-25/H_3_-27, and H-28/H_3_-29 afforded four structural fragments, which were linked by the HMBC correlation from H-22 to C-17, C-20, C-21; from H-23 to C-25; from H_3_-26 to C-24; from H-28 to C-22, C-23, C-24; and from H_3_-29 to C-24. The acetoxy group was connected to the C-16 by the HMBC correlations from H-16 to *δ*_C_ 171.0 and from *δ*_H_ 2.13 to *δ*_C_ 171.0. The NOESY experiment was launched for interpreting the relative configuration of **3**. The NOESY correlations between H_3_-18/H-20, H_3_-18/H-16 and H-16/H-20 suggested these protons possessed the same orientation. The NOESY correlations between H-16/H-22 and H-20/H-22 as well as a small coupling constant (*J* = 6.4 Hz) between H-20 and H-22 inferred they were at the same side. Besides, the NOESY correlations between H-22/H-23, H-23/H-25, H-25/H_3_-29, and H-22/H_3_-29 suggested these protons have the same orientation. To observe the overall structures of **2** and **3**, it was easily conjectured that **3** was derived from **2** via breaking the oxygen bridge between C-16 and C-23. Meanwhile, the same ECD curve tendency (Figure 5B) between **2** and **3** indicated that they had the same stereochemistry. Therefore, the absolute configuration of **2** was assigned as 3*S*, 5*S*, 10*S*, 13*S*, 14*R*, 16*R*, 17*R*, 20*R*, 22*R*, 23*S*, 24*S*, 28*R* based on their biogenetic relationship. The similar ^1^H and ^13^C NMR data (Table 2) of **3** and **4** hinted that they possessed almost the same architecture except for an additional hydroxy group at C-21 in **3**. The absolute configuration of **4** was assigned as 3*S*, 5*S*, 10*S*, 13*S*, 14*R*, 16*R*, 17*R*, 20*S*, 22*R*, 23*S*, 24*S*, 28*R* because the NOESY cross peaks and the ECD curve were both identical to **3**. Their glycosyl at C-3 were determined as *β*-D-glucose due to having the same NMR data as well as the biogenetic relationship to **1**.

Compounds **5** and **6** were obtained as white amorphous solids. The molecular formula of **5** and **6** were deduced as C_35_H_56_O_10_ according to their positive ion peaks at *m/z* 659.3767 [M + Na]+ (calculated for C_35_H_56_O_10_Na, 659.3766) and *m/z* 659.3765 [M + Na]+ (calculated for C_35_H_56_O_10_Na, 659.3766), respectively. The ^1^H and ^13^C NMR data (Table 2) of **5** were similar with **4**, except for a hydroxyl group at C-16 in **5** instead of the acetyl group in **4**. The deduction was further confirmed by the ^1^H-^1^H COSY cross peaks of H_2_-15/H-16/H-17/H-20/H_3_-21, H-20/H-22/H-23, H_3_-26/H-25/H_3_-27, and H-28/H_3_-29, as well as the HMBC correlations from H-25 to C-23, C-2; from H_3_-26 to C-24; from H-28 to C-22, C-23; and from H_3_-29 to C-24. The NMR data of **6** was similar to that of **5** with only an arresting difference at C-16. The 2D NMR spectra of **6** shared a cluster of identical correlation signals to those of **5**, indicating that **5** and **6** had the same planar structure. The different relative configurations of **5** and **6** were interpreted by the NOESY experiment. The NOESY cross peaks of **5** and **6** were similar to each other except for the correlations of H-16/H-15*β*/H_3_-18 in **5** and H-16/H-17/H-14 in **6**, indicating that **5** and **6** were a pair of C-16 epimers. Their saccharide groups were identical to those of **1** based on the same NMR data and biogenetic relationship. The ECD curves of **5** and **6** were identical to those of **4**, which assigned the absolute configuration of **5** as 3*S*, 5*S*, 10*S*, 13*S*, 14*R*, 16*R*, 17*R*, 20*S*, 22*R*, 23*S*, 24*S*, 28*R* and **6** as 3*S*, 5*S*, 10*S*, 13*S*, 14*R*, 16*S*, 17*R*, 20*S*, 22*R*, 23*S*, 24*S*, 28*R*.

Compound **7**, obtained as white powder, gave a molecular formula of C_31_H_48_O_6_ based on its HR-ESI-MS data. The ^1^H and ^13^C NMR data (Table 1) exhibited the same framework as that of **4**, except for the absence of a sugar at C-3 in **7**. The deduction was further confirmed by the difference of their MS data. Therefore, **7** was an aglycone of **4**. The similar NOESY cross peaks between **4** and **7** indicated that they had identical relative configurations. The stereogenic center of *vic*-diols at the hydrogenated furan ring moiety was determined by the induced CD spectrum (ICD) [29]. The characteristic positive cotton effect of Mo_2_^4+^complex of **7** at band IV (303 nm) inferred the absolute configuration of C-23 and C-34 as 23*S*, 24*S* according to the empirical rule. Furthermore, the overall pattern of the ECD spectrum of **7** was identical to that of **4**, indicating that they have the same absolute configuration. Therefore, the absolute configuration of **7** was determined as 3*S*, 5*S*, 10*S*, 13*S*, 14*R*, 16*R*, 17*R*, 20*S*, 22*R*, 23*S*, 24*S*, 28*R*.

Compound **8**, isolated as a white amorphous solid, showed a molecular formula of C_29_H_46_O_5_ deduced by its positive HR-ESI-MS ion peak [M + Na]+ at *m/z* 497.3241 (calculated for C_29_H_46_O_5_Na, 497.3237), implying seven degrees of unsaturation. The ^1^H and ^13^C NMR data (Table 1) of **8** were similar to those of **7** with the same stigmastane-type steroid core architecture. The differences lie in an acetoxyl group at C-3 in **8** instead of a hydroxyl group substituted in **7**, which was confirmed by the ^1^H-^1^H COSY and HMBC spectra. The same NOESY correlations between **7** and **8** suggested that they had the same relative configuration. The absolute configuration of **8** was deduced as 3*S*, 5*S*, 10*S*, 13*S*, 14*R*, 16*R*, 17*R*, 20*S*, 22*R*, 23*S*, 24*S*, 28*R*, since the same cotton effects of ECD curves between **7** and **8** (Figure 5B).

**Figure 6:**
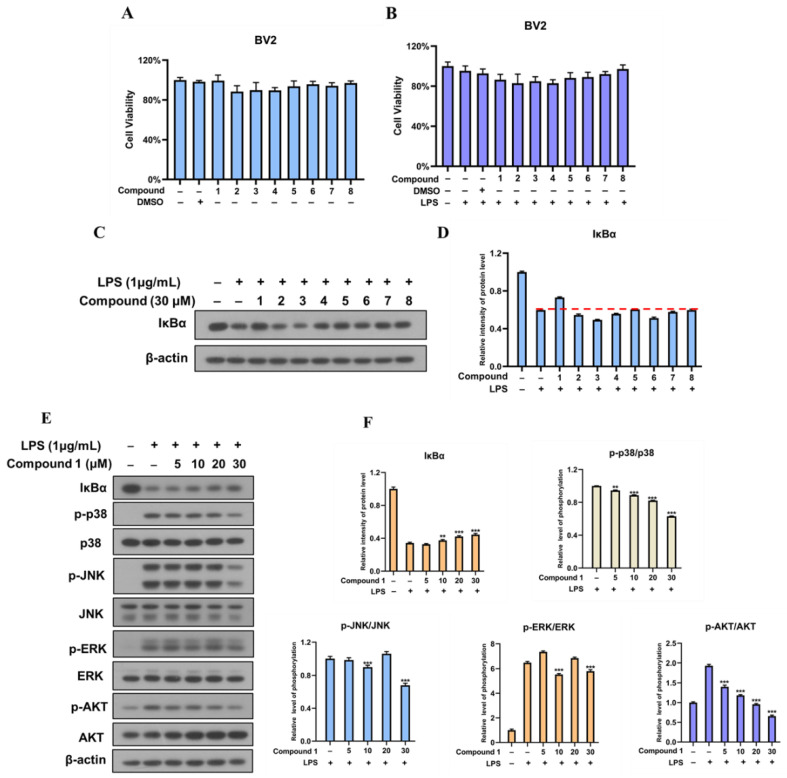
(**A**) BV-2 microglia cells were treated with compounds **1**–**8** (30 μM) or DMSO (0.03%) for 24 h. The viability of cells was measured by MTT assay. (**B**) BV-2 microglia cells were treated with compounds **1**–**8** (30 μM) or DMSO (0.03%) for 24 h. Then, cells were incubated with LPS (1μg/mL) for 30 min. The viability of cells was evaluated by MTT assay. (**C**) BV-2 microglia cells were treated with compounds 1–8 (30 μM) for 24 h. After exposed to LPS (1μg/mL), cells were harvested and the expression of IκBα was analyzed by western blotting. (**D**) Quantitation of the expression as shown in C was analyzed by image J. (**E**) BV-2 cells were treated with compound **1** (5-30 μM) and LPS, and then the expression of proteins was measured via western blotting. (**F**) Quantitation of the expression as shown in E was analyzed by image J. Statistical analysis was performed via GraphPad Prism 8 (** *p* < 0.01, *** *p* < 0.001compared with cells treated with only LPS).

The NF-κB signaling pathway plays a critical role in inflammatory diseases, including neuroinflammatory. The activation of NF-κB depends on the degradation of NF-κB inhibitory proteins (IκBs) [30]. Once activated by bacterial lipopolysaccharide (LPS), IκBs are rapidly phosphorylated and degraded. Then, the IκB degradation promotes NF-κB moving into the nucleus, which contributes to the transcriptional induction of inflammation-associated genes. Thus, blocking the LPS-stimulated degradation of IκBα inhibits NF-κB activation. In this study, western blotting was used to investigate whether the isolates could block the LPS-stimulated degradation of IκBα in BV-2 microglia cells. In order to exclude the cytotoxic effect of compounds on the cell growth of BV2 cells, the cells were exposed to **1**–**8** (30 μM) in the presence or absence of LPS, and cell viability was not affected by the treatment of these compounds (Figure 6A,B). As shown in Figure 6C,D, the IκBα was markedly degraded after treatment with LPS, while the LPS-induced IκBα degradation was blocked by pre-treatment with compound **1** at the concentration of 30 μM. Furthermore, dose–response analysis indicated that compound **1** reversed IκBα degradation in a dose-dependent manner (Figure 6E,F). These results suggest that compound **1** could inhibit the LPS-induced activation of NF-κB signaling pathway in BV-2 microglia cells by the suppression of IκB degradation.

Studies have shown that the PI3K/AKT signaling pathway is able to trigger NF-κB activation through IκB degradation [31]. MAPKs, an important signaling pathway, are involved in the production of pro-inflammatory mediators and cytokines as well as the modulation of NF-κB in microglia BV2 cells [31,32]. Stimulation of BV2 cells with LPS led to the phosphorylation of PI3K and AKT as well as p38 MAPK, extracellular signal-regulated kinase (ERK), and c-Jun N-terminal kinase (JNK). However, compound **1** decreased the phosphorylation of PI3K/AKT and p38 MAPK in a dose-dependent manner (Figure 6E, F). These results confirmed that compound **1** might inhibit inflammation through restricting the activation of the PI3K/AKT and p38 MAPK pathways.

## 3. Materials and Methods

### 3.1. General Experimental Procedures

Ultraviolet (UV) spectra were measured on a Shimadzu UV-2600 spectrophotometer. Optical rotation values were detected on a Rudolph Autopol IV/IV-T automatic polarimeter (Hackettstown, NJ, USA). Electronic circular dichroism (ECD) spectra were obtained on a MOS-500 CD spectrometer (Bio-Logic, Seyssinet-Pariset, France). High-resolution electrospray ionization mass spectra were acquired by a Thermo Scientific Q Exactive Quadrupole-Orbitrap mass spectrometer. NMR spectra were recorded on Bruker AVANCE III 600 MHz or 850 MHz NMR spectrometers (Bruker, Frankfurt, Germany) with TMS as the internal standard at room temperature. Analytical HPLC was carried out on a Shimadzu LC-20AD pump system with a diode-array detector equipped with an RP-C18 HPLC column (250 × 4.6 mm, 5 μm). Semi-preparative HPLC was performed on a Shimadzu LC-16P series instrument with a dual-wavelength UV detector using YMC-Pack ODS-A (250 × 10 mm, 5 μm) chromatographic columns (CC). The silica gel (200–300 mesh, Qingdao Marine Chemical Inc., Qingdao, China), Sephadex LH-20 (Amersham Pharmacia Biotech, Uppsala, Sweden) and ODS (50 μm, YMC, Kyoto, Japan) were employed for column chromatography. Thin-layer chromatography (TLC) (Qingdao Marine Chemical Inc., Qingdao, China) was used for monitoring the chromatography. The petroleum ether-ethyl acetate and dichloromethane-methanol solvent systems were used as mobile phase, and the spots were observed by UV lamp and heated after spraying with 5% H_2_SO_4_ in EtOH (*v*/*v*).

### 3.2. Plant Material

The twigs of *V. amygdalina* (compositae) were harvested from Taiwan, People’s Republic of China, in April 2016, and the species identification was carried out by Prof. Zhenji Li, College of the Environment Ecology, Xiamen University.

### 3.3. Extraction and Isolation

The air-dried and pulverized twigs of *V. amygdalina* were extracted by refluxing with 60% ethanol. The solvents were removed to yield 1 kg crude extract (extraction yield: 18.2%), which was dissolved in distilled water and partitioned with petroleum ether (3 × 4 L), dichloromethane (3 × 4 L), ethyl acetate (3 × 4 L) and n-butyl alcohol (3 × 4 L). The dichloromethane fraction was further separated into eleven fractions (Fr. 1–11) by silica gel CC (150 × 1400 mm) and was eluted with a CHCl_2_–CH_3_OH gradient system from 100:0 to 80:20 (*v*/*v*) as mobile phase. Fr. 8 (16.2 g) was subjected to ODS CC (80 × 500 mm) with CH_3_OH–H_2_O (50:50, *v*/*v*) to afford twelve fractions (Fr. 8.1–8.12). Further, Fr. 8.12 (1.8 g) was purified by semipreparative HPLC (CH_3_OH–H_2_O, 70:30, *v*/*v*) to yield compound **1** (3.7 mg). Compounds **2** (2.9 mg) and **3** (13.0 mg) were isolated from Fr. 8.5 (11.9 g) by silica gel CC (100 × 1000 mm) eluting with CHCl_2_–CH_3_OH (from 100:1 to 5:1, *v*/*v*), and then purified by semipreparative HPLC (ACN–H_2_O, 40:60, *v*/*v*). Fr. 8.8 (576.9 mg) was separated by ODS CC (50 × 250 mm) with CH_3_OH–H_2_O (45:55, *v*/*v*) and subsequently was purified using semipreparative HPLC (ACN–H_2_O, 35:65, *v*/*v*) to obtain compound **6** (157.2 mg). Fr. 8.7 (746.9 mg) was separated by Sephadex LH-20 column chromatography (40 × 2000 mm) with CH_2_Cl_2_/CH_3_OH (1:1, *v*/*v*) as mobile phase to obtained four fractions (Fr.8.7.1–8.7.4). Fr.8.7.2 (195.0 mg) was purified by semipreparative HPLC (CH_3_OH–H_2_O, 60:40, *v*/*v*) to yield compounds **4** (0.7 mg), **5** (2.9 mg), **7** (29.6 mg) and **8** (23.0 mg).

Vernonin M (**1**)

Colorless needle crystals; ${\left[\alpha \right]}_{D}^{24}$ + 56.68 (c 2.47, CH_3_OH); UV (CH_3_OH) *λ*_max_ (log *ε*) = 234 (4.08), 242 (4.13) and 250 (3.95) nm; ECD (CH_3_OH) *λ*_max_ (Δ*ε*) 241 (+4.26) nm; HR-ESI-MS *m/z* 671.3769 [M + Na]^+^ (calculated for C_36_H_56_O_10_Na, *m/z* 671.3766); ^1^H and ^13^C NMR data see Table 1.

Vernonin P (**2**)

White amorphous solid; ${\left[\alpha \right]}_{D}^{29}$ + 65.18 (*c* 0.58, CH_3_OH); UV (CH_3_OH) *λ*_max_ (log *ε*) = 235 (4.12), 242 (4.17) and 250 (3.97) nm; ECD (CH_3_OH) *λ*_max_ (Δ*ε*) 240 (+5.81) nm; HR-ESI-MS *m/z* 663.3727 [M-H]^-^ (calculated for C_36_H_55_O_11_, *m/z* 663.3739); ^1^H and ^13^C NMR data see Table 1.

Vernonin Q (**3**)

White amorphous solid; ${\left[\alpha \right]}_{D}^{26}$ − 5.79 (*c* 2.42, CH_3_OH); UV (CH_3_OH) *λ*_max_ (log *ε*) = 235 (4.07), 242 (4.12) and 250 (3.94) nm; ECD (CH_3_OH) *λ*_max_ (Δ*ε*) 239 (+1.68) nm; HR-ESI-MS *m/z* 717.3817 [M + Na]^+^ (calculated for C_37_H_58_O_12_Na, *m/z* 717.3820); ^1^H and ^13^C NMR data see Table 1.

Vernonin R (**4**)

White amorphous solid; ${\left[\alpha \right]}_{D}^{26}$ − 0.55 (*c* 7.30, CH_3_OH); UV (CH_3_OH) *λ*_max_ (log *ε*) = 235 (4.10), 242 (4.16) and 250 (3.97) nm; ECD (CH_3_OH) *λ*_max_ (Δ*ε*) 239 (+7.89) nm; HR-ESI-MS *m/z* 701.3868 [M + Na]^+^ (calculated for C_37_H_58_O_11_Na, *m/z* 701.3871); ^1^H and ^13^C NMR data see Table 1.

Vernonin S (**5**)

White amorphous solid; ${\left[\alpha \right]}_{D}^{26}$ + 20.61 (*c* 5.92, CH_3_OH); UV (CH_3_OH) *λ*_max_ (log *ε*) = 235 (4.09), 242 (4.15) and 250 (3.97) nm; ECD (CH_3_OH) *λ*_max_ (Δ*ε*) 238 (+1.74) nm; HR-ESI-MS *m/z* 659.3767 [M + Na]^+^ (calculated for C_35_H_56_O_10_Na, *m/z* 659.3766); ^1^H and ^13^C NMR data see Table 2.

Vernonin T (**6**)

White amorphous solid; ${\left[\alpha \right]}_{D}^{26}$ + 27.64 (*c* 7.67, CH_3_OH); UV (CH_3_OH) *λ*_max_ (log *ε*) = 235 (4.09), 242 (4.14) and 250 (3.96) nm; ECD (CH_3_OH) *λ*_max_ (Δ*ε*) 239 (+2.49) nm; HR-ESI-MS *m/z* 659.3765 [M + Na]^+^ (calculated for C_35_H_56_O_10_Na, *m/z* 659.3766); ^1^H and ^13^C NMR data see Table 2.

Vernonin O (**7**)

White amorphous solid; ${\left[\alpha \right]}_{D}^{25}$ + 11.43 (*c* 4.19, CH_3_OH); UV (CH_3_OH) *λ*_max_ (log *ε*) = 235 (3.92), 242 (3.98) and 250 (3.80) nm; ECD (CH_3_OH) *λ*_max_ (Δ*ε*) 242 (+4.47) nm; HR-ESI-MS *m/z* 539.3341 [M + Na]^+^ (calculated for C_31_H_48_O_6_Na, *m/z* 539.3343); ^1^H and ^13^C NMR data see Table 2.

Vernonin N (**8**)

White amorphous solid; ${\left[\alpha \right]}_{D}^{30}$ + 42.20 (*c* 2.42, CH_3_OH); UV (CH_3_OH) *λ*_max_ (log *ε*) = 235 (4.07), 242 (4.12) and 250 (3.94) nm; ECD (CH_3_OH) *λ*_max_ (Δ*ε*) 242 (+3.81) nm; HR-ESI-MS *m/z* 497.3241 [M + Na]^+^ (calculated for C_29_H_46_O_5_Na, *m/z* 497.3237); ^1^H and ^13^C NMR data see Table 2.

### 3.4. X-ray Diffraction Analysis of Compound ***1***

Compound **1** was crystallized from CH_3_OH solvents. Crystallographic data was collected on Rigaku Oxford Diffraction SuperNova Dual, Cu at zero, AtlasS2, using Cu K*α* radiation at 100.00 (10) K. The structure was elucidated using Oxle2 software and refined by SHELXL-2017. Crystallographic data of 1 was deposited at the Cambridge Crystallographic Data Center (CCDC 2191187).

Crystal data for Vvernonin M (**1**)

C_36_H_56_O_10_ (M = 648.80 g/mol): monoclinic, space group P2_1_ (no. 4), *a* = 16.217(4) Å, *b* = 5.6615(13) Å, *c* = 20.908(6) Å, *β* = 106.92(3)°, *V* = 1836.5(9) Å^3^, *Z* = 2, *T* = 100.00(10) K, μ(CuK*α*) = 0.687 mm^−1^, *Dcalc* = 1.170 g/cm^3^, 6613 reflections measured (4.418° ≤ 2Θ ≤ 147.734°), 5110 unique (*R*_int_ = 0.0915, *R*_sigma_ = 0.1004) which were used in all calculations. The final *R*_1_ was 0.1051 (I > 2σ(I)) and *wR*_2_ was 0.3058 (all data). Flack parameter = 0.40(6).

### 3.5. Computational Electronic Circular Dichroism (ECD) Spectrum Calculation Method

The detailed calculation method of the ECD spectrum of **1** was supplied in supporting information.

### 3.6. Snatzke’s Method to Identify the Absolute Configuration of the Vic-Diols in 7

The detailed operation procedures of Snatzke’s method were performed as previously described [29]. The Mo_2_(OAc)_4_ and **7** were mixed in DMSO solvent to produce Mo_2_^4+^ complex. The stable ICD of the curve was recorded in a CD spectrum. The cotton effects at 310 nm or 400 nm were used as a reference for diagnostic bands.

### 3.7. Cell Culture

BV-2 microglia cells were cultured in DMEM medium (BasalMedia, China) with 10% fetal bovine serum (BI) in a cell culture incubator with 5% CO_2_ and 37 °C.

### 3.8. Cell Proliferation Assay

Cell viability was evaluated through MTT assay. BV-2 cells were seeded in 96-well plates at a density of 8 × 10^3^ cells per well. After 16 h, the cells were treated with compounds or DMSO for 24 h. Before culturing with DMEM medium (60 μL) and MTT reagent (15 μL), the cells were incubated with LPS (1 μg/mL). After 4 h, each plate dissolved in equal amounts of DMSO (100 μL). The optical absorbance was measured at 492 nm through a microplate reader (Thermo Multiskan MK3, Thermo Scientific, Helsinki, Finland).

### 3.9. Western Blot Analysis

BV-2 cells were lysed by an ice-cold buffer (RIPA) containing protease-inhibitor and phosphatase-inhibitor cocktails (MCE). Protein samples of cell lysate were separated through sodium dodecyl sulfate-polyacrylamide gel electrophoresis (SDS-PAGE) and transfected into polyvinylidene difluoride (PVDF) membranes. The membranes were blocked in 5% non-fat milk at room temperature for 1 h. Then, the membranes were incubated with primary antibodies at 4 °C overnight. After being washed three times with TBST buffer, the membranes were incubated with the second antibodies at room temperature for one hour. Then, the membranes were washed four times and immunoreactive products were visualized via ECL western blotting detection reagents.

## 4. Conclusions

In this paper, eight undescribed polyhydric stigmastane-type steroids derivative were purified from *Vernonia amygdalina*. Their gross structures and stereochemistry were elucidated by comprehensive NMR spectra and X-ray diffraction. Moreover, ECD calculation and Snatzke’s method were also employed for the determination of their absolute configuration. The anti-neuroinflammatory activity results showed that compound **1** restrained the activation of NF-κB through suppressing the degradation of IκB proteins in BV-2 microglia cells. In addition, compound **1** could inhibit the activation of PI3K/AKT and p38 MAPK pathway in BV-2 microglia cells. Therefore, compound **1** could be a candidate for treatment of neuroinflammatory. Despite the anti-neuroinflammatory activity of compound **1** has been confirmed in vitro, however, the presence of blood brain barrier in vivo must be considered. Therefore, further researches should be paid attention to improve bioavailability of active compounds.

## Figures and Tables

**Table 1:** ^1^H (600 MHz) and ^13^C NMR (150 MHz) data for compounds **1**–**4** (*δ* in ppm, *J* in Hz).

No.	1 ^a^		2 ^a^		3 ^b^		4 ^a^	
	*δ*_C_	*δ*_H_	*δ*_C_	*δ*_H_	*δ*_C_	*δ*_H_	*δ*_C_	*δ*_H_
1α	35.3	1.27, m	35.3	1.21, overlap	35.3	1.23, overlap	35.2	1.24, m
1β		1.87, m		1.84, overlap		1.83, m		1.87, m
2α	30.5	2.18, m	30.5	2.16, br d (8.0)	30.5	2.15, overlap	30.4	2.15, m
2β		1.73, m		1.72, m		1.70, m		1.72, br d (13.3)
3	77.5	3.99, overlap	77.5	3.98, m	77.4	3.97, m	77.4	3.98, m
4α	34.9	2.07, m	34.9	2.07, br d (12.4)	34.9	2.03, br d (11.4)	34.8	2.04, m
4β		1.43, d (11.8)		1.43, m		1.40, q (11.7)		1.41, q (12.1)
5	39.7	1.39, m	39.7	1.38, m	39.6	1.32, m	39.5	1.34, m
6α	30.5	1.84, m	30.5	1.85, m	30.5	1.80, m	30.5	1.81, m
6β		1.84, m		1.85, m		1.80, m		1.81, m
7	121.6	5.42, br s	121.5	5.44, br s	121.9	5.31, br s	121.7	5.31, br s
8	135.2		135.3		135.5		135.6	
9	144.5		144.4		144.6		144.4	
10	36.7		36.6		36.5		36.5	
11	119.0	5.45, br d (5.8)	119.2	5.46, overlap	118.7	5.42, br s	118.8	5.43, br d (6.8)
12α	43.6	2.11, d (6.3)	43.3	2.37, m	42.2	2.32, overlap	43.1	2.27, br d (16.9)
12β		2.15, overlap		2.44, m		2.32, overlap		2.34, dd (17.2, 6.4)
13	42.0		42.2		43.4		43.4	
14	47.3	2.35, br d (13.5)	47.5	2.45, m	49.6	2.54, m	49.6	2.52, m
15α	32.8	2.43, dd (12.8, 5.6)	33.1	2.50, m	34.5	1.89, m	34.6	1.86, m
15β		1.51, m		1.61, t (12.9)		2.10, overlap		2.06, m
16	111.0		111.5		79.5	5.55, br s	80.1	5.40, t (7.1)
17	61.6	1.34, d (11.8)	54.5	2.26, m	57.0	2.32, overlap	61.0	2.19, dd (10.7, 6.2)
18	13.7	0.60, s	14.2	0.71, s	13.3	0.66, s	13.0	0.64, s
19	19.9	0.81, s	19.8	0.82, s	19.8	0.79, s	19.8	0.81, s
20	33.7	2.17, overlap	41.0	2.35, m	40.6	2.73, br s	35.4	2.48, m
21a	18.2	1.15, d (6.3)	60.2	4.43, m	61.8	4.25, m	16.2	1.45, d (5.6)
21b				3.89, m		4.19, m		
22	80.1	3.97, dd (10.3, 7.6)	74.8	4.71, m	79.0	4.52, br d (6.4)	80.4	4.29, m
23	76.1	4.18, d (7.4)	76.1	4.24, br d (6.8)	76.9	4.36, m	76.0	4.35, t (5.6)
24	83.3		83.5		83.5		82.7	
25	33.5	1.86, m	33.5	1.89, m	32.8	1.89, m	32.4	1.94, m
26	17.9	1.24, d (6.7)	17.9	1.26, d (5.9)	18.1	1.27, d (6.4)	17.9	1.19, d (5.9)
27	18.2	1.07, d (6.5)	18.2	1.08, d (5.7)	18.3	1.10, d (6.1)	18.3	1.12, d (6.4)
28	82.2	4.36, overlap	82.2	4.40, m	81.8	4.39, m	81.2	4.41, m
29	17.3	1.21, d (6.7)	17.2	1.20, d (5.8)	15.3	1.24, d (6.5)	15.4	1.29, d (6.7)
16-OMe	50.5	3.35, s	50.6	3.37, s				
16-OAc					171.0		171.0	
					21.8	2.13, s	21.8	2.15, s
1′	102.8	5.04, d (7.7)	102.8	5.02, overlap	102.7	5.03, overlap	102.7	5.02, d (7.7)
2′	75.8	4.07, t (7.9)	75.7	4.07, m	75.8	4.07, m	75.7	4.05, t (7.9)
3′	79.1	4.31, overlap	79.0	4.31, m	79.1	4.31, m	79.0	4.31, m
4′	72.1	4.29, overlap	72.1	4.28, m	72.2	4.28, m	72.1	4.28, m
5′	78.9	4.01, m	78.8	4.01, m	78.9	4.01, m	78.9	4.00, m
6′	63.3	4.59, br d (11.5)	63.2	4.59, br d (11.3)	63.3	4.60, br d (11.6)	63.2	4.58, br d (11.5)
		4.43, dd (11.6, 5.1)		4.43, m		4.42, dd (11.8, 5.2)		4.41, m

^a^ Recorded in pyridine-*d*_5_; ^b^ recorded in CD_3_OD.

**Table 2:** ^1^H (600 MHz) and ^13^C NMR (150 MHz) data for compounds **5**–**8** (*δ* in ppm, *J* in Hz).

No.	5 ^a^		6 ^a^		7 ^a^		8 ^a^	
	*δ*_C_	*δ*_H_	*δ*_C_	*δ*_H_	*δ*_C_	*δ*_H_	*δ*_C_	*δ*_H_
1α	35.3	1.24, dd (13.6,3.4)	35.3	1.26, td (13.3, 3.0)	35.9	1.34, td (13.6, 3.5)	35.6	1.39, t (13.6)
1β		1.86, dt (13.4, 3.1)		1.89, overlap		1.99, overlap		1.97, m
2α	30.5	2.14, m	30.5	2.15, m	32.2	1.84, m	33.0	2.12, m
2β		1.71, m		1.72, br d (12.8)		1.48, m		1.77, m
3	77.4	3.95, m	77.4	3.97, m	71.4	3.51, m	70.6	3.84, m
4α	34.8	2.01, m	34.8	2.03, br d (11.3)	38.4	1.70, m	39.2	1.97, m
4β		1.41, q (12.5)		1.41, q (12.0)		1.29, m		1.60, q (12.5)
5	39.5	1.30, overlap	39.6	1.32, m	40.6	1.42, m	40.0	1.49, overlap
6α	30.6	1.80, m	30.6	1.82, m	30.9	1.91, m	30.7	1.89, m
6β		1.80, m		1.82, m		1.91, m		1.89, m
7	121.3	5.39, br s	120.9	5.43, br s	122.1	5.39, br s	121.5	5.45, br s
8	136.5		136.6		136.3		136.5	
9	144.5		144.7		145.2		144.7	
10	36.5		36.5		37.0		36.0	
11	119.0	5.43, br d (6.0)	119.2	5.46, br d (6.0)	119.3	5.52, br d (6.4)	119.0	5.51, br d (5.4)
12α	43.3	2.29, br d (16.8)	43.5	2.23, br d (16.6)	43.6	2.25, br d (17.1)	43.4	2.34, br d (17.0)
12β		2.36, dd (17.2, 6.6)		2.39, dd (17.2, 6.6)		2.35, dd (17.2, 6.7)		2.41, dd (17.2, 6.3)
13	44.3		43.4		44.0		44.3	
14	49.6	2.77, m	50.5	2.20, overlap	50.1	2.44, m	49.7	2.82, m
15α	37.1	2.08, m	37.2	1.88, overlap	34.8	1.70, m	37.2	2.10, m
15β		2.14, m		2.54, dt (12.8, 7.7)		1.97, m		2.18, mk
16	76.5	4.58, overlap	72.3	4.87, br s	81.0	5.00, t (7.0)	76.5	4.59, br s
17	65.2	2.12, overlap	60.1	1.97, dd (10.8, 7.4)	61.3	1.85, m	65.3	2.15, m
18	13.4	0.69, s	13.3	1.05, s	12.9	0.61, s	13.5	0.73, s
19	19.8	0.83, s	20.0	0.86, s	19.9	0.91, s	20.1	0.97, s
20	35.5	2.60, m	31.7	3.10, m	35.4	2.06, m	35.6	2.62, m
21a	16.6	1.47, d (6.7)	15.8	1.52, d (6.7)	15.7	1.07, d (6.7)	16.6	1.50, d (6.7)
21b								
22	81.2	4.84, br s	82.0	4.60, br d (6.0)	80.7	3.93, dd (7.1, 1.4)	81.3	4.87, br s
23	76.3	4.51, t (6.0)	75.9	4.44, t (4.9)	76.4	4.03, d (7.1)	76.3	4.52, t (5.3)
24	83.0		82.6		83.1		83.1	
25	32.4	1.95, m	32.3	1.92, m	32.9	1.81, m	32.5	1.95, m
26	18.1	1.21, d (6.8)	17.9	1.14, d (6.8)	17.4	0.95, d (6.4)	18.1	1.22, d (6.8)
27	18.4	1.12, d (6.6)	18.5	1.10, d (6.5)	17.9	0.94, d (6.4)	18.5	1.13, d (6.5)
28	81.2	4.42, q (6.7)	81.3	4.43, q (7.1)	81.8	3.97, q (6.7)	81.3	4.43, q (6.4)
29	15.5	1.12, d (6.7)	15.6	1.28, d (6.7)	15.1	1.12, d (6.7)	15.5	1.33, d (6.7)
16-OAc					172.7			
					21.5	2.00, s		
1′	102.6	5.02, overlap	102.6	5.02, d (7.7)				
2′	75.7	4.04, t (8.0)	75.7	4.04, t (8.1)				
3′	79.0	4.29, m	79.0	4.29, m				
4′	72.1	4.27, m	72.1	4.26, m				
5′	78.8	3.98, m	78.8	3.99, m				
6′	63.2	4.57, br d (10.9)	63.2	4.57, br d (10.9)				
		4.40, m		4.40, dd (11.6, 5.2)				

^a^ Recorded in pyridine-*d*_5_.

## Data Availability

Data is contained within the article and Supplementary Materials.

## Supplementary Materials

The following supporting information can be downloaded at: https://www.mdpi.com/article/10.3390/ph15091160/s1, Figure S1: Optimized geometries of predominant conformers for compound **1**; Table S1: Cartesian coordinates for the optimized conformers of 1; Spectral information of **1–8**.
