# Supplementary material for: Polyhydric Stigmastane-Type Steroids Derivative from Vernonia amygdalina and Their Anti-Neuroinflammatory Activity
Source: Pharmaceuticals (Basel). 2022 Sep 19;15(9):1160. doi: 10.3390/ph15091160 (PMC9506346; doi:10.3390/ph15091160)

Polyhydric Stigmastane-type Steroids Derivative from *Vernonia amygdalina* and Their Anti-neuroinflammatory Activity

Xiangzhong Liu,† Mi Zhou,† Shoulun He, Qiannan Xu, Chunchun Du, Honghong Zhu, Ting Lin, Guanghui Wang, Wenjing Tian* and Haifeng Chen*

Fujian Provincial Key Laboratory of Innovative Drug Target, School of Pharmaceutical Sciences, Xiamen University, Xiamen 361005, China

* Correspondence: tianwj@xmu.edu.cn (Wenjing Tian); haifeng@xmu.edu.cn (Haifeng Chen)

† These authors contributed equally to this work.

List of Supporting Information

1. Figure S1. Optimized geometries of predominant conformers for compound **1**...........................4
2. Table S1. Cartesian coordinates for the optimized conformers of **1**. 4-14
3. Spectral information of **1** 15-19
3.1 HR-ESI-MS and UV (CH_3OH) spectrum of **1** 15-16
3.2 1D and 2D NMR spectra of **1** in pyridine-d_5 17-19
3.2.1 ^1H NMR spectrum of **1** in pyridine-d_5 17
3.2.2 ^{13}C NMR spectrum of **1** in pyridine-d_5 17
3.2.3 $^1H–^1H$ COSY spectrum of **1** in pyridine-d_5...18
3.2.4 HSQC spectrum of **1** in pyridine-d_5...19
3.2.5 HMBC spectrum of **1** in pyridine-d_5...19
3.2.6 NOESY spectrum of **1** in pyridine-d_5...19
4. Spectral information of **2** 20-25
4.1 HR-ESI-MS and UV (CH_3OH) spectrum of **2** 20-21
4.2 1D and 2D NMR spectra of **2** in pyridine-d_5 22-25
4.2.1 ^1H NMR spectrum of **2** in pyridine-d_5 22
4.2.2 ^{13}C NMR spectrum of **2** in pyridine-d_5 22
4.2.3 DEPT-135 spectrum of **3** in pyridine-d_5...23
4.2.4 $^1H–^1H$ COSY spectrum of **2** in pyridine-d_5...23
4.2.5 HSQC spectrum of **2** in pyridine-d_5...24
4.2.6 HMBC spectrum of **2** in pyridine-d_5...24
4.2.7 NOESY spectrum of **2** in pyridine-d_5...25
5. Spectral information of **3** 26-31
5.1 HR-ESI-MS and UV (CH_3OH) spectrum of **3** 26-27
5.2 1D and 2D NMR spectra of **3** in pyridine-d_5 28-31
5.2.1 ^1H NMR spectrum of **3** in pyridine-d_5 28
5.2.2 ^{13}C NMR spectrum of **3** in pyridine-d_5 28
5.2.3 DEPT-135 spectrum of **3** in pyridine-d_5...29
5.2.4 $^1H–^1H$ COSY spectrum of **3** in pyridine-d_5...29
5.2.5 HSQC spectrum of **3** in pyridine-d_5...30
5.2.6 HMBC spectrum of **3** in pyridine-d_5...30
5.2.7 NOESY spectrum of **3** in pyridine-d_5...31
6. Spectral information of **4** 32-37
6.1 HR-ESI-MS and UV (CH_3OH) spectrum of **4** 32-33
6.2 1D and 2D NMR spectra of **4** in pyridine-d_5 34-37
6.2.1 ^1H NMR spectrum of **4** in pyridine-d_5 34
6.2.2 ^{13}C NMR spectrum of **4** in pyridine-d_5 34
6.2.3 DEPT-135 spectrum of **4** in pyridine-d_5...35
6.2.4 $^1H–^1H$ COSY spectrum of **4** in pyridine-d_5...35
6.2.5 HSQC spectrum of **4** in pyridine-d_5...36
6.2.6 HMBC spectrum of **4** in pyridine-d_5...36
6.2.7 NOESY spectrum of **4** in pyridine-d_5...37
7. Spectral information of **5** 38-43

7.1 HR-ESI-MS and UV (CH_3OH) spectrum of **5** 38-39
7.2 1D and 2D NMR spectra of **5** in pyridine-d_5 40-43
7.2.1 ^1H NMR spectrum of **5** in pyridine-d_5 40
7.2.2 ^{13}C NMR spectrum of **5** in pyridine-d_5 40
7.2.3 DEPT-135 spectrum of **5** in pyridine-d_5 41
7.2.4 ^1H–^1H COSY spectrum of **5** in pyridine-d_5 41
7.2.5 HSQC spectrum of **5** in pyridine-d_5 42
7.2.6 HMBC spectrum of **5** in pyridine-d_5 42
7.2.7 NOESY spectrum of **5** in pyridine-d_5 43
8. Spectral information of **6** 44-49
8.1 HR-ESI-MS and UV (CH_3OH) spectrum of **6** 44-45
8.2 1D and 2D NMR spectra of **6** in pyridine-d_5 46-49
8.2.1 ^1H NMR spectrum of **6** in pyridine-d_5 46
8.2.2 ^{13}C NMR spectrum of **6** in pyridine-d_5 46
8.2.3 DEPT-135 spectrum of **6** in pyridine-d_5 47
8.2.4 ^1H–^1H COSY spectrum of **6** in pyridine-d_5 47
8.2.5 HSQC spectrum of **6** in pyridine-d_5 48
8.2.6 HMBC spectrum of **6** in pyridine-d_5 48
8.2.7 NOESY spectrum of **6** in pyridine-d_5 49
9. Spectral information of **7** 50-55
9.1 HR-ESI-MS and UV (CH_3OH) spectrum of **7** 50-51
9.2 1D and 2D NMR spectra of **7** in CD_3OD 52-55
9.2.1 ^1H NMR spectrum of **7** in CD_3OD 52
9.2.2 ^{13}C NMR spectrum of **7** in CD_3OD 52
9.2.3 DEPT-135 spectrum of **7** in CD_3OD 53
9.2.4 ^1H–^1H COSY spectrum of **7** in CD_3OD 53
9.2.5 HSQC spectrum of **7** in CD_3OD 54
9.2.6 HMBC spectrum of **7** in CD_3OD 54
9.2.7 NOESY spectrum of **7** in CD_3OD 55
10. Spectral information of **8** 56-61
10.1 HR-ESI-MS and UV (CH_3OH) spectrum of **8** 56-57
10.2 1D and 2D NMR spectra of **8** in pyridine-d_5 58-61
10.2.1 ^1H NMR spectrum of **8** in pyridine-d_5 58
10.2.2 ^{13}C NMR spectrum of **8** in pyridine-d_5 58
10.2.3 DEPT-135 spectrum of **8** in pyridine-d_5 59
10.2.4 ^1H–^1H COSY spectrum of **8** in pyridine-d_5 59
10.2.5 HSQC spectrum of **8** in pyridine-d_5 60
10.2.6 HMBC spectrum of **8** in pyridine-d_5 60
10.2.7 NOESY spectrum of **8** in pyridine-d_5 61

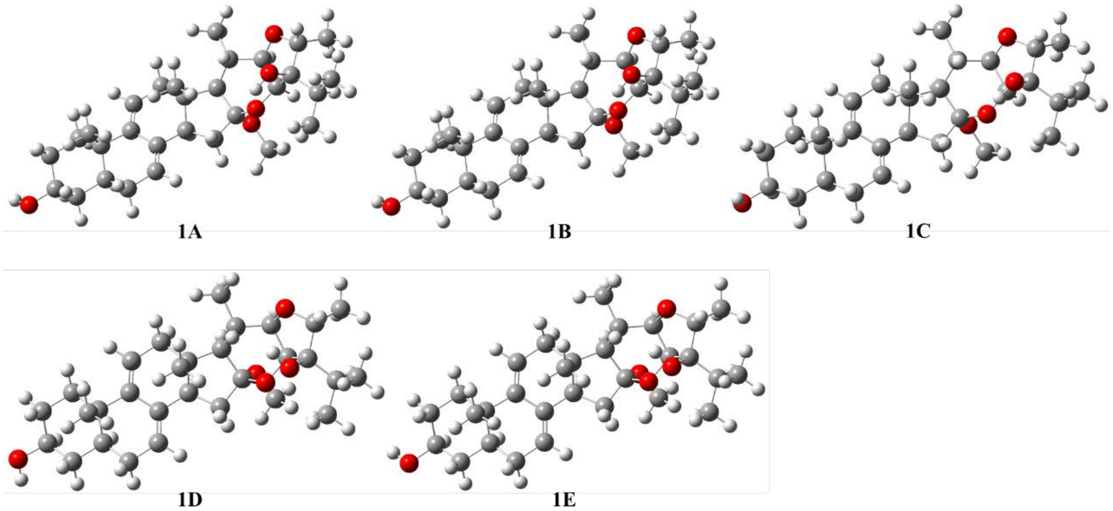

Figure S1. Optimized geometries of predominant conformers for compound **1**.

Table S1. Cartesian coordinates for the optimized conformers of **1**

Cartesian coordinates of optimized compound **1A**

Center Number	Atomic Number	Atomic Type	Coordinates (Angstroms)		
			X	Y	Z
1	6	0	-9.8089	-1.3551	-1.50811
2	6	0	-9.55105	-2.74076	-2.10574
3	6	0	-8.47955	-3.48168	-1.31187
4	6	0	-7.18384	-2.6612	-1.20798
5	6	0	-7.39804	-1.24733	-0.58243
6	6	0	-8.51309	-0.53331	-1.39164
7	6	0	-6.06602	-3.431	-0.49286
8	6	0	-4.81453	-2.60331	-0.40388
9	6	0	-4.81192	-1.25993	-0.52175
10	6	0	-6.06413	-0.47619	-0.63488
11	6	0	-3.54751	-0.44429	-0.64262
12	6	0	-3.6058	0.905025	0.124606
13	6	0	-4.75561	1.71039	-0.51195
14	6	0	-6.01061	0.874961	-0.66206
15	6	0	-2.19175	-1.03516	-0.25529
16	6	0	-1.26561	0.194563	-0.3402
17	6	0	-2.16484	1.463724	-0.13669
18	8	0	-0.25608	0.066697	0.66124
19	6	0	0.660152	1.167767	0.713787
20	6	0	-0.0506	2.544802	0.69184
21	6	0	-1.56716	2.433598	0.902275
22	6	0	1.429962	1.141668	2.062732
23	6	0	1.699767	2.65523	2.264689
24	8	0	0.523137	3.30775	1.763072

25	6	0	-7.81043	-1.33859	0.912308
26	8	0	-10.7276	-3.55968	-2.10204
27	6	0	-2.21675	3.823867	0.863532
28	6	0	2.939966	3.229561	1.570291
29	6	0	2.696488	0.245356	2.08236
30	6	0	3.416374	0.323009	3.440906
31	6	0	2.3928	-1.22496	1.746456
32	8	0	0.53324	0.750385	3.107928
33	1	0	-6.82806	-2.48247	-2.23513
34	1	0	-3.47319	-0.16925	-1.70821
35	6	0	-3.89029	0.692869	1.627207
36	1	0	-1.69443	2.019484	1.907259
37	1	0	-2.17598	1.971863	-1.10875
38	1	0	0.150681	3.058978	-0.25978
39	1	0	1.366172	1.083673	-0.11984
40	8	0	-0.66508	0.357143	-1.62356
41	6	0	0.171094	-0.69747	-2.09584
42	1	0	-10.2797	-1.48478	-0.52594
43	1	0	-10.5295	-0.80696	-2.1312
44	1	0	-9.19725	-2.61996	-3.14402
45	1	0	-8.27091	-4.44213	-1.79851
46	1	0	-8.88031	-3.71648	-0.31777
47	1	0	-8.75318	0.433859	-0.93705
48	1	0	-8.12966	-0.31763	-2.39815
49	1	0	-6.39936	-3.75297	0.50626
50	1	0	-5.85176	-4.35861	-1.04052
51	1	0	-3.87033	-3.13226	-0.29336
52	1	0	-4.99591	2.600406	0.083473
53	1	0	-4.44065	2.088354	-1.49743
54	1	0	-6.93462	1.439885	-0.75129
55	1	0	-1.85973	-1.84567	-0.90748
56	1	0	-2.19739	-1.41937	0.768879
57	1	0	1.737304	2.870118	3.336828
58	1	0	-8.03434	-0.33705	1.294011
59	1	0	-7.00315	-1.74839	1.526155
60	1	0	-8.69543	-1.96282	1.065446
61	1	0	-11.4236	-3.10864	-2.59816
62	1	0	-3.2824	3.778702	1.107232
63	1	0	-1.73296	4.487476	1.585127
64	1	0	-2.12029	4.273734	-0.13281
65	1	0	2.926737	4.317392	1.682812
66	1	0	3.86951	2.852985	2.006635
67	1	0	2.949398	3.005393	0.497948

68	1	0	3.372828	0.628665	1.306182
69	1	0	4.320073	-0.29603	3.426608
70	1	0	3.718403	1.342017	3.701376
71	1	0	2.762654	-0.04391	4.237768
72	1	0	3.320766	-1.80719	1.749044
73	1	0	1.923503	-1.34049	0.766039
74	1	0	1.723376	-1.66795	2.491815
75	1	0	-0.08376	0.108222	2.725173
76	1	0	-4.83616	0.166001	1.773325
77	1	0	-3.10706	0.111911	2.122533
78	1	0	-3.96364	1.652388	2.148235
79	1	0	0.745547	-0.27946	-2.92561
80	1	0	0.858498	-1.05022	-1.31933
81	1	0	-0.4149	-1.54682	-2.4684

Cartesian coordinates of optimized compound **1B**

Center Number	Atomic Number	Atomic Type	Coordinates (Angstroms)		
			X	Y	Z
1	6	0	-9.85118	-1.21729	-1.51194
2	6	0	-9.61539	-2.59953	-2.1117
3	6	0	-8.55235	-3.36287	-1.316
4	6	0	-7.24105	-2.56614	-1.21126
5	6	0	-7.43656	-1.15216	-0.58044
6	6	0	-8.54199	-0.42	-1.38715
7	6	0	-6.13338	-3.35511	-0.50111
8	6	0	-4.86995	-2.54546	-0.41211
9	6	0	-4.84974	-1.20169	-0.52369
10	6	0	-6.09138	-0.40041	-0.63
11	6	0	-3.57463	-0.40286	-0.64365
12	6	0	-3.61233	0.942317	0.132039
13	6	0	-4.75275	1.767247	-0.49634
14	6	0	-6.01917	0.949954	-0.64946
15	6	0	-2.22591	-1.01461	-0.26419
16	6	0	-1.28331	0.202937	-0.34417
17	6	0	-2.16454	1.482844	-0.12978
18	8	0	-0.27247	0.054804	0.653076
19	6	0	0.658729	1.143151	0.710041
20	6	0	-0.03353	2.529739	0.699846
21	6	0	-1.55085	2.437567	0.913824
22	6	0	1.432545	1.097422	2.056165
23	6	0	1.722871	2.605889	2.267803
24	8	0	0.553222	3.277204	1.77489
25	6	0	-7.85007	-1.2437	0.913925

26	8	0	-10.8767	-3.28056	-2.12253
27	6	0	-2.18166	3.836743	0.886108
28	6	0	2.968144	3.168824	1.57312
29	6	0	2.687321	0.184642	2.065373
30	6	0	3.412643	0.243674	3.421973
31	6	0	2.36346	-1.27932	1.720578
32	8	0	0.534203	0.710436	3.101568
33	1	0	-6.88552	-2.3888	-2.23872
34	1	0	-3.5	-0.12233	-1.70776
35	6	0	-3.8958	0.724651	1.634066
36	1	0	-1.6809	2.018518	1.916403
37	1	0	-2.17142	1.997483	-1.09844
38	1	0	0.172101	3.047916	-0.24867
39	1	0	1.360736	1.05527	-0.12656
40	8	0	-0.68474	0.365418	-1.62848
41	6	0	0.136155	-0.69704	-2.10976
42	1	0	-10.3285	-1.34381	-0.53264
43	1	0	-10.5641	-0.66793	-2.13707
44	1	0	-9.26065	-2.47746	-3.1495
45	1	0	-8.34666	-4.32842	-1.80119
46	1	0	-8.95596	-3.5921	-0.32168
47	1	0	-8.76856	0.54751	-0.92653
48	1	0	-8.15329	-0.20242	-2.39126
49	1	0	-6.46954	-3.67523	0.497663
50	1	0	-5.93289	-4.28392	-1.05243
51	1	0	-3.93292	-3.08795	-0.30671
52	1	0	-4.97989	2.656331	0.105573
53	1	0	-4.43523	2.147706	-1.48003
54	1	0	-6.93553	1.527727	-0.73472
55	1	0	-1.90685	-1.82541	-0.92249
56	1	0	-2.23376	-1.4051	0.757591
57	1	0	1.766867	2.812686	3.341284
58	1	0	-8.05411	-0.23985	1.300334
59	1	0	-7.05132	-1.67258	1.526093
60	1	0	-8.74838	-1.84945	1.063771
61	1	0	-10.7536	-4.16116	-2.50113
62	1	0	-3.24706	3.804558	1.132849
63	1	0	-1.68672	4.489028	1.610454
64	1	0	-2.08212	4.291705	-0.10761
65	1	0	3.894174	2.777251	2.003695
66	1	0	2.971117	2.951973	0.499233
67	1	0	2.969415	4.25593	1.693137
68	1	0	3.366035	0.564412	1.289533

69	1	0	4.308078	-0.38707	3.400521
70	1	0	3.72891	1.256858	3.68818
71	1	0	2.756758	-0.11992	4.218581
72	1	0	3.28382	-1.87349	1.715972
73	1	0	1.889357	-1.38214	0.741044
74	1	0	1.690921	-1.71864	2.465307
75	1	0	-0.09299	0.079889	2.716098
76	1	0	-4.84879	0.210556	1.779429
77	1	0	-3.11968	0.12941	2.12362
78	1	0	-3.95407	1.681766	2.161338
79	1	0	0.713048	-0.28162	-2.93914
80	1	0	0.821643	-1.06309	-1.33773
81	1	0	-0.46196	-1.53667	-2.48515

Cartesian coordinates of optimized compound **1C**

Center Number	Atomic Number	Atomic Type	Coordinates (Angstroms)		
			X	Y	Z
1	6	0	-9.80612	-1.28235	-1.6408
2	6	0	-9.55283	-2.66768	-2.24066
3	6	0	-8.49323	-3.42183	-1.4321
4	6	0	-7.19181	-2.61222	-1.30223
5	6	0	-7.40562	-1.20006	-0.67297
6	6	0	-8.50486	-0.47388	-1.49327
7	6	0	-6.08818	-3.39356	-0.5775
8	6	0	-4.83238	-2.57465	-0.46851
9	6	0	-4.81994	-1.2307	-0.57897
10	6	0	-6.06552	-0.43813	-0.70316
11	6	0	-3.54897	-0.4227	-0.68027
12	6	0	-3.60755	0.92236	0.09437
13	6	0	-4.74455	1.738994	-0.55072
14	6	0	-6.00287	0.912766	-0.72146
15	6	0	-2.20161	-1.02471	-0.28109
16	6	0	-1.26669	0.199552	-0.34798
17	6	0	-2.16005	1.473219	-0.14667
18	8	0	-0.26945	0.058885	0.663902
19	6	0	0.653137	1.153788	0.733824
20	6	0	-0.04872	2.535428	0.713183
21	6	0	-1.56829	2.432512	0.905406
22	6	0	1.40782	1.113951	2.09096
23	6	0	1.684432	2.624465	2.306121
24	8	0	0.517161	3.287319	1.796282
25	6	0	-7.83747	-1.29544	0.81595
26	8	0	-10.7607	-3.42319	-2.38732

27	6	0	-2.20859	3.827149	0.868275
28	6	0	2.935468	3.19593	1.629041
29	6	0	2.668668	0.209913	2.11853
30	6	0	3.374089	0.274374	3.485324
31	6	0	2.359963	-1.25632	1.769595
32	8	0	0.497188	0.72112	3.123522
33	1	0	-6.82305	-2.43139	-2.32429
34	1	0	-3.46078	-0.14189	-1.74327
35	6	0	-3.91095	0.703013	1.592209
36	1	0	-1.7097	2.012733	1.906156
37	1	0	-2.15662	1.987601	-1.11548
38	1	0	0.166775	3.054783	-0.23248
39	1	0	1.367666	1.070616	-0.09259
40	8	0	-0.65102	0.366149	-1.62348
41	6	0	0.183843	-0.69072	-2.09318
42	1	0	-10.2915	-1.40283	-0.66126
43	1	0	-10.5152	-0.73684	-2.27374
44	1	0	-9.19111	-2.54745	-3.26993
45	1	0	-8.28504	-4.38242	-1.91883
46	1	0	-8.89897	-3.65795	-0.43705
47	1	0	-8.74393	0.492221	-1.03577
48	1	0	-8.10559	-0.25403	-2.49256
49	1	0	-6.43591	-3.71849	0.415923
50	1	0	-5.87335	-4.31957	-1.12764
51	1	0	-3.89309	-3.11046	-0.35003
52	1	0	-4.9865	2.626823	0.047257
53	1	0	-4.41574	2.121041	-1.53005
54	1	0	-6.92185	1.484255	-0.81997
55	1	0	-1.86729	-1.83336	-0.9344
56	1	0	-2.22133	-1.41496	0.740634
57	1	0	1.711595	2.831942	3.380026
58	1	0	-8.05753	-0.29408	1.200095
59	1	0	-7.04235	-1.71615	1.438234
60	1	0	-8.73056	-1.91179	0.955508
61	1	0	-11.1456	-3.56193	-1.51075
62	1	0	-3.27726	3.787375	1.099375
63	1	0	-1.72897	4.482934	1.59973
64	1	0	-2.09775	4.282865	-0.12389
65	1	0	2.927539	4.283037	1.748784
66	1	0	3.857962	2.810818	2.072818
67	1	0	2.955179	2.978927	0.555366
68	1	0	3.355735	0.594203	1.352336
69	1	0	4.274196	-0.34998	3.4768

70	1	0	3.679317	1.289826	3.755738
71	1	0	2.709537	-0.09378	4.2726
72	1	0	3.284416	-1.84404	1.778174
73	1	0	1.900547	-1.36258	0.783461
74	1	0	1.68004	-1.70028	2.504815
75	1	0	-0.11918	0.085155	2.729603
76	1	0	-4.86199	0.181654	1.724038
77	1	0	-3.13743	0.113809	2.093006
78	1	0	-3.98397	1.659816	2.118204
79	1	0	0.769663	-0.27119	-2.91417
80	1	0	0.860683	-1.05231	-1.31149
81	1	0	-0.40324	-1.53417	-2.47724

Cartesian coordinates of optimized compound **1D**

Center Number	Atomic Number	Atomic Type	Coordinates (Angstroms)		
			X	Y	Z
1	6	0	-9.86133	-1.21338	-1.55476
2	6	0	-9.62281	-2.58469	-2.17813
3	6	0	-8.56501	-3.3631	-1.39016
4	6	0	-7.25383	-2.56936	-1.26334
5	6	0	-7.45226	-1.1671	-0.60772
6	6	0	-8.55238	-0.41952	-1.40756
7	6	0	-6.15042	-3.37175	-0.56179
8	6	0	-4.88717	-2.56458	-0.45101
9	6	0	-4.86554	-1.21905	-0.53815
10	6	0	-6.10638	-0.41545	-0.6354
11	6	0	-3.58879	-0.41975	-0.63665
12	6	0	-3.6302	0.910477	0.16416
13	6	0	-4.76751	1.74821	-0.45288
14	6	0	-6.03385	0.93502	-0.62764
15	6	0	-2.24271	-1.03905	-0.26139
16	6	0	-1.29686	0.177265	-0.31389
17	6	0	-2.18069	1.454641	-0.08134
18	8	0	-0.29822	0.008928	0.681406
19	6	0	0.633326	1.085686	0.73985
20	6	0	-0.05747	2.476593	0.785082
21	6	0	-1.57352	2.385936	0.987692
22	6	0	1.50097	1.044241	2.035695
23	6	0	1.813268	2.559461	2.183177
24	8	0	0.535926	3.167489	1.905661
25	6	0	-7.87491	-1.28609	0.882125
26	8	0	-10.8845	-3.2645	-2.20904
27	6	0	-2.20467	3.785435	0.996894

28	6	0	2.907674	3.143229	1.285125
29	6	0	2.729817	0.112509	1.968632
30	6	0	3.615159	0.240379	3.222056
31	6	0	2.33844	-1.36015	1.75491
32	8	0	0.728276	0.631624	3.163776
33	1	0	-6.89246	-2.37325	-2.28533
34	1	0	-3.5091	-0.11905	-1.69492
35	6	0	-3.91874	0.660806	1.660476
36	1	0	-1.72338	1.932665	1.974145
37	1	0	-2.18205	1.991952	-1.03753
38	1	0	0.156092	3.04067	-0.13418
39	1	0	1.298361	1.029557	-0.13033
40	8	0	-0.70251	0.365015	-1.60256
41	6	0	0.124098	-0.68611	-2.09732
42	1	0	-10.3446	-1.35743	-0.58077
43	1	0	-10.5702	-0.65222	-2.17404
44	1	0	-9.26181	-2.44408	-3.21142
45	1	0	-8.35701	-4.31983	-1.89158
46	1	0	-8.97467	-3.61008	-0.40256
47	1	0	-8.78108	0.539674	-0.93084
48	1	0	-8.15742	-0.18403	-2.40517
49	1	0	-6.49208	-3.71011	0.429103
50	1	0	-5.94734	-4.2904	-1.12897
51	1	0	-3.95096	-3.10898	-0.34921
52	1	0	-4.99744	2.625742	0.165125
53	1	0	-4.44539	2.147377	-1.42767
54	1	0	-6.94953	1.514923	-0.70581
55	1	0	-1.92156	-1.8376	-0.93357
56	1	0	-2.25053	-1.44589	0.753688
57	1	0	2.034704	2.787276	3.229762
58	1	0	-8.08101	-0.28946	1.285832
59	1	0	-7.08001	-1.72666	1.49096
60	1	0	-8.77428	-1.89418	1.015117
61	1	0	-10.759	-4.13911	-2.60053
62	1	0	-3.2719	3.745326	1.233298
63	1	0	-1.71661	4.420174	1.741732
64	1	0	-2.09738	4.266981	0.017025
65	1	0	3.894716	2.76138	1.562111
66	1	0	2.743346	2.919386	0.226324
67	1	0	2.918274	4.231053	1.401124
68	1	0	3.31686	0.435723	1.098021
69	1	0	4.492528	-0.40893	3.129713
70	1	0	3.9772	1.261384	3.380843

71	1	0	3.059964	-0.06155	4.114812
72	1	0	3.240016	-1.97833	1.679492
73	1	0	1.747689	-1.50098	0.846589
74	1	0	1.740426	-1.72464	2.595075
75	1	0	0.024439	1.280568	3.302508
76	1	0	-4.86773	0.135277	1.790201
77	1	0	-3.13871	0.063007	2.14043
78	1	0	-3.99231	1.607339	2.205712
79	1	0	0.808631	-1.06159	-1.32878
80	1	0	-0.46916	-1.52311	-2.48656
81	1	0	0.701908	-0.25704	-2.91939

Cartesian coordinates of optimized compound **1E**

Center Number	Atomic Number	Atomic Type	Coordinates (Angstroms)		
			X	Y	Z
1	6	0	-9.81943	-1.35134	-1.54966
2	6	0	-9.55915	-2.7259	-2.17148
3	6	0	-8.49239	-3.48214	-1.38577
4	6	0	-7.19676	-2.66462	-1.25979
5	6	0	-7.41358	-1.2626	-0.60893
6	6	0	-8.52374	-0.53283	-1.41077
7	6	0	-6.08282	-3.44813	-0.55369
8	6	0	-4.83147	-2.62289	-0.44303
9	6	0	-4.82751	-1.27758	-0.536
10	6	0	-6.07902	-0.49141	-0.63953
11	6	0	-3.56151	-0.46134	-0.63535
12	6	0	-3.62326	0.872903	0.157525
13	6	0	-4.77028	1.69143	-0.46723
14	6	0	-6.02522	0.860005	-0.6391
15	6	0	-2.20821	-1.05985	-0.25243
16	6	0	-1.27892	0.168885	-0.30984
17	6	0	-2.18077	1.435456	-0.08765
18	8	0	-0.28101	0.020424	0.689501
19	6	0	0.635616	1.109987	0.743757
20	6	0	-0.07402	2.491668	0.777637
21	6	0	-1.58923	2.381713	0.977063
22	6	0	1.499648	1.088915	2.042514
23	6	0	1.791439	2.609099	2.180452
24	8	0	0.507084	3.198366	1.894838
25	6	0	-7.83434	-1.38184	0.88149
26	8	0	-10.7364	-3.54377	-2.18937
27	6	0	-2.23941	3.772476	0.975905
28	6	0	2.880769	3.201194	1.281677

29	6	0	2.740949	0.17316	1.985709
30	6	0	3.620305	0.321193	3.241116
31	6	0	2.369857	-1.30597	1.78065
32	8	0	0.729013	0.673906	3.171111
33	1	0	-6.83566	-2.46674	-2.28155
34	1	0	-3.48257	-0.16567	-1.69511
35	6	0	-3.91205	0.628183	1.654595
36	1	0	-1.73553	1.932567	1.965943
37	1	0	-2.18692	1.966723	-1.0472
38	1	0	0.13413	3.052256	-0.145
39	1	0	1.304238	1.057201	-0.12391
40	8	0	-0.68327	0.356932	-1.59775
41	6	0	0.158006	-0.68661	-2.08406
42	1	0	-10.2957	-1.49898	-0.57265
43	1	0	-10.5363	-0.79124	-2.16642
44	1	0	-9.19976	-2.58616	-3.20544
45	1	0	-8.2817	-4.43353	-1.88903
46	1	0	-8.89867	-3.73512	-0.39838
47	1	0	-8.76571	0.425947	-0.93964
48	1	0	-8.13461	-0.29874	-2.41097
49	1	0	-6.4211	-3.78868	0.43759
50	1	0	-5.8662	-4.36545	-1.11754
51	1	0	-3.88803	-3.15381	-0.33653
52	1	0	-5.01315	2.569665	0.144838
53	1	0	-4.45122	2.088681	-1.4438
54	1	0	-6.94861	1.427133	-0.72096
55	1	0	-1.87425	-1.85816	-0.91861
56	1	0	-2.21346	-1.46045	0.765139
57	1	0	2.006627	2.846972	3.226109
58	1	0	-8.05996	-0.38756	1.280805
59	1	0	-7.03063	-1.80369	1.491825
60	1	0	-8.72045	-2.00841	1.017798
61	1	0	-11.4291	-3.08318	-2.68136
62	1	0	-3.30665	3.719139	1.209671
63	1	0	-1.76207	4.41837	1.718105
64	1	0	-2.13596	4.249428	-0.00663
65	1	0	3.87193	2.834148	1.563895
66	1	0	2.722375	2.968196	0.223947
67	1	0	2.876819	4.289814	1.390461
68	1	0	3.326575	0.498265	1.114843
69	1	0	4.506679	-0.31677	3.155876
70	1	0	3.967954	1.348031	3.394359
71	1	0	3.066282	0.017587	4.134031

72	1	0	3.279797	-1.91264	1.712684
73	1	0	1.784371	-1.46079	0.871196
74	1	0	1.77362	-1.67267	2.621113
75	1	0	0.016629	1.314759	3.303655
76	1	0	-4.85373	0.089901	1.785179
77	1	0	-3.12485	0.044411	2.140063
78	1	0	-4.00039	1.576835	2.193962
79	1	0	0.843088	-1.05072	-1.31057
80	1	0	-0.42371	-1.53198	-2.47255
81	1	0	0.734809	-0.25432	-2.90516

3. Spectral information of 1

3.1 HR-ESI-MS and UV (CH_3OH) spectrum of 1

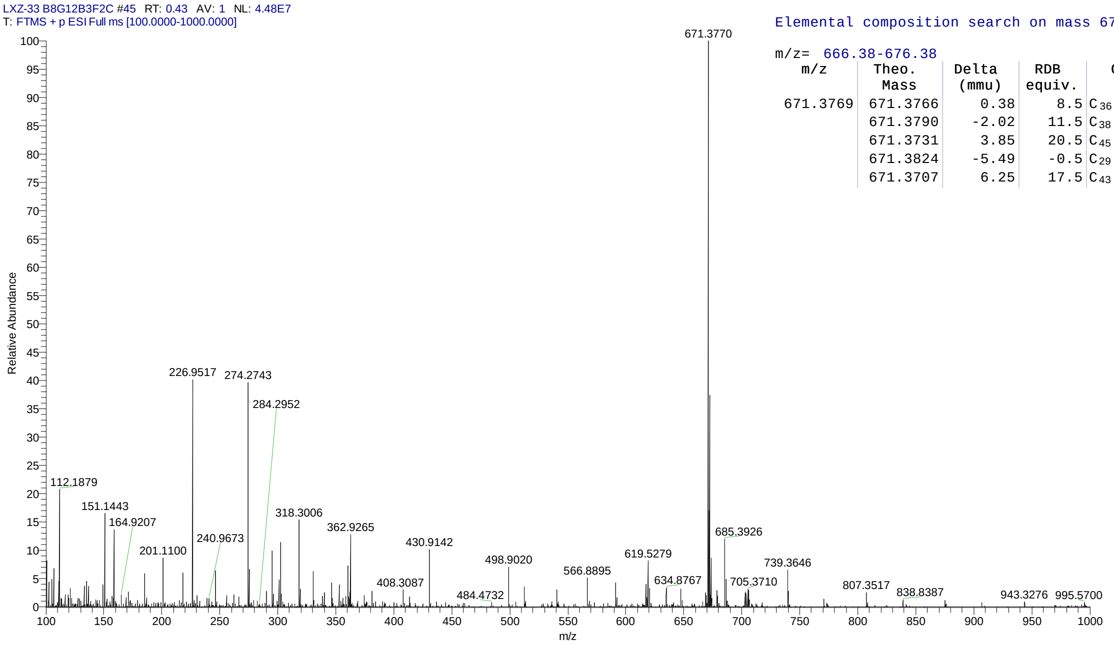

Elemental composition search on mass 671.38

m/z= 666.38-676.38

m/z	Theo. Mass	Delta (mmu)	RDB equiv.	Composition
671.3769	671.3766	0.38	8.5	$C_{36}H_{56}O_{10}Na$
	671.3790	-2.02	11.5	$C_{38}H_{55}O_{10}$
	671.3731	3.85	20.5	$C_{45}H_{51}O_5$
	671.3824	-5.49	-0.5	$C_{29}H_{60}O_{15}Na$
	671.3707	6.25	17.5	$C_{43}H_{52}O_5Na$

HR-ESI-MS of compound 1

Spectrum Peak Pick Report

2018-01-09 01:21:10

Data Set: B8G12B3F2C - RawData

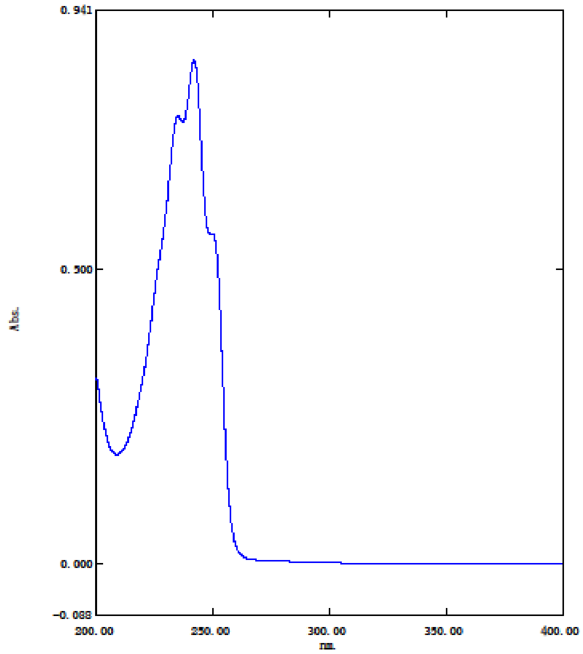

[Measurement Properties]
Wavelength Range (nm.): 200.00 to 400.00
Scan Speed: Medium
Sampling Interval: 0.2
Auto Sampling Interval: Disabled
Scan Mode: Auto

[Instrument Properties]
Instrument Type: UV-2600 Series
Measuring Mode: Absorbance
Slit Width: 2.0
Accumulation time: 0.1 sec.
Light Source Change Wavelength: 323.0 nm
Detector Unit: Direct
S/R Exchange: Normal
Stair Correction: OFF

[Attachment Properties]
Attachment: None

[Operation]
Threshold: 0.0010000
Points: 5
InterPolate: Disabled
Average: Disabled

[Sample Preparation Properties]
Weight:
Volume:
Dilution:
Path length:
Addition: Member:

No.	P/V	Wavelengh (nm)	Absorbance	description

页 1 / 1

UV spectrum of 1

3.2 1D and 2D NMR spectra of 1 in pyridine-d5

3.2.1 ^{1}H NMR spectrum of 1 in pyridine-d5

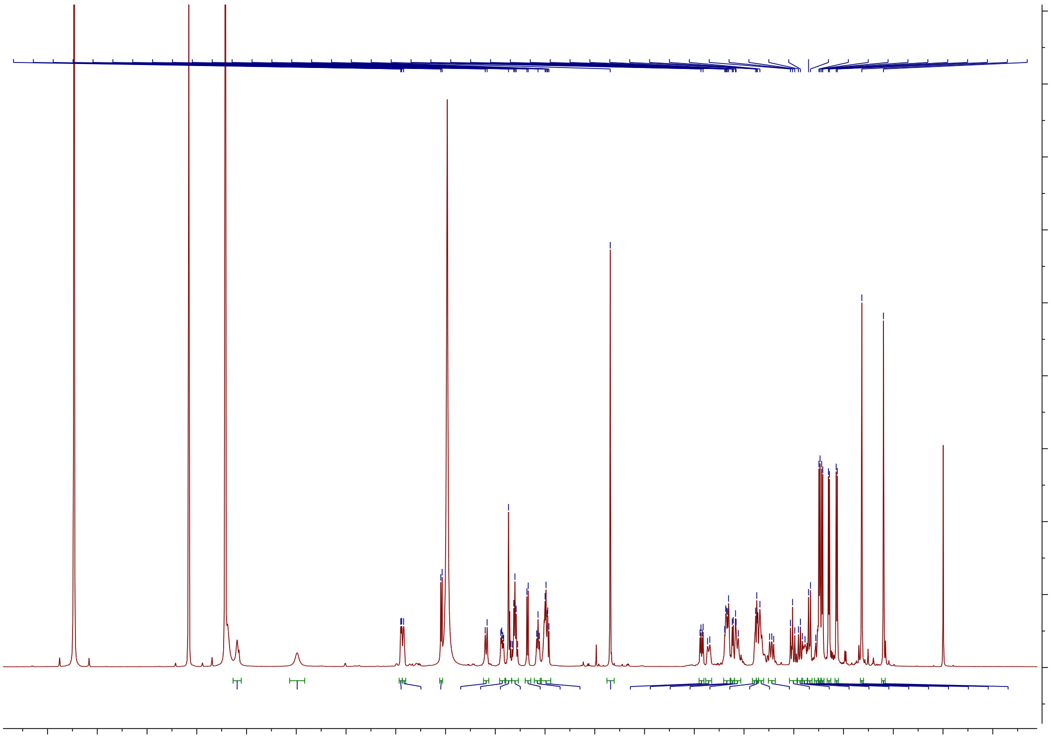

3.2.2 ^{13}C NMR spectrum of 1 in pyridine-d5

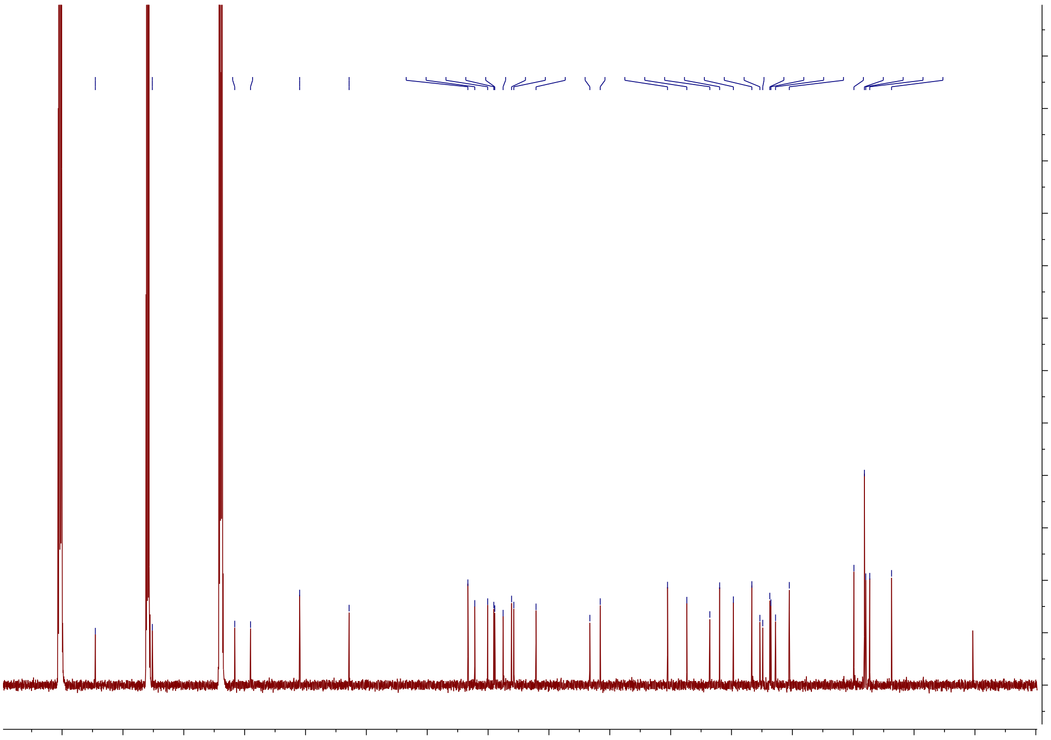

3.2.3 ^{1}H–^{1}H COSY spectrum of 1 in pyridine-d$_5$

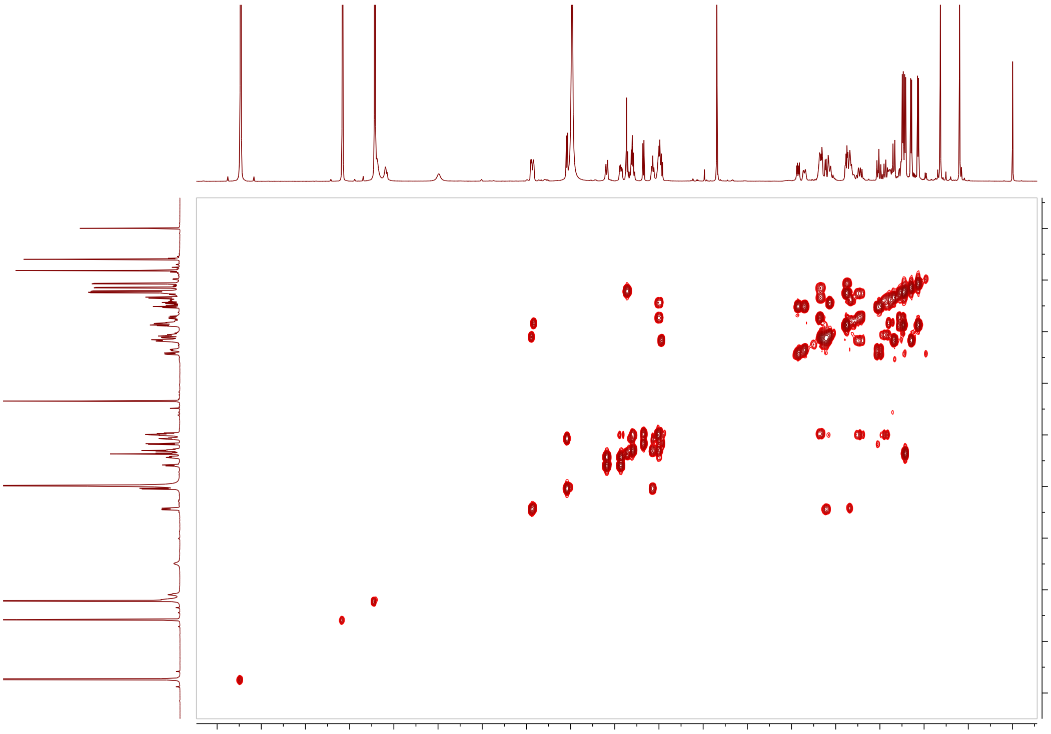

3.2.4 HSQC spectrum of 1 in pyridine-d$_5$

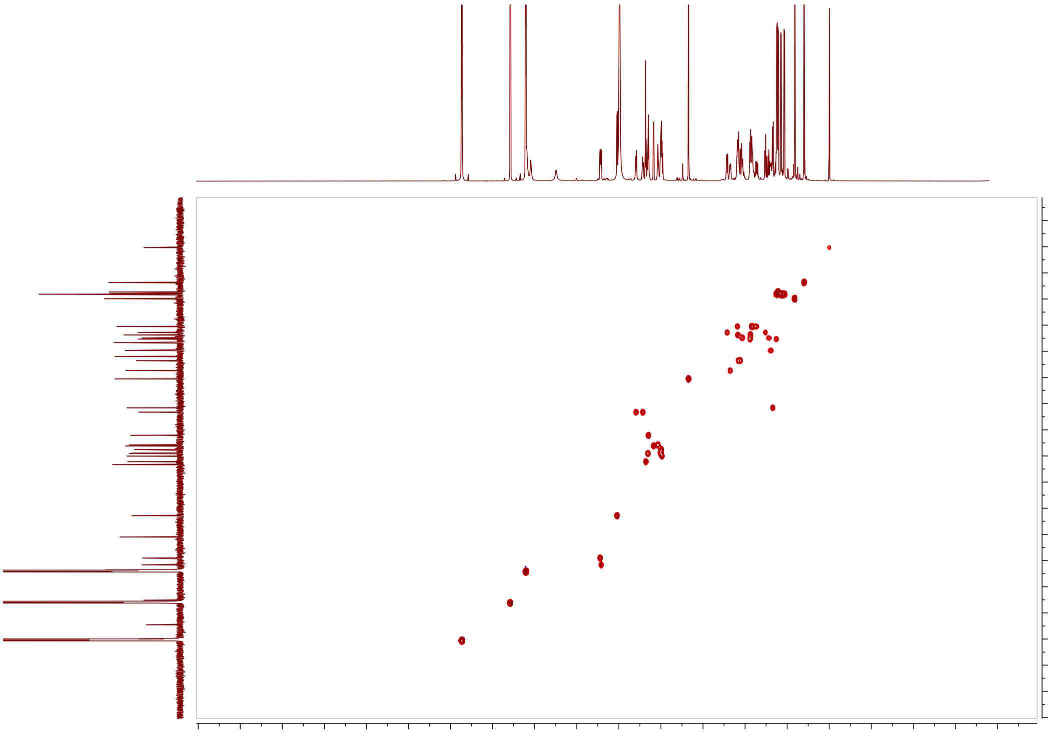

3.2.5 HMBC spectrum of 1 in pyridine-d_5

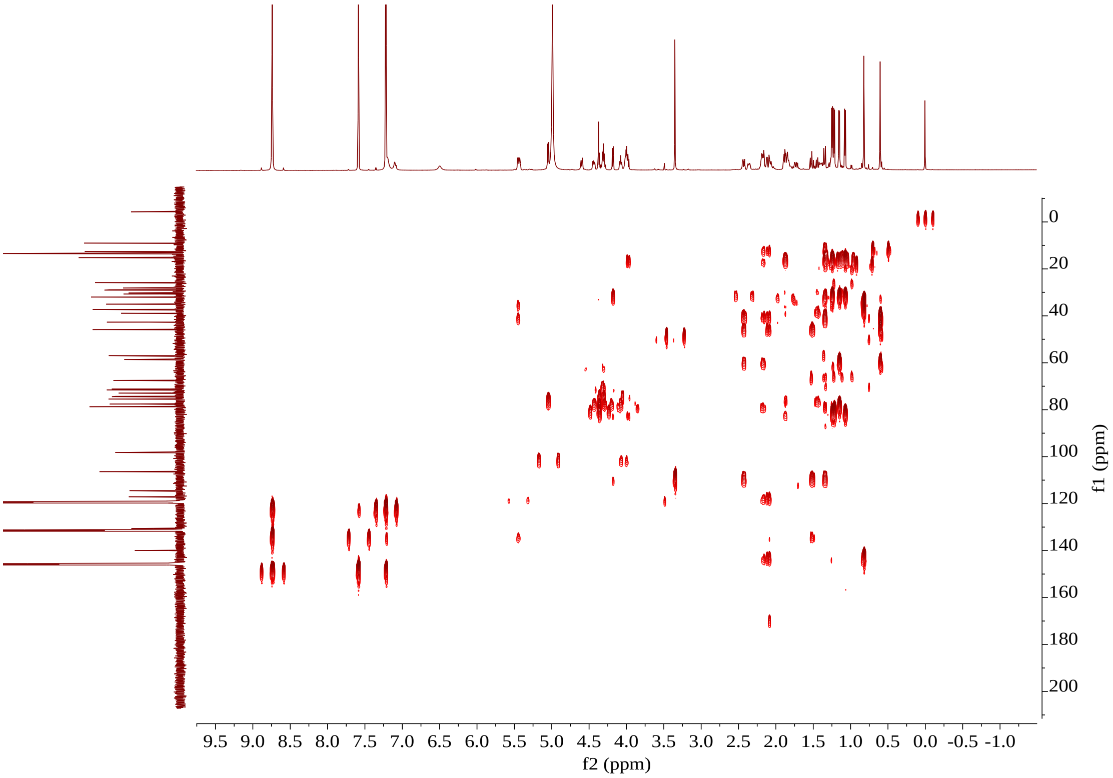

3.2.6 NOESY spectrum of 1 in pyridine-d_5

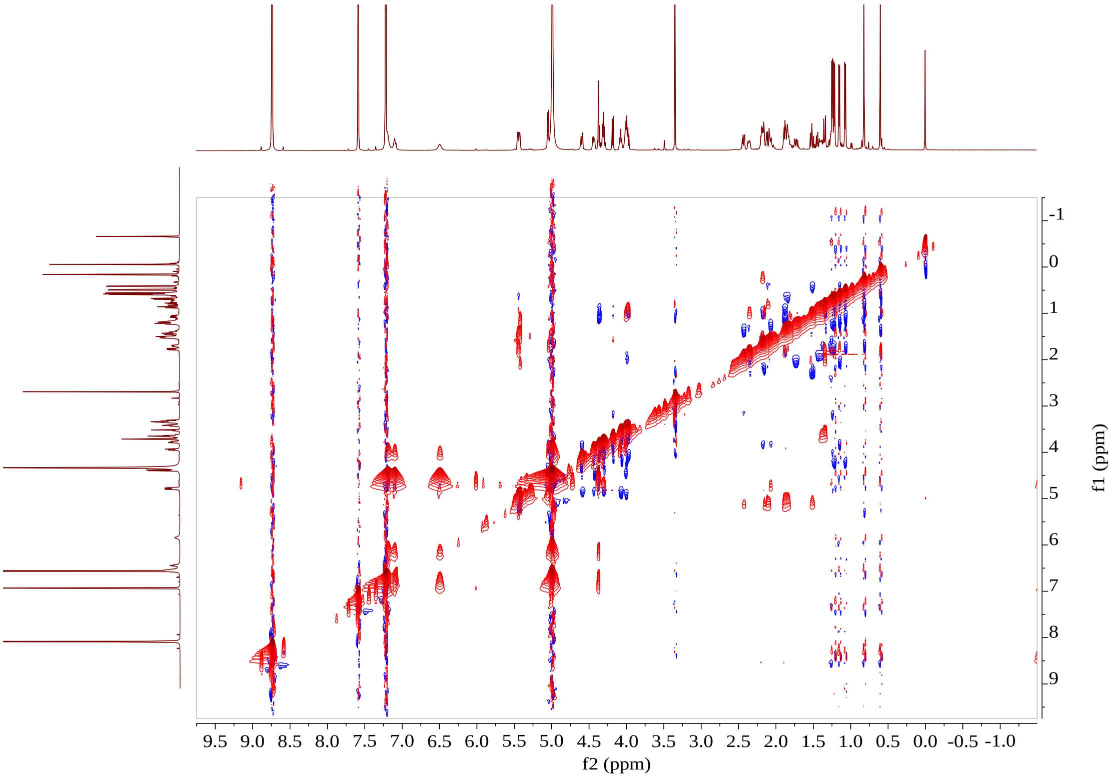

4. Spectral information of 2

4.1 HR-ESI-MS and UV (CH_3OH) spectrum of 2

HR-ESI-MS and UV (CH3OH) spectrum of 2

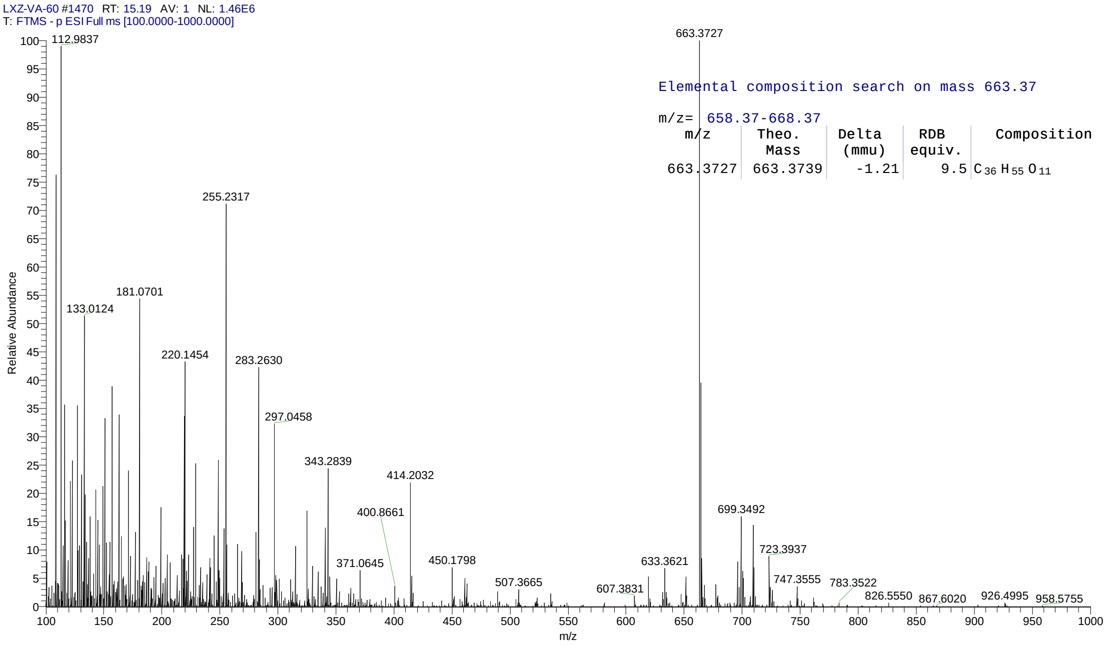

HR-ESI-MS of compound 2

Spectrum Peak Pick Report

2019-11-07 19:58:30

Data Set: VA-60 0.0233 mg ml - RawData

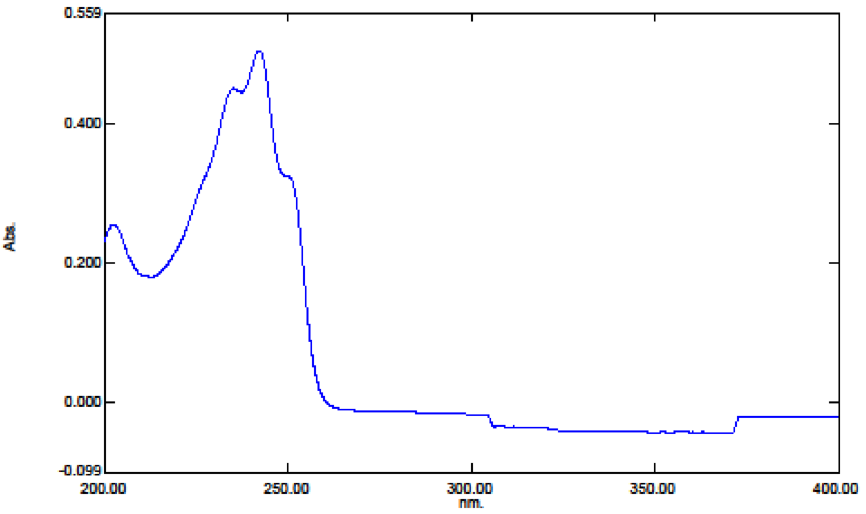

No.	P/V	Wavelength	Abs.	Description
1	P	373.00	-0.020	
2	P	242.00	0.504	
3	P	235.00	0.450	
4	P	202.00	0.254	
5	V	367.00	-0.044	
6	V	237.50	0.444	
7	V	212.50	0.180	

[Measurement Properties]
Wavelength Range (nm.): 200.00 to 400.00
Scan Speed: Medium
Sampling Interval: 0.5
Auto Sampling Interval: Disabled
Scan Mode: Auto

[Instrument Properties]
Instrument Type: UV-2600 Series
Measuring Mode: Absorbance
Slit Width: 2.0
Accumulation time: 0.1 sec.
Light Source Change Wavelength: 323.0 nm
Detector Unit: Direct
S/R Exchange: Normal
Stair Correction: OFF

[Attachment Properties]
Attachment: None

[Operation]
Threshold: 0.0010000
Points: 4
InterPolate: Disabled
Average: Disabled

[Sample Preparation Properties]
Weight:
......

Page 1 / 1

UV spectrum of 2

4.2 1D and 2D NMR spectra of 2 in pyridine-d_5

4.2.1 ^{1}H NMR spectrum of 2 in pyridine-d_5

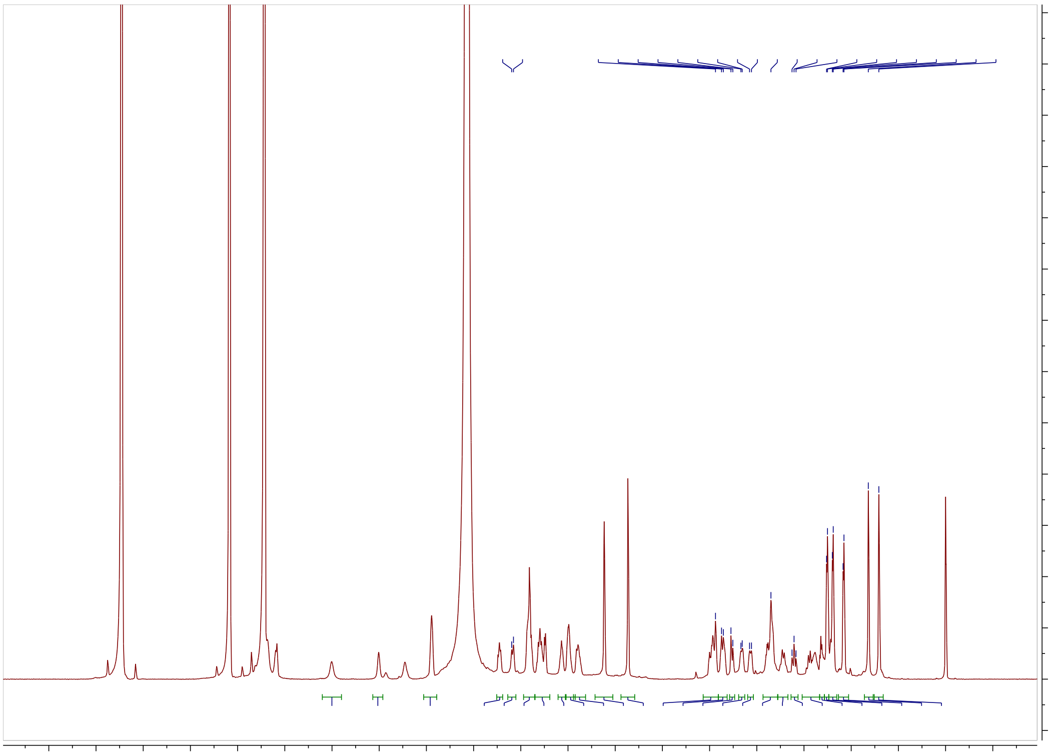

4.2.2 ^{13}C NMR spectrum of 2 in pyridine-d_5

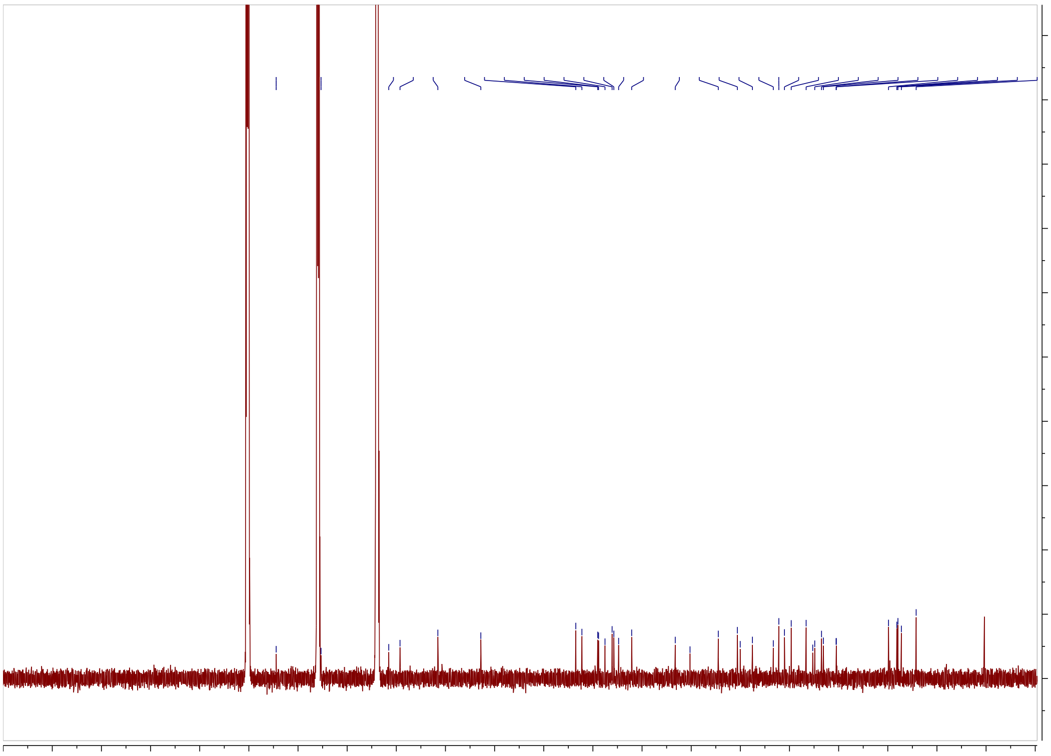

4.2.3 DEPT-135 spectrum of 2 in pyridine-d$_5$

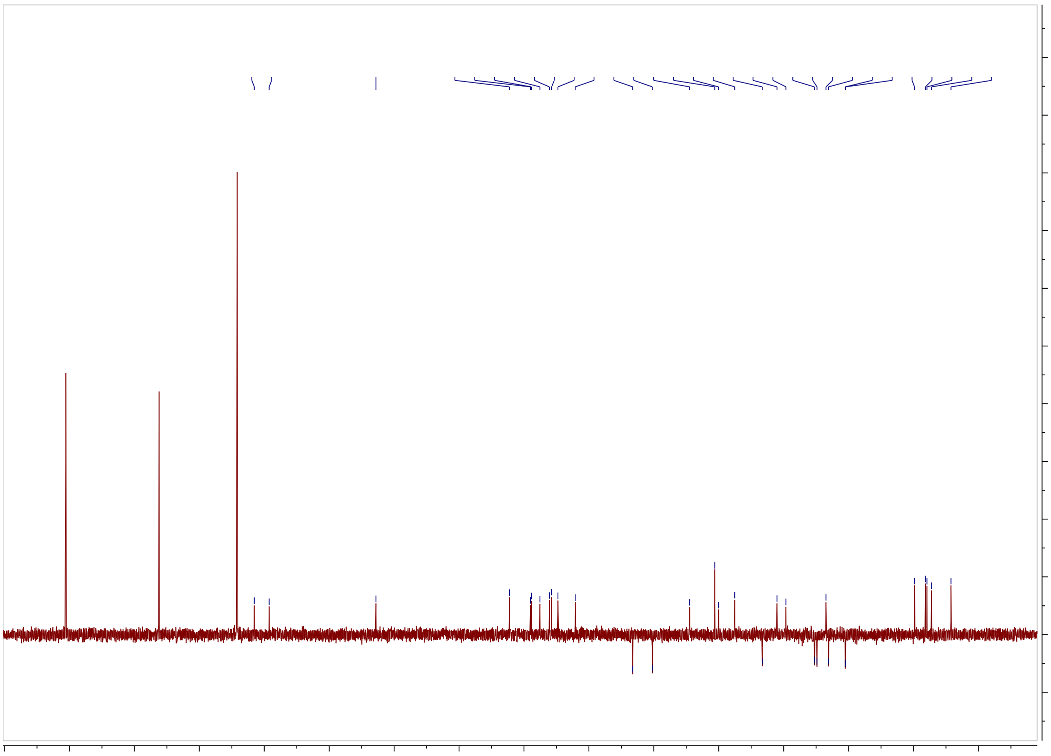

4.2.4 ^{1}H–^{1}H COSY spectrum of 2 in pyridine-d$_5$

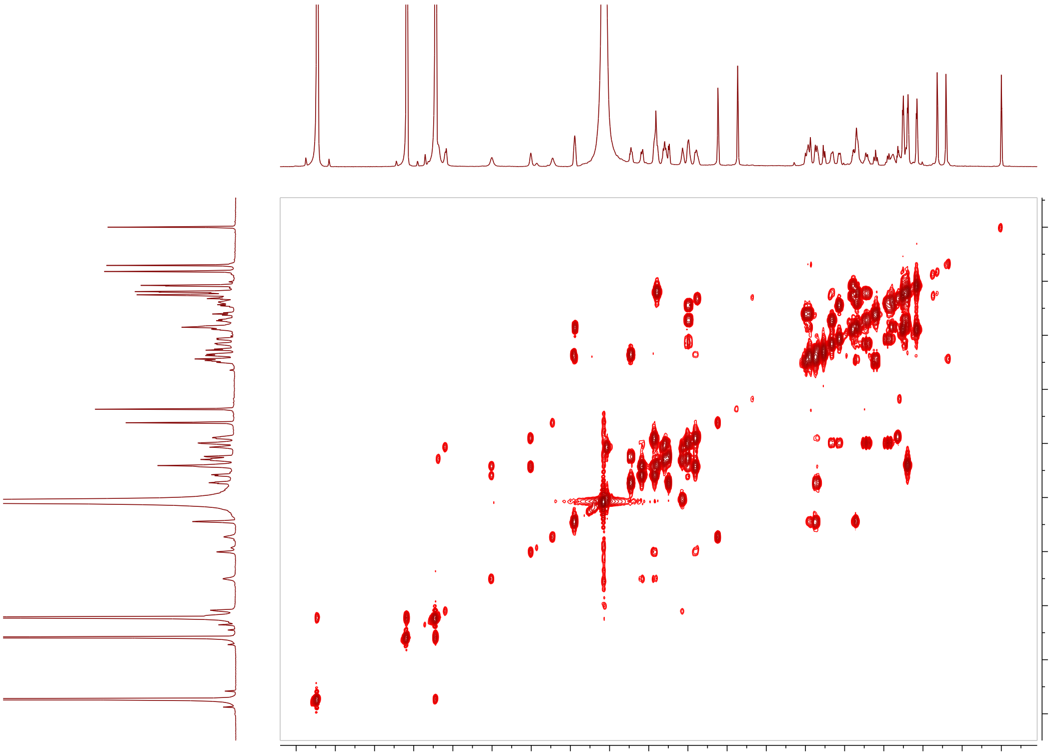

4.2.5 HSQC spectrum of 2 in pyridine-d_5

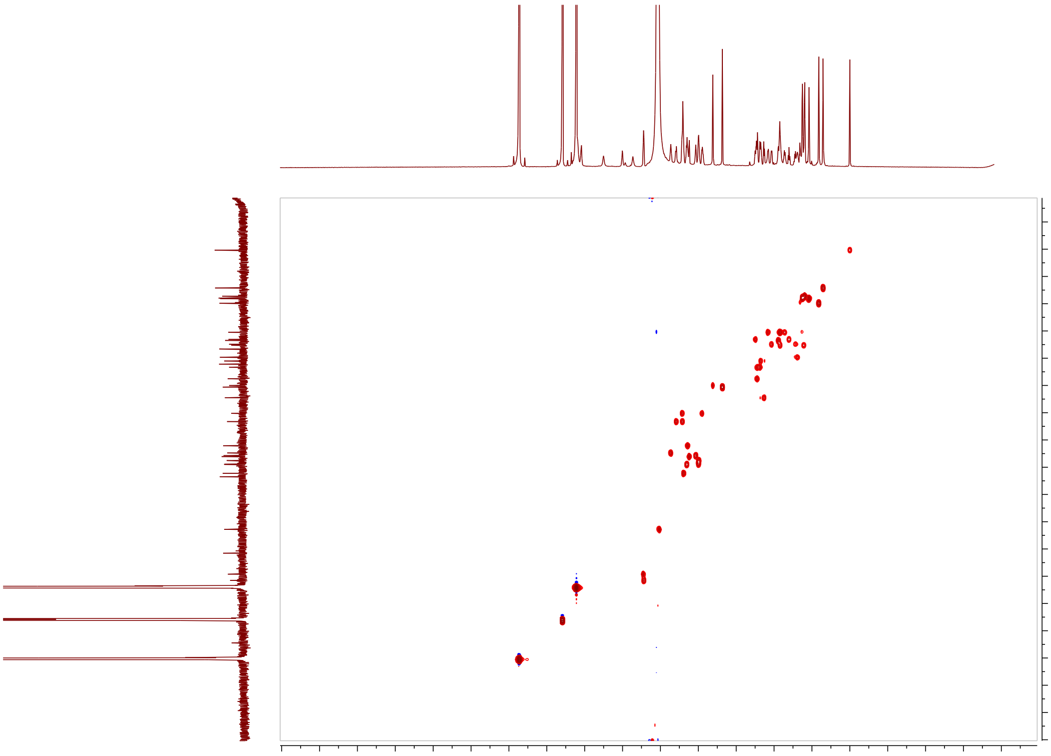

4.2.6 HMBC spectrum of 2 in pyridine-d_5

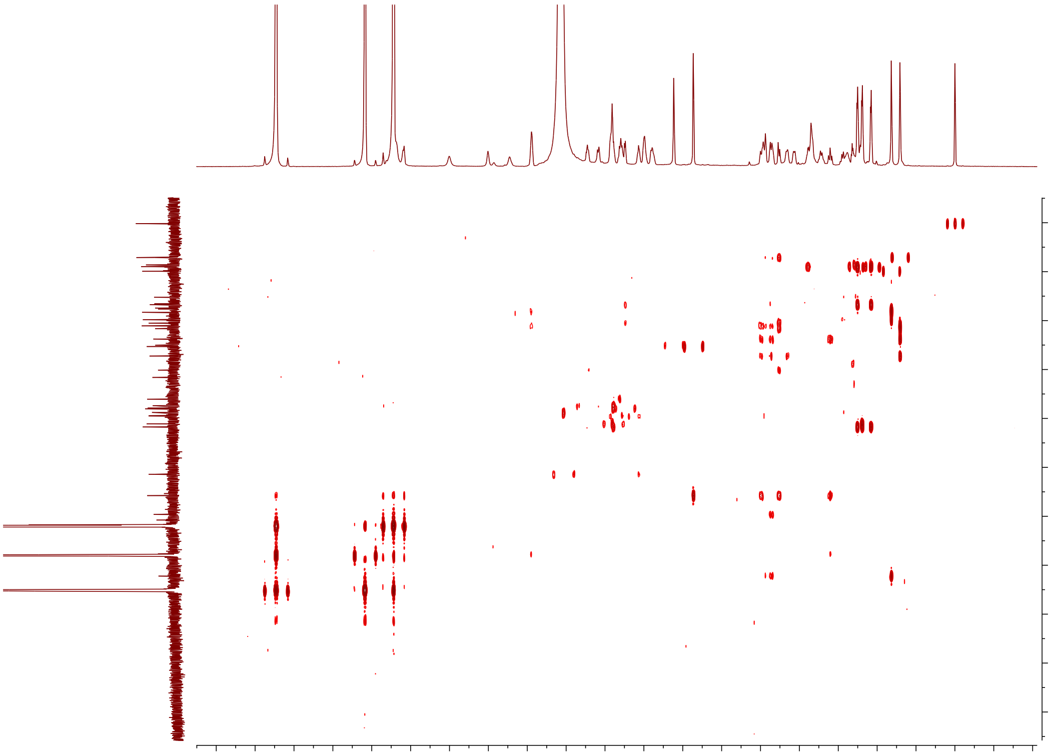

4.2.7 NOESY spectrum of 2 in pyridine-d_5

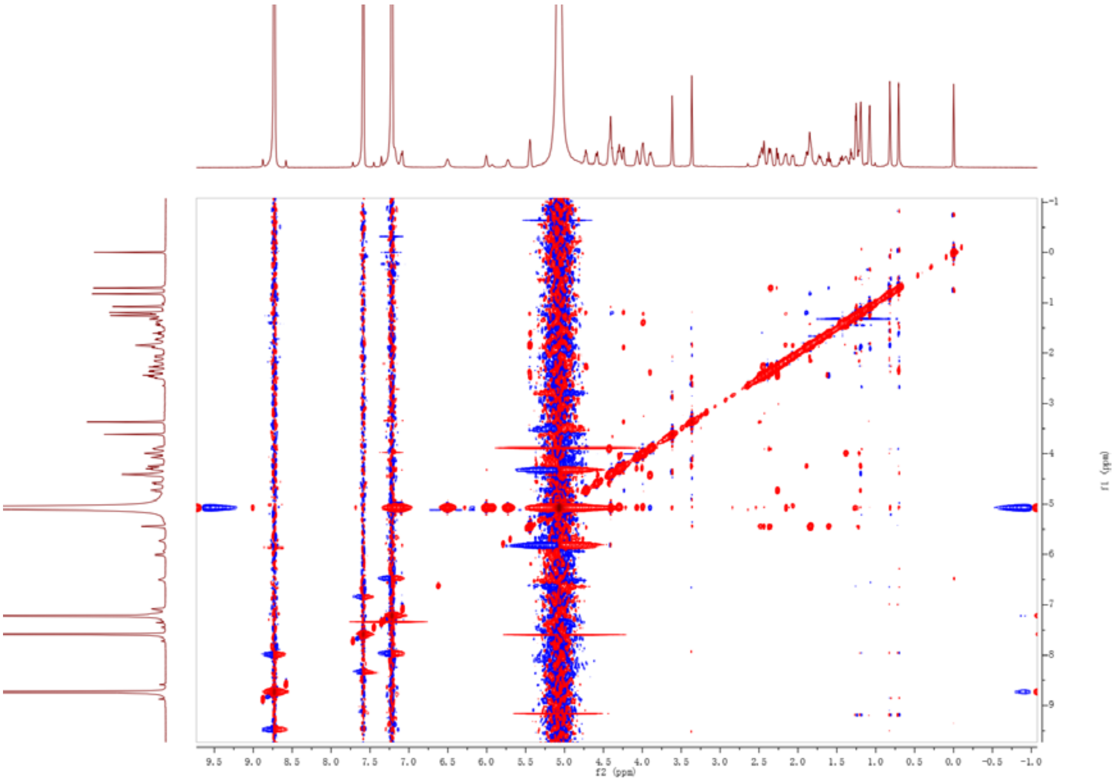

5. Spectral information of 3

5.1 HR-ESI-MS and UV (CH_3OH) spectrum of 3

HR-ESI-MS and UV (CH3OH) spectrum of 3

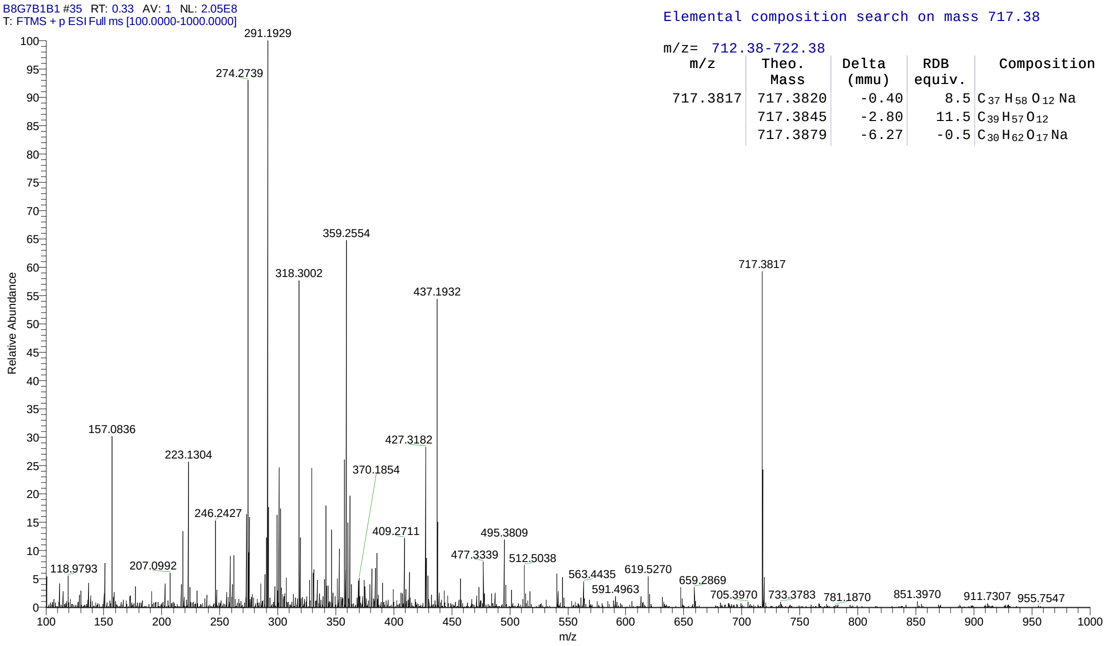

m/z	Theo. Mass	Delta (mmu)	RDB equiv.	Composition
717.3817	717.3820	-0.40	8.5	$C_{37}H_{58}O_{12}Na$
	717.3845	-2.80	11.5	$C_{39}H_{57}O_{12}$
	717.3879	-6.27	-0.5	$C_{30}H_{62}O_{17}Na$

HR-ESI-MS of compound 3

Spectrum Peak Pick Report

2019-05-08 21:24:34

Data Set: B8G7B1B1 0.0303 mg ml - RawData

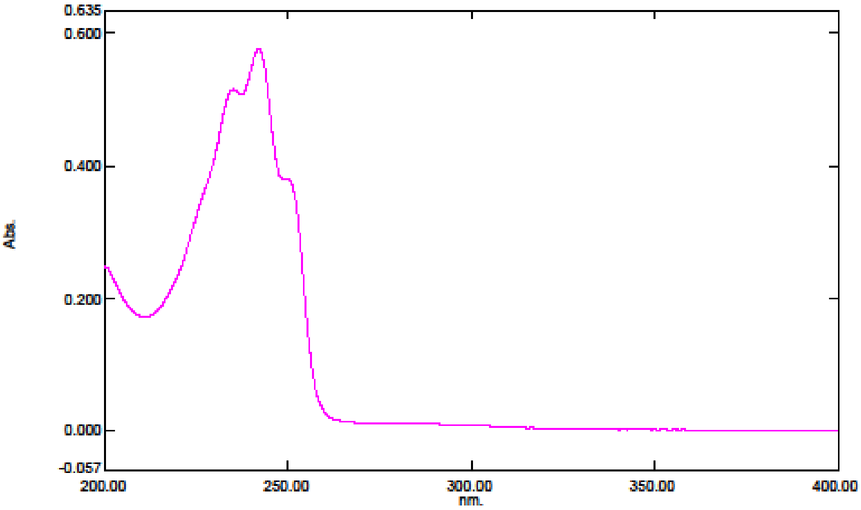

No.	P/V	Wavelength	Abs.	Description
1		329.80	0.004	
2		249.80	0.361	
3		242.00	0.578	
4		235.00	0.515	

[Measurement Properties]
Wavelength Range (nm.): 200.00 to 400.00
Scan Speed: Medium
Sampling Interval: 0.2
Auto Sampling Interval: Enabled
Scan Mode: Single

[Instrument Properties]
Instrument Type: UV-2600 Series
Measuring Mode: Absorbance
Slit Width: 2.0
Accumulation time: 0.1 sec.
Light Source Change Wavelength: 323.0 nm
Detector Unit: Direct
S/R Exchange: Normal
Stair Correction: OFF

[Attachment Properties]
Attachment: None

[Operation]
Threshold: 0.0010000
Points: 4
InterPolate: Disabled
Average: Disabled

[Sample Preparation Properties]
Weight:
......

Page 1 / 1

UV spectrum of 3

5.2 1D and 2D NMR spectra of 3 in pyridine-d_5

5.2.1 ^{1}H NMR spectrum of 3 in pyridine-d_5

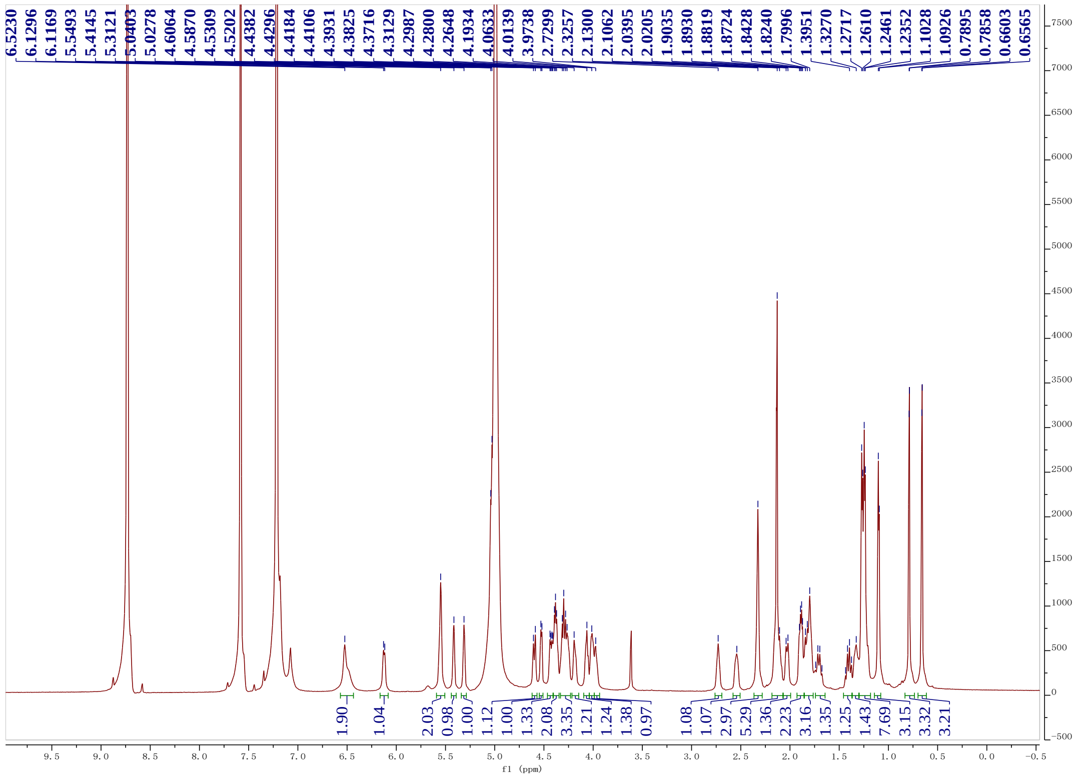

5.2.2 ^{13}C NMR spectrum of 3 in pyridine-d_5

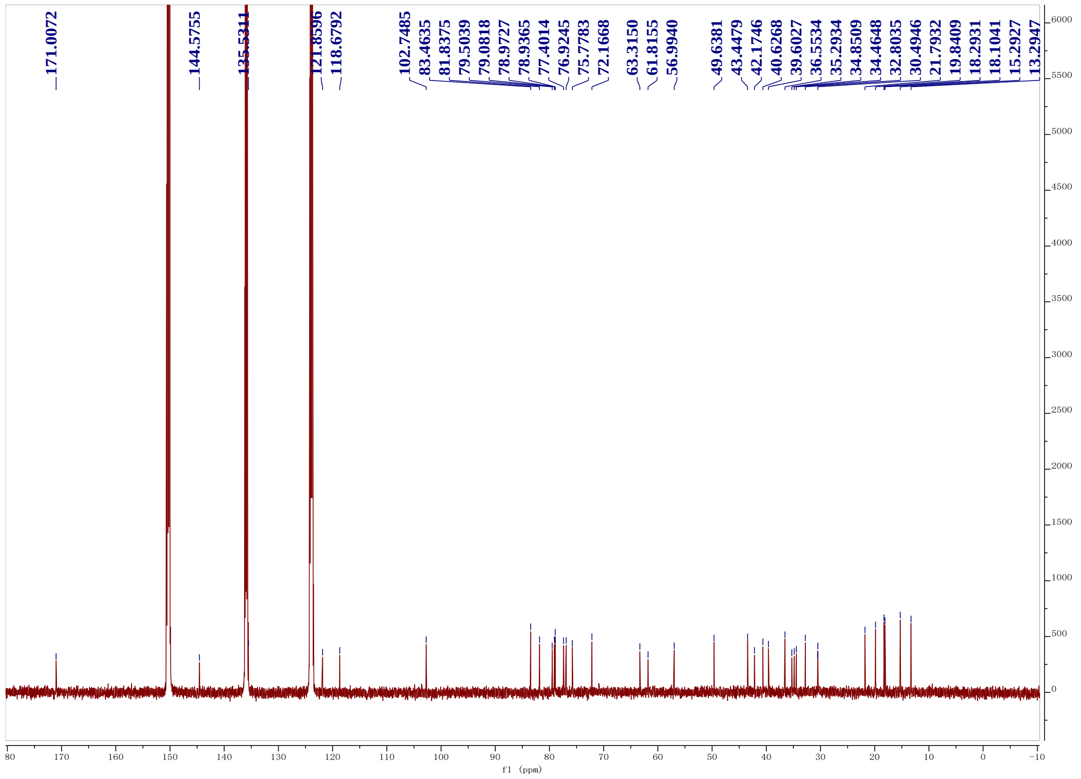

5.2.3 DEPT-135 spectrum of 3 in pyridine-d_5

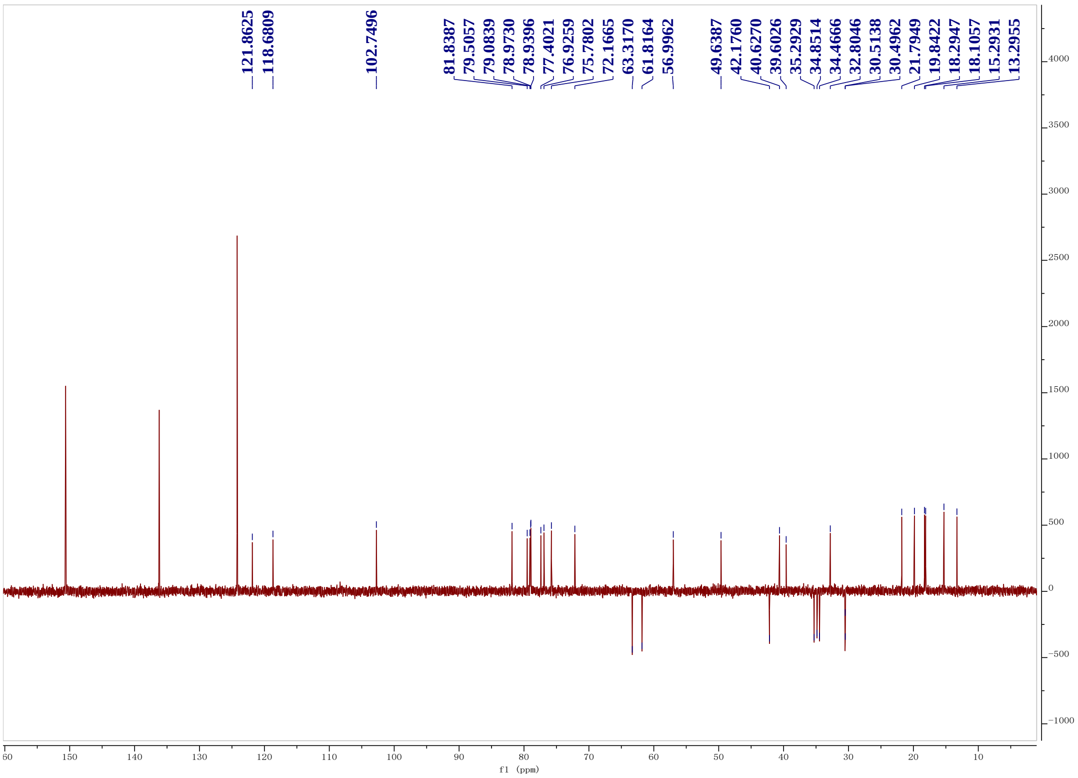

5.2.3 ^1H–^1H COSY spectrum of 3 in pyridine-d_5

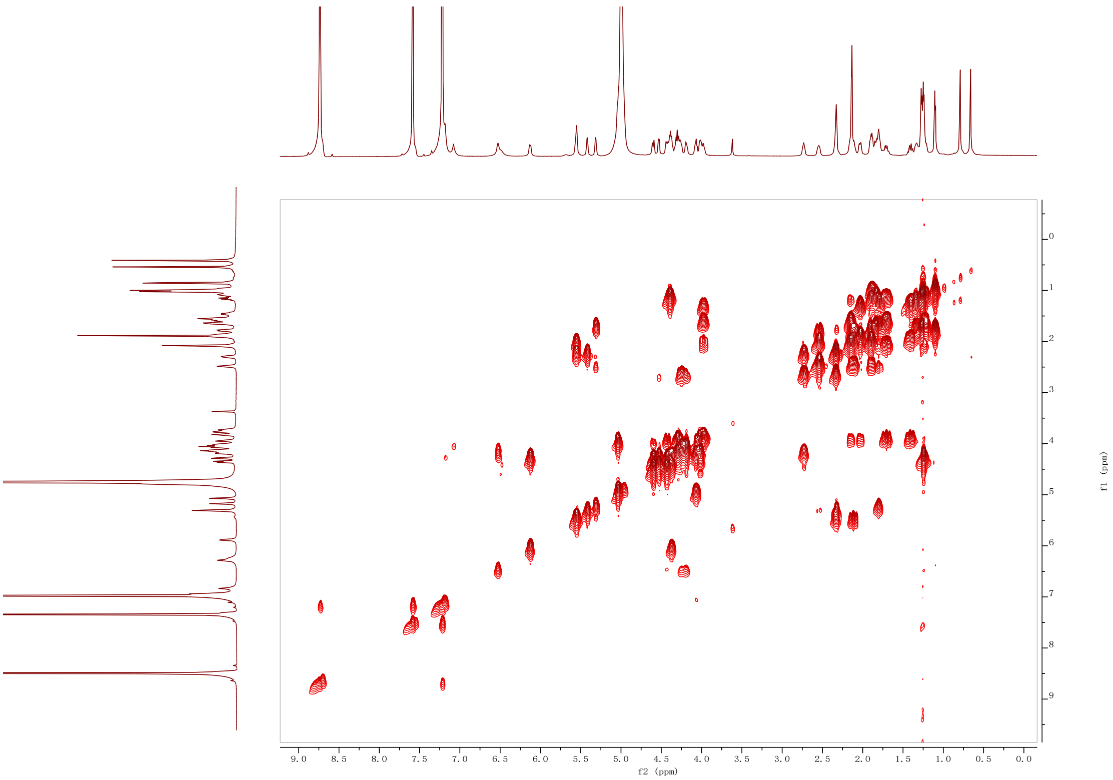

5.2.4 HSQC spectrum of 3 in pyridine-d_5

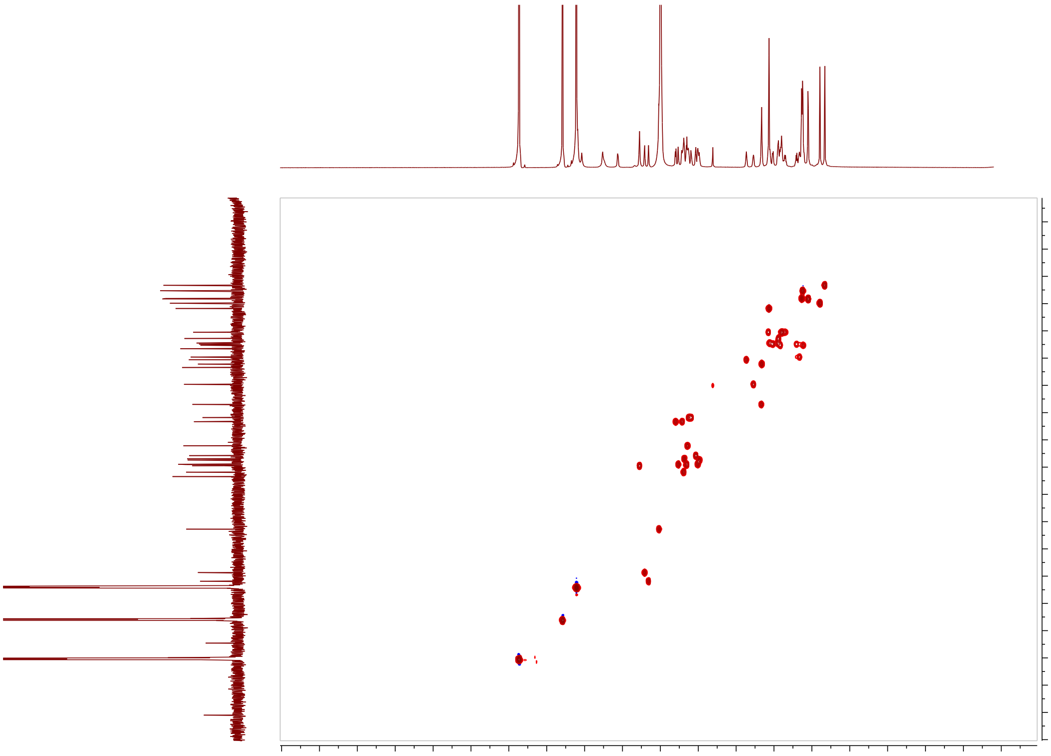

5.2.5 HMBC spectrum of 3 in pyridine-d_5

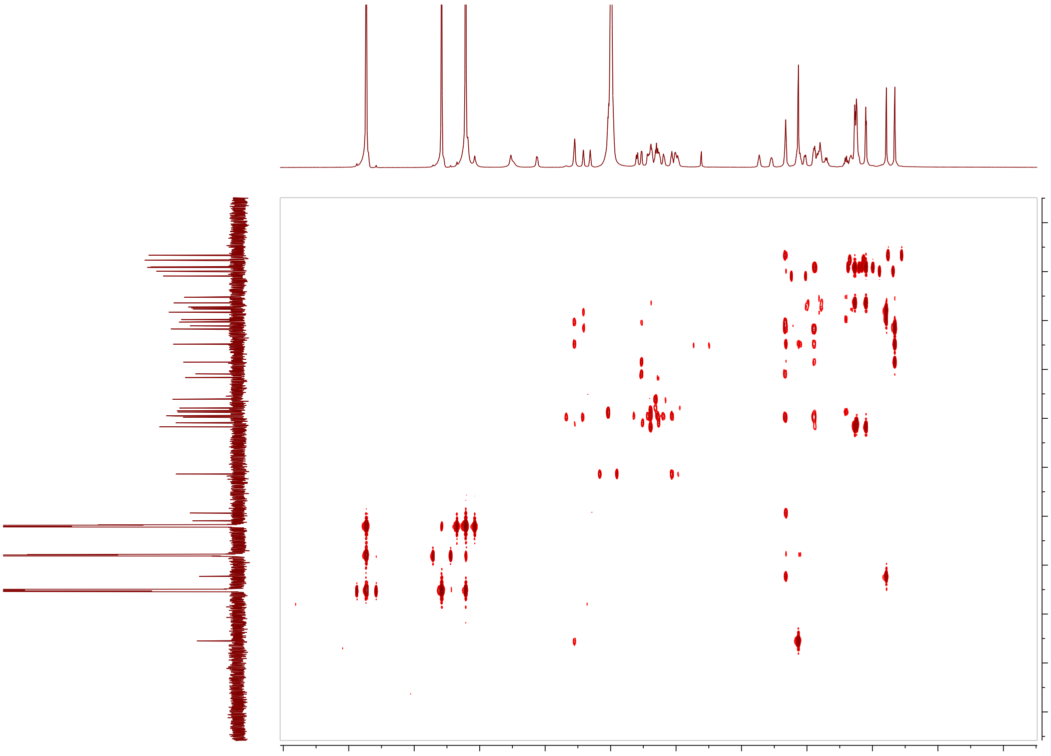

5.2.6 NOESY spectrum of 3 in pyridine-d_5

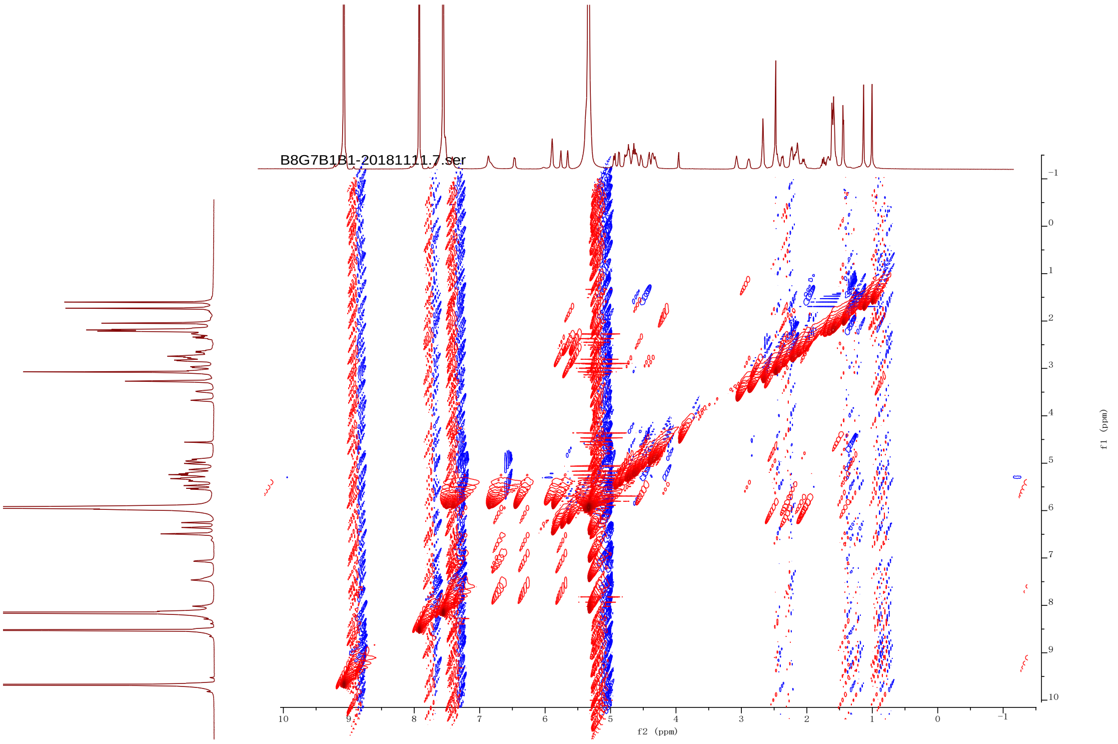

6. Spectral information of 4

6.1 HR-ESI-MS and UV (CH_3OH) spectrum of 4

HR-ESI-MS, IR and UV (CH3OH) spectrum of 4

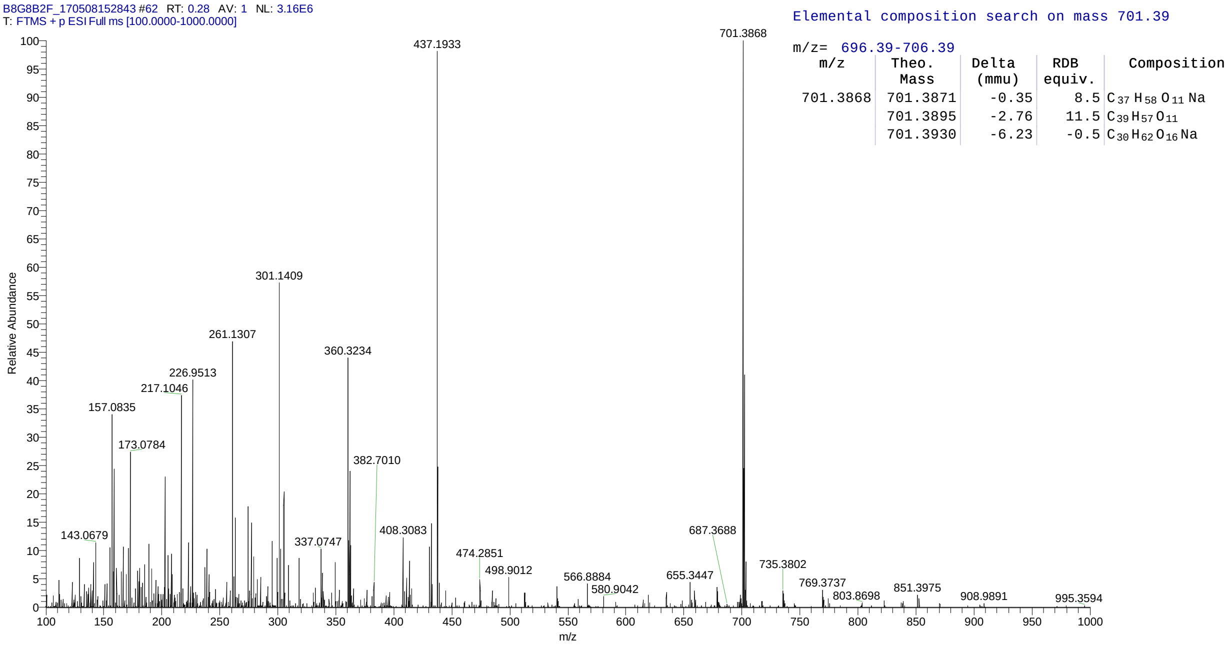

HR-ESI-MS of compound 4

Spectrum Peak Pick Report

2017-06-21 22:06:13

Data Set: st_220323 - RawData

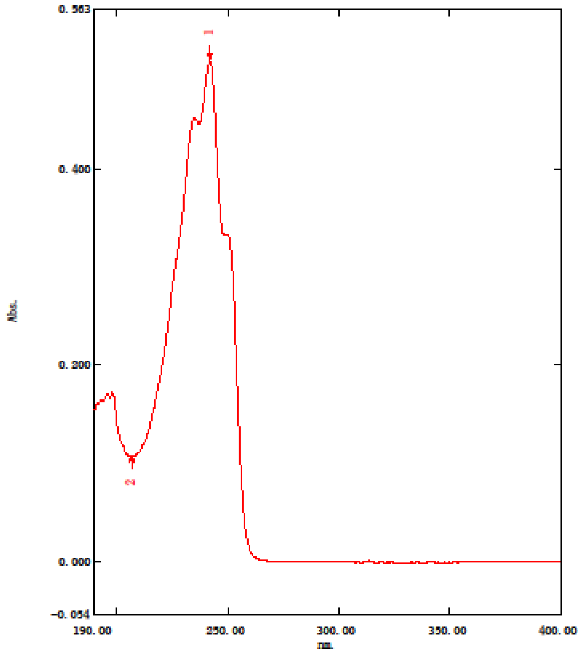

[Measurement Properties]
Wavelength Range (nm.): 190. 00 to 400. 00
Scan Speed: Medium
Sampling Interval: 1. 0
Auto Sampling Interval: Disabled
Scan Mode: Auto

[Instrument Properties]
Instrument Type: UV-2600 Series
Measuring Mode: Absorbance
Slit Width: 2. 0
Accumulation time: 0. 1 sec.
Light Source Change Wavelength: 323. 0 nm
Detector Unit: Direct
S/R Exchange: Normal
Stair Correction: OFF

[Attachment Properties]
Attachment: None

[Operation]
Threshold: 0. 0010000
Points: 5
InterPolate: Disabled
Average: Disabled

[Sample Preparation Properties]
Weight:
Volume:
Dilution:
Path length:
Addition: Member:

No.	P/V	Wavelength (nm)	Absorbance	description
1	↑	242. 00	0. 512	
2	↓	207. 00	0. 107	

页 1 / 1

UV spectrum of 4

6.2 1D and 2D NMR spectra of 4 in pyridine-d_5

6.2.1 ^{1}H NMR spectrum of 4 in pyridine-d_5

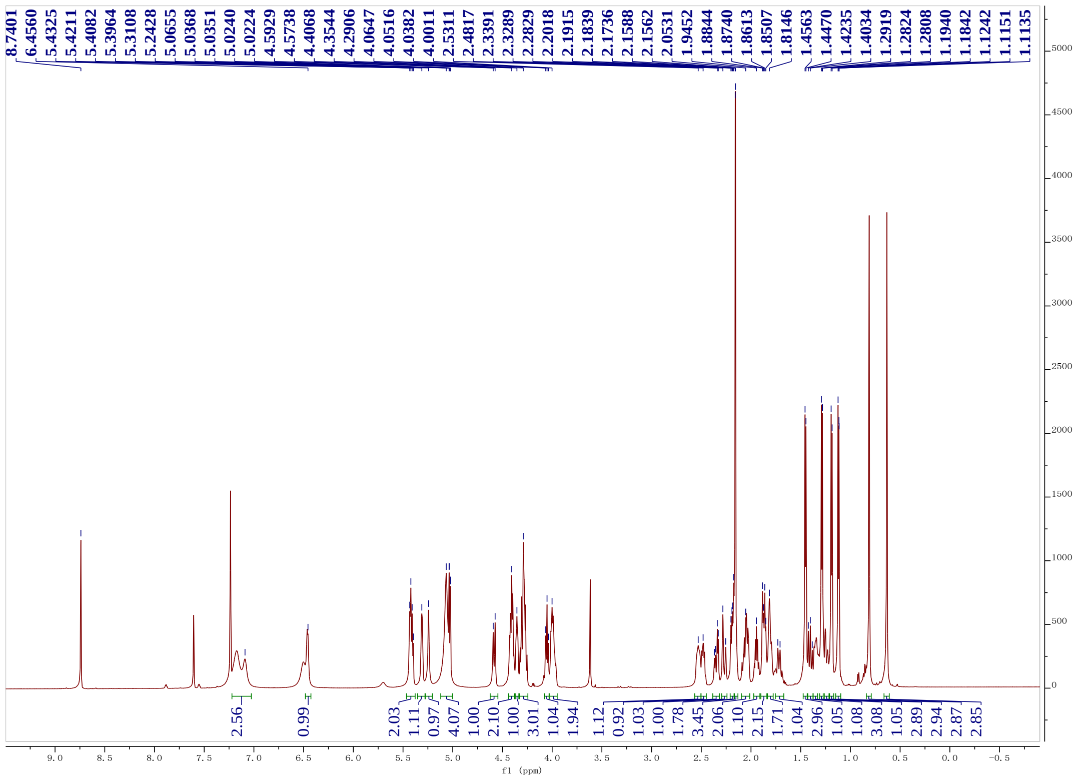

6.2.2 ^{13}C NMR spectrum of 4 in pyridine-d_5

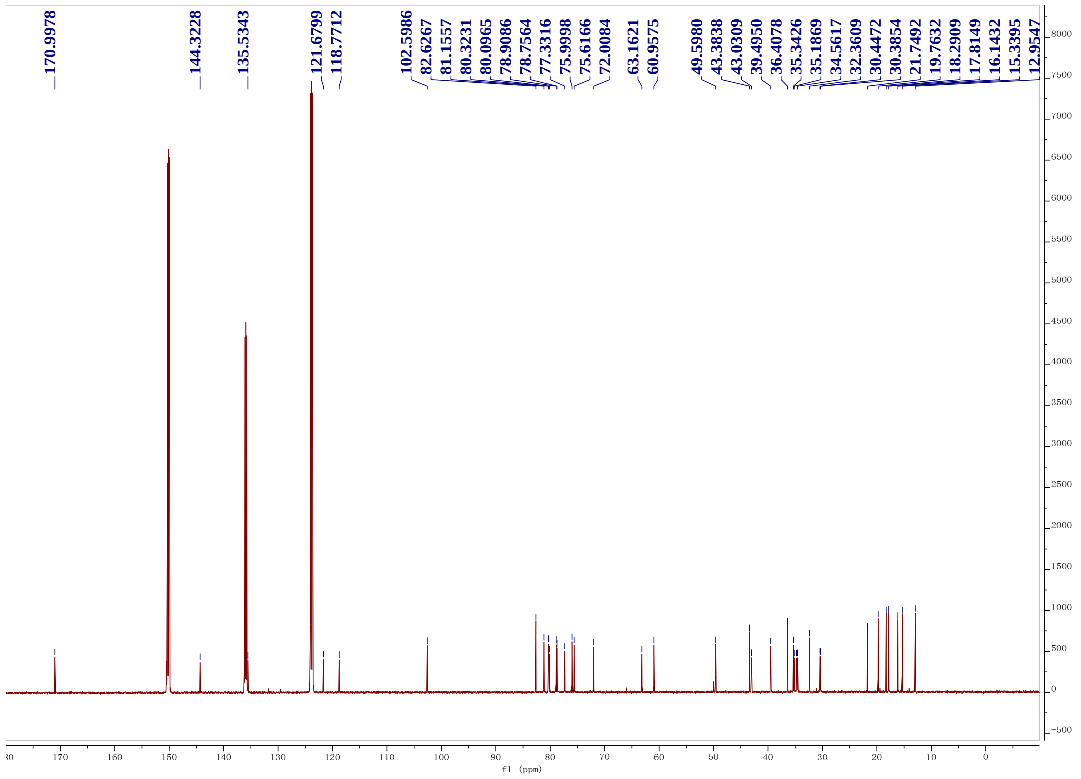

6.2.3 DEPT-135 spectrum of 4 in pyridine-d_5

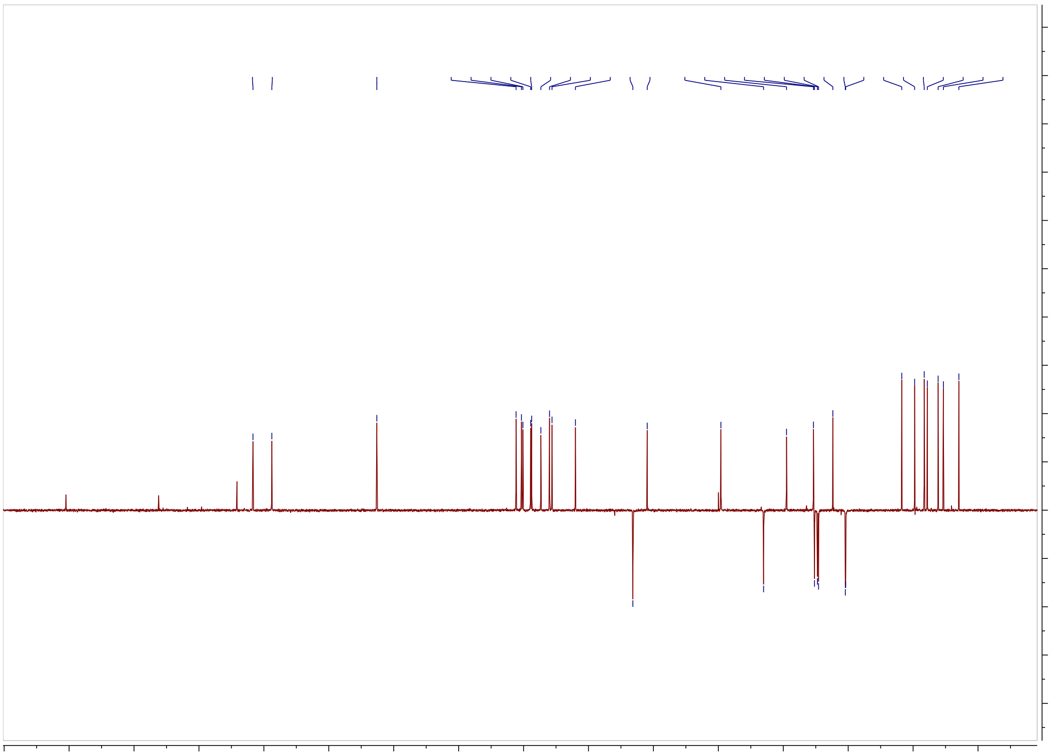

6.2.4 ^{1}H–^{1}H COSY spectrum of 4 in pyridine-d_5

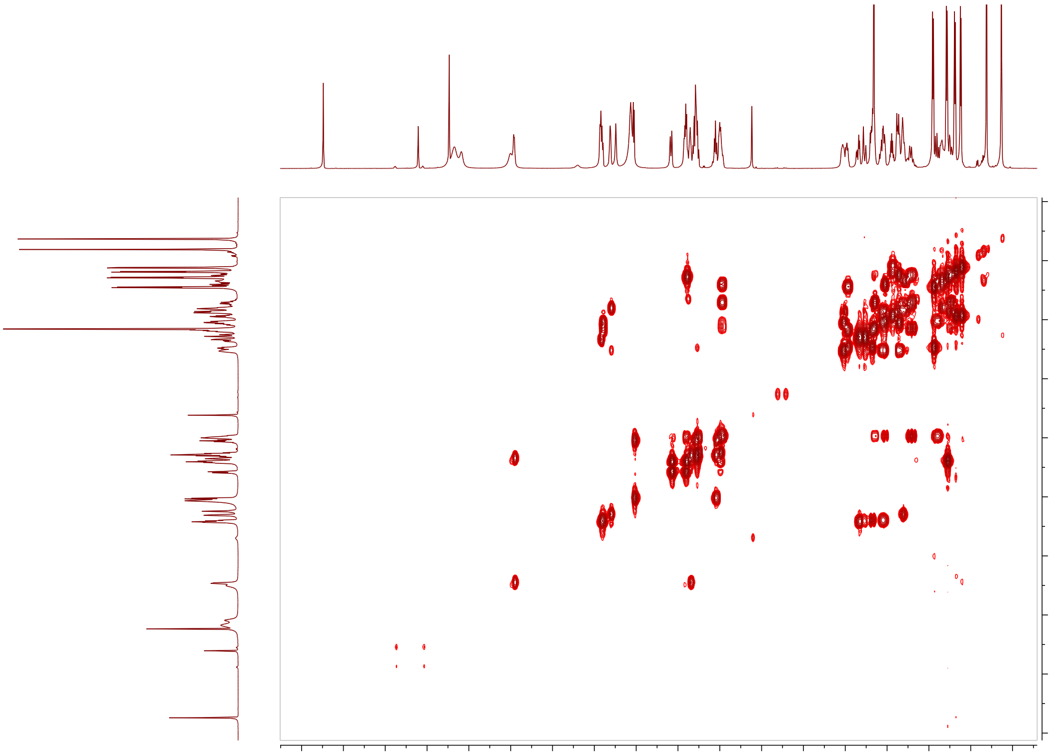

6.2.5 HSQC spectrum of 4 in pyridine-d_5

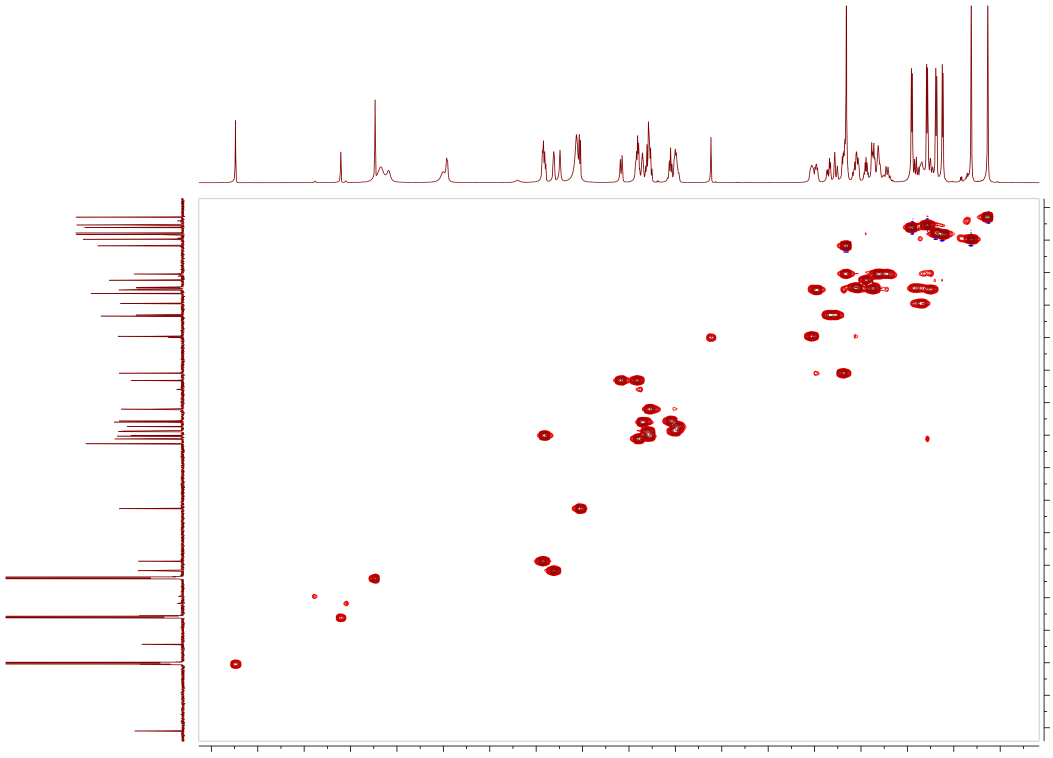

6.2.6 HMBC spectrum of 4 in pyridine-d_5

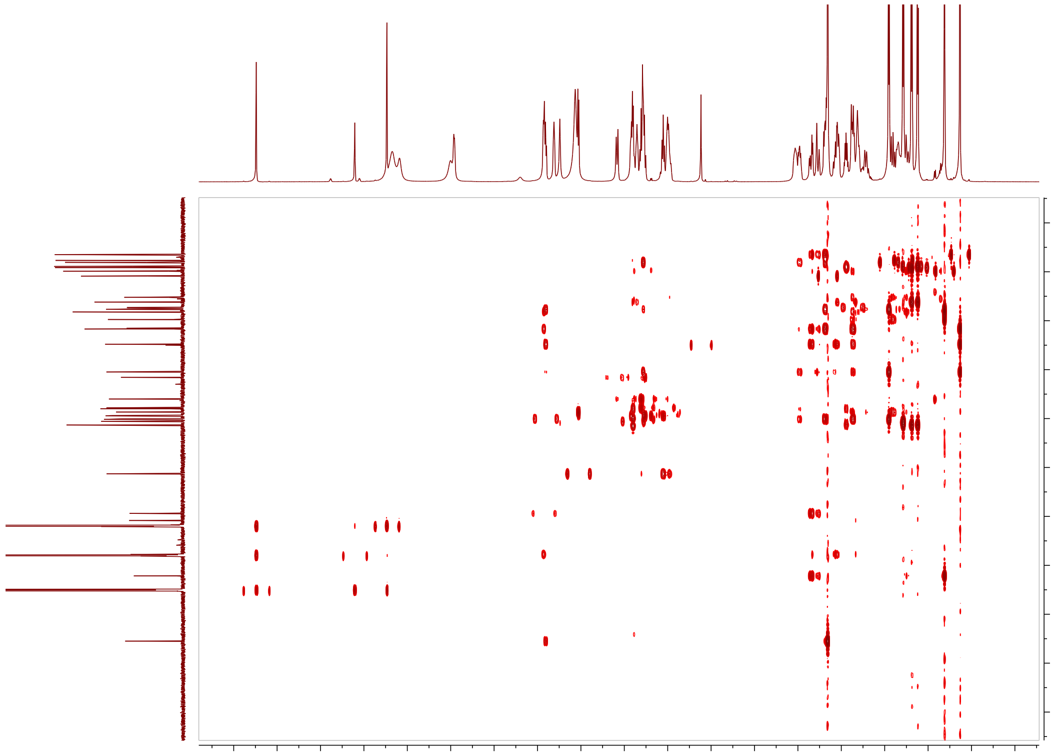

6.2.7 NOESY spectrum of 4 in pyridine-d_5

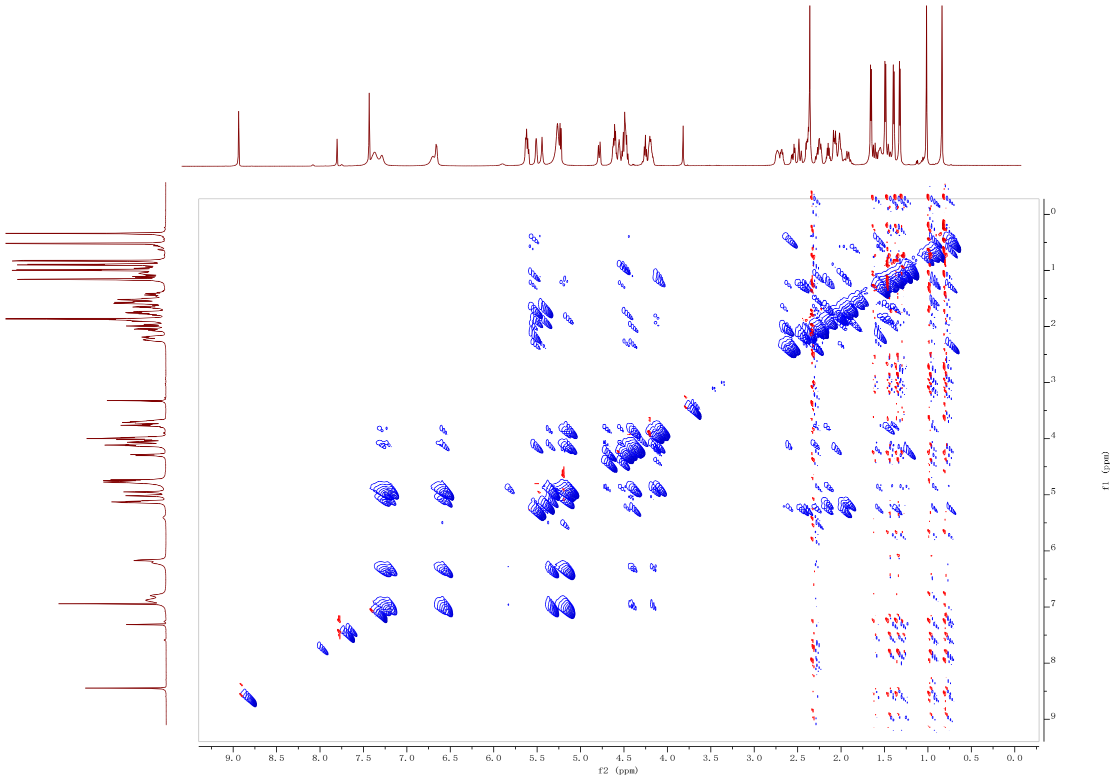
f2 (ppm)
f1 (ppm)

7. Spectral information of 5

7.1 HR-ESI-MS and UV (CH_3OH) spectrum of 5

HR-ESI-MS and UV (CH3OH) spectrum of 5

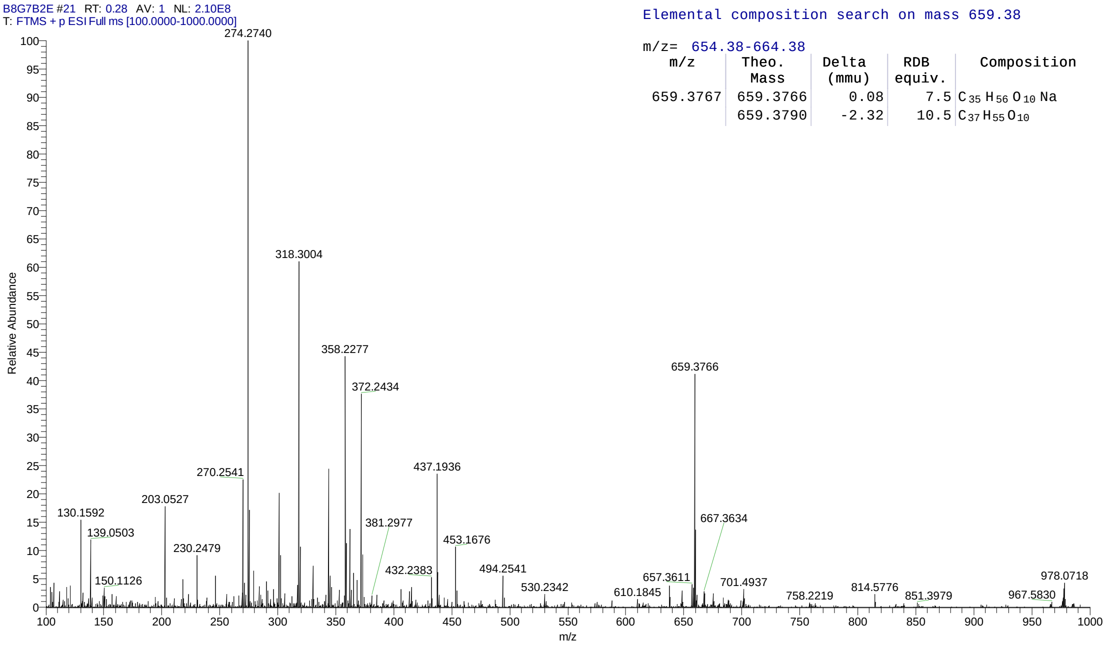

Elemental composition search on mass 659.38

m/z= 654.38-664.38

m/z	Theo. Mass	Delta (mmu)	RDB equiv.	Composition
659.3767	659.3766	0.08	7.5	$C_{35}H_{56}O_{10}Na$
	659.3790	-2.32	10.5	$C_{37}H_{55}O_{10}$

HR-ESI-MS of compound 5

Spectrum Peak Pick Report

2019-05-08 20:42:14

Data Set: B8G7B2E1A 0.0296 mg ml - RawData

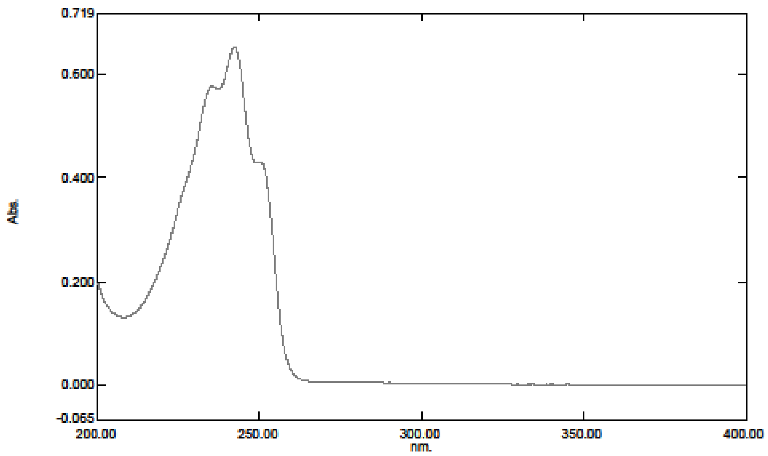

[Measurement Properties]
Wavelength Range (nm.): 200.00 to 400.00
Scan Speed: Medium
Sampling Interval: 0.2
Auto Sampling Interval: Enabled
Scan Mode: Single

No.	P/V	Wavelength	Abs.	Description
1		334.20	0.002	
2		320.40	0.003	
3		250.00	0.431	
4		242.40	0.653	
5		235.40	0.577	

[Instrument Properties]
Instrument Type: UV-2600 Series
Measuring Mode: Absorbance
Slit Width: 2.0
Accumulation time: 0.1 sec.
Light Source Change Wavelength: 323.0 nm
Detector Unit: Direct
S/R Exchange: Normal
Stair Correction: OFF

[Attachment Properties]
Attachment: None

[Operation]
Threshold: 0.0010000
Points: 4
InterPolate: Disabled
Average: Disabled

[Sample Preparation Properties]
Weight:
......

Page 1 / 1

UV spectrum of 5

7.2 1D and 2D NMR spectra of 5 in pyridine-d$_5$

7.2.1 ^{1}H NMR spectrum of 5 in pyridine-d$_5$

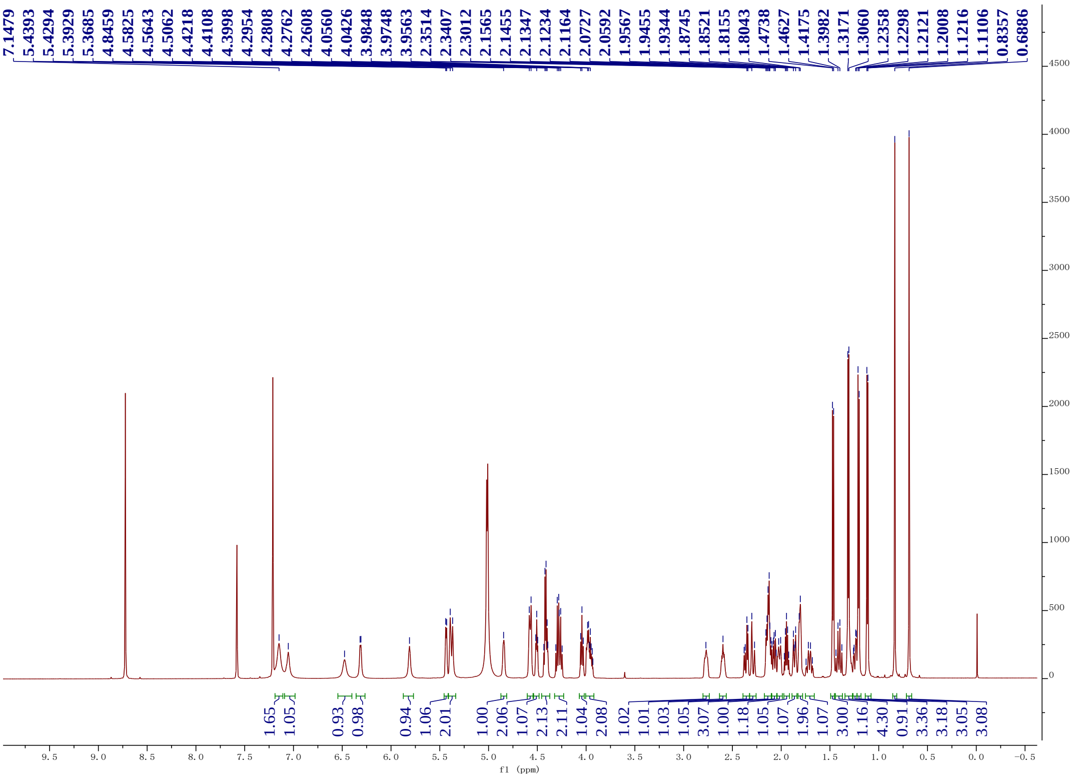

7.2.2 ^{13}C NMR spectrum of 5 in pyridine-d$_5$

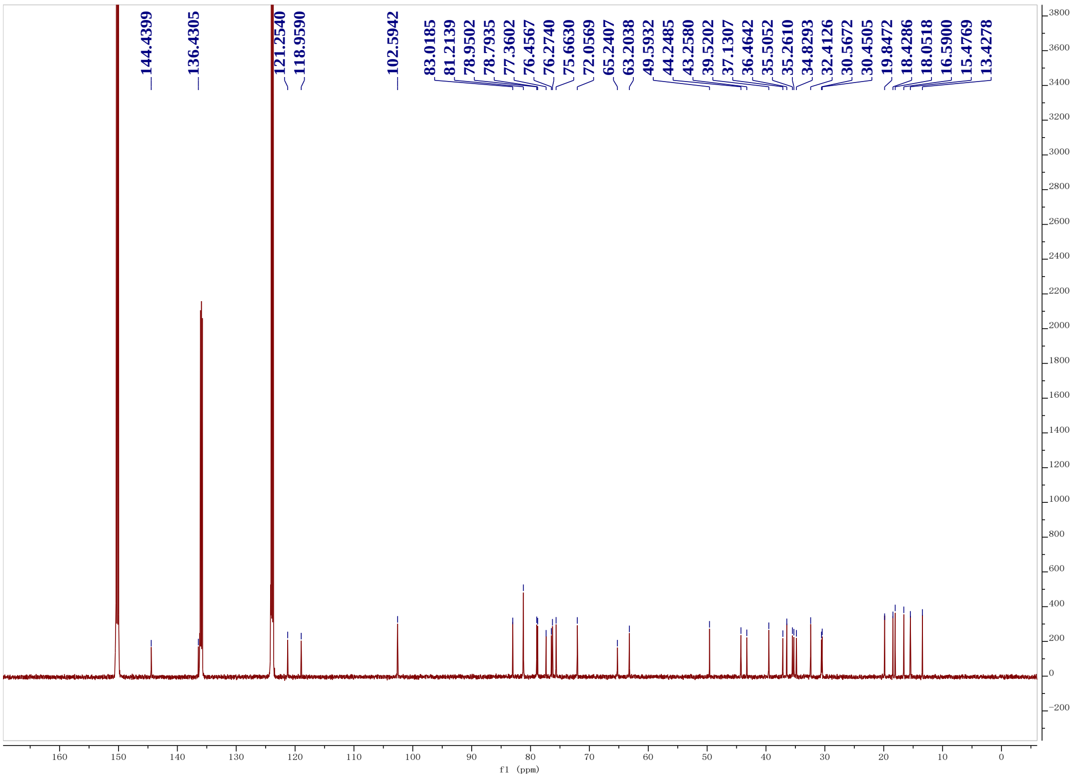

7.2.3 DEPT-135 spectrum of 5 in pyridine-d$_5$

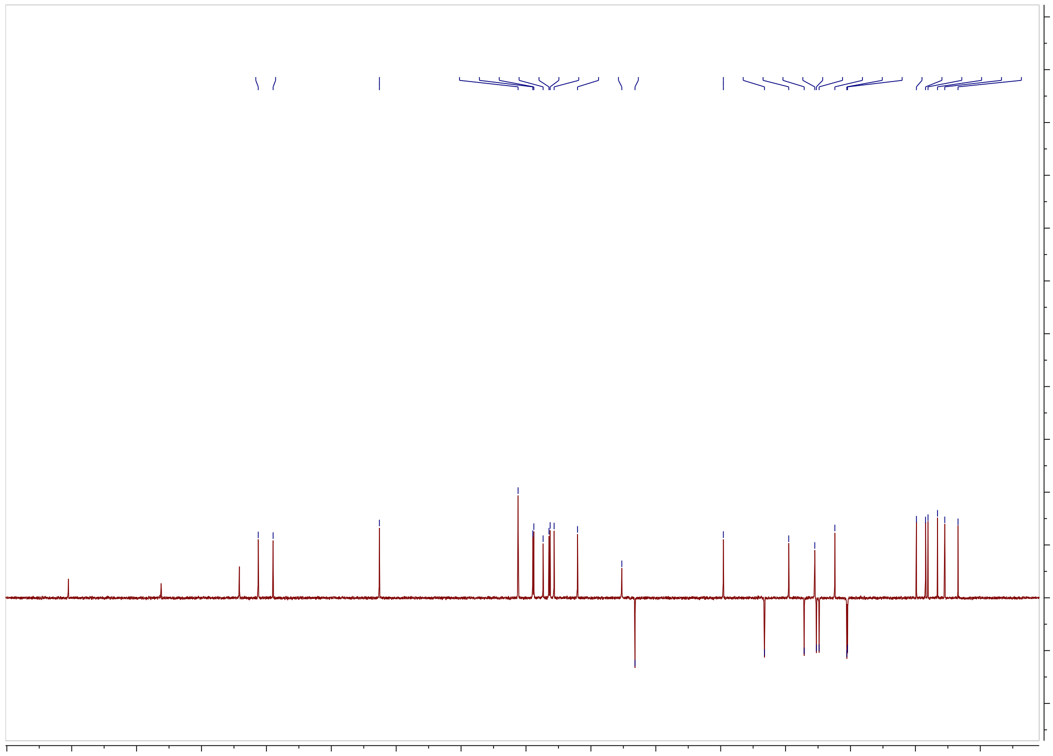

7.2.4 ^1H–^1H COSY spectrum of 5 in pyridine-d$_5$

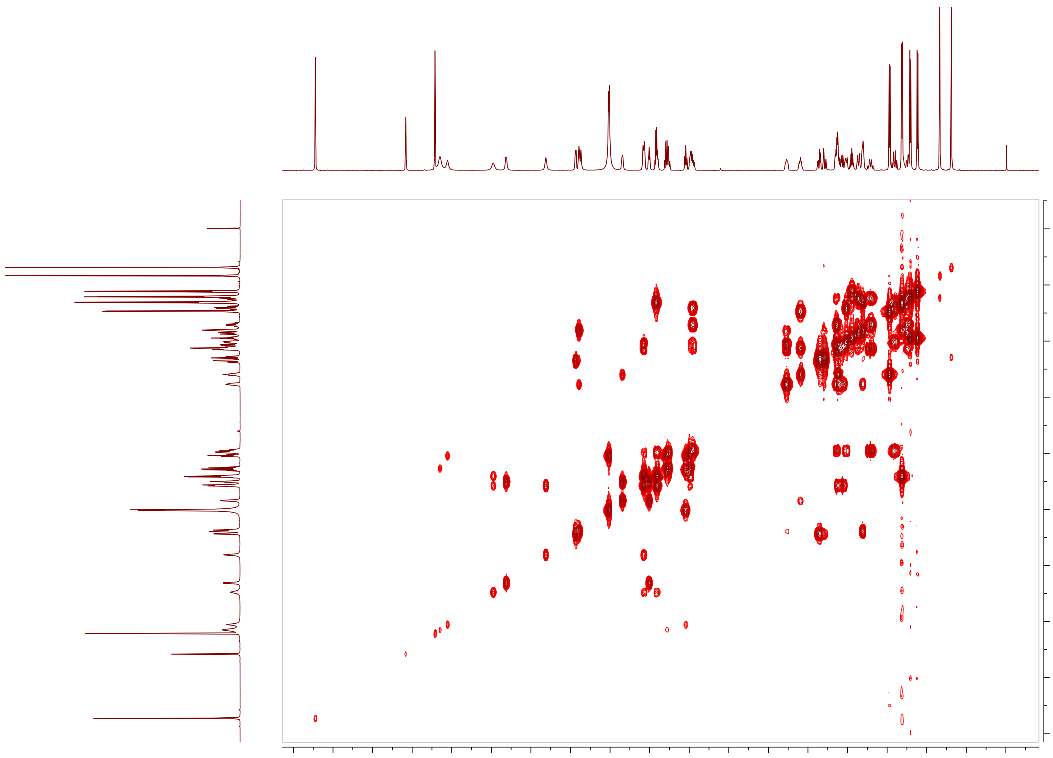

7.2.5 HSQC spectrum of 5 in pyridine-d_5

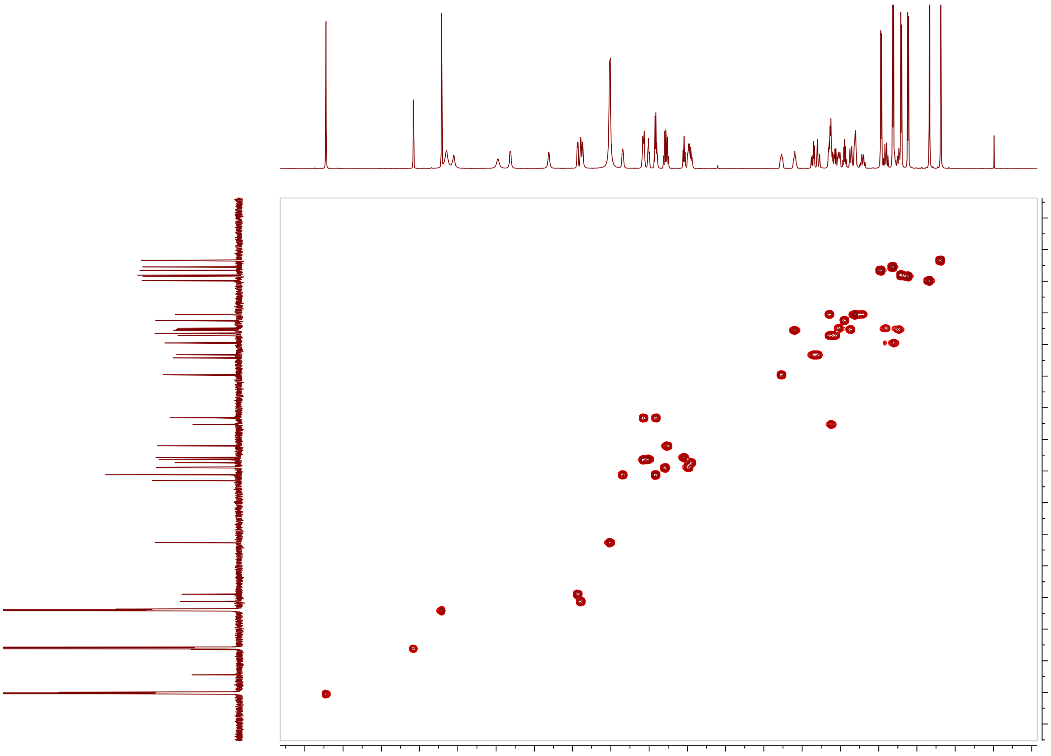

7.2.6 HMBC spectrum of 5 in pyridine-d_5

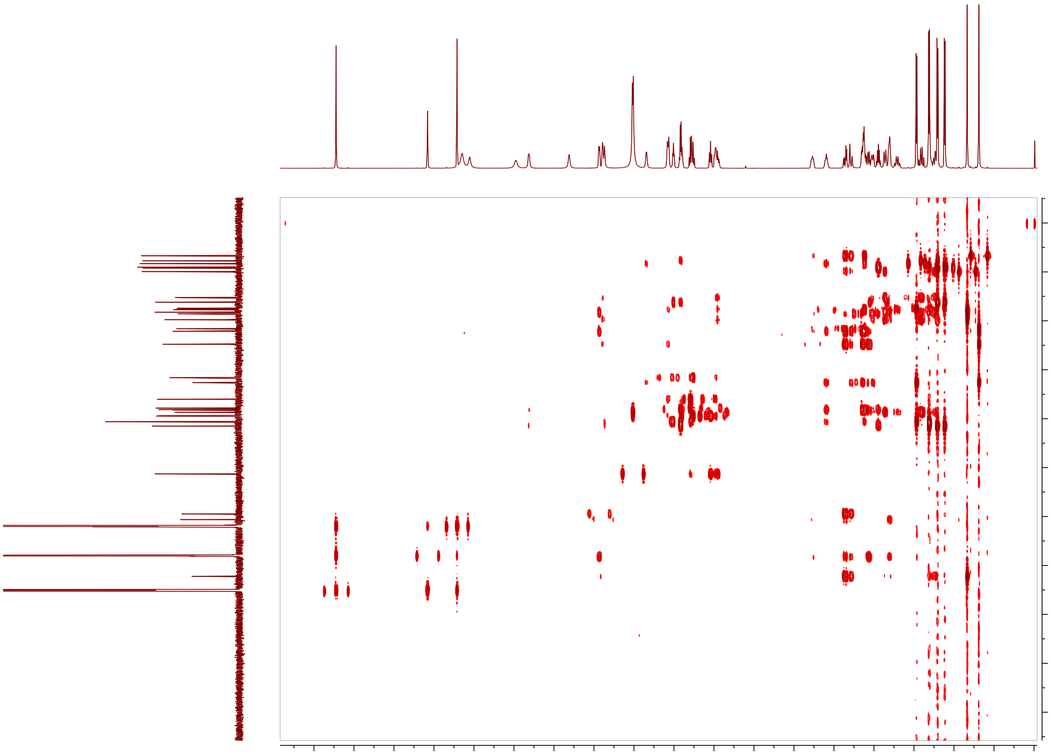

7.2.7 NOESY spectrum of 5 in pyridine-d_5

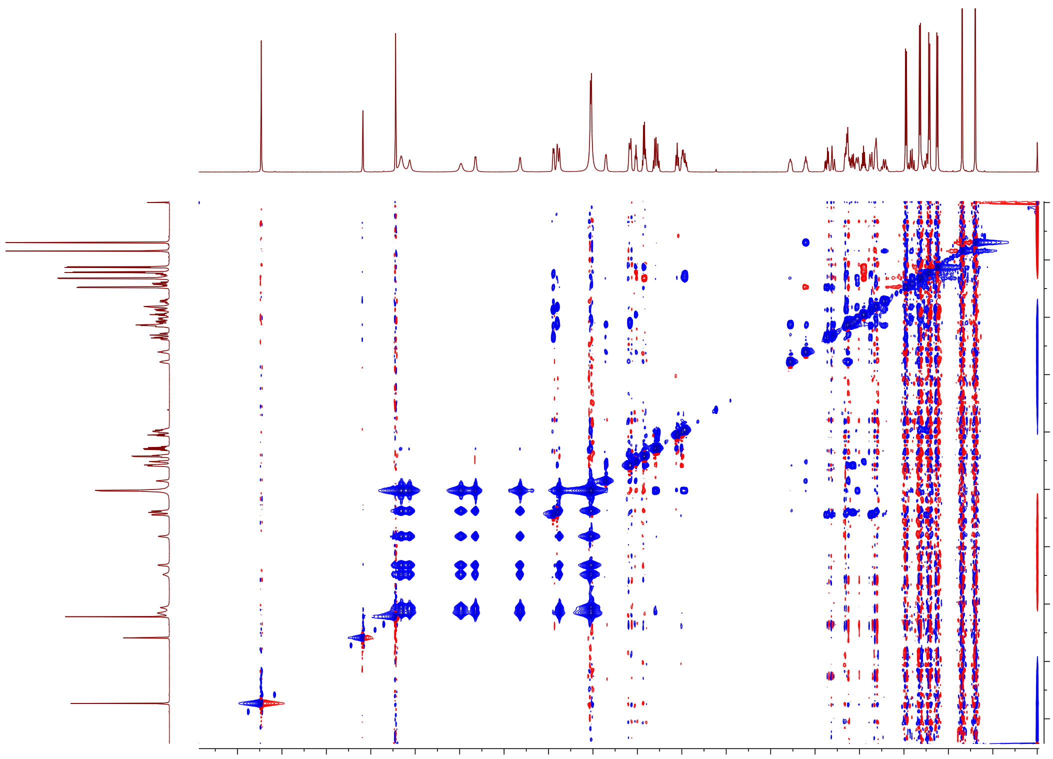

8. Spectral information of 6

8.1 HR-ESI-MS and UV (CH_3OH) spectrum of 6

HR-ESI-MS and UV (CH3OH) spectrum of 6

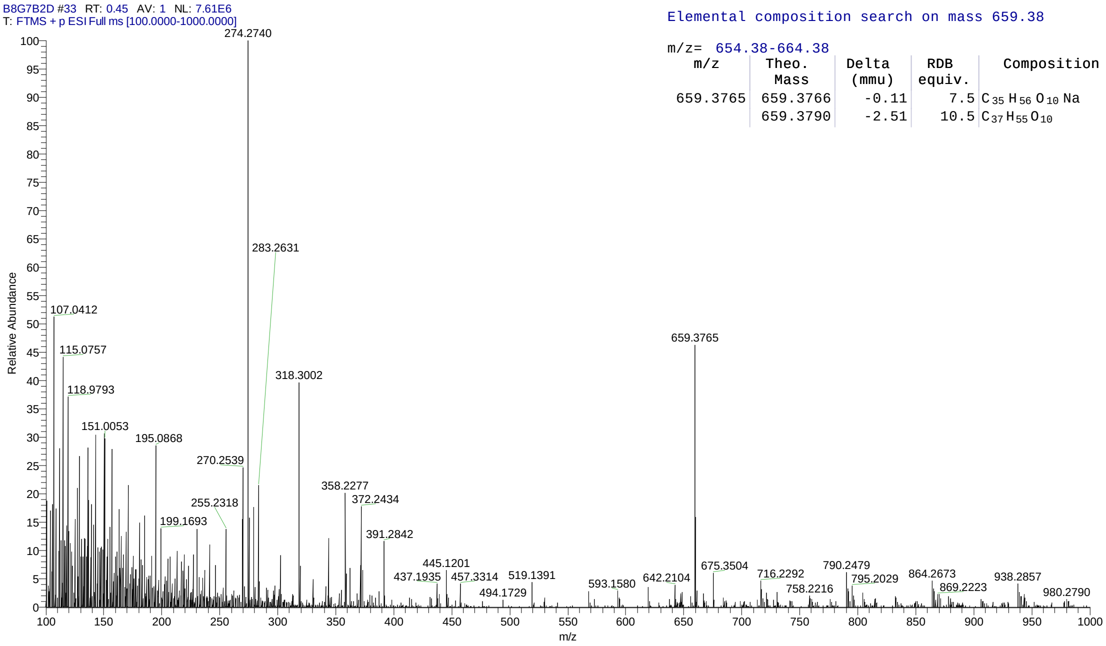

HR-ESI-MS of compound 6

Spectrum Peak Pick Report

2019-05-08 20:35:10

Data Set: B8G7B2D 0.0384 mg ml - RawData

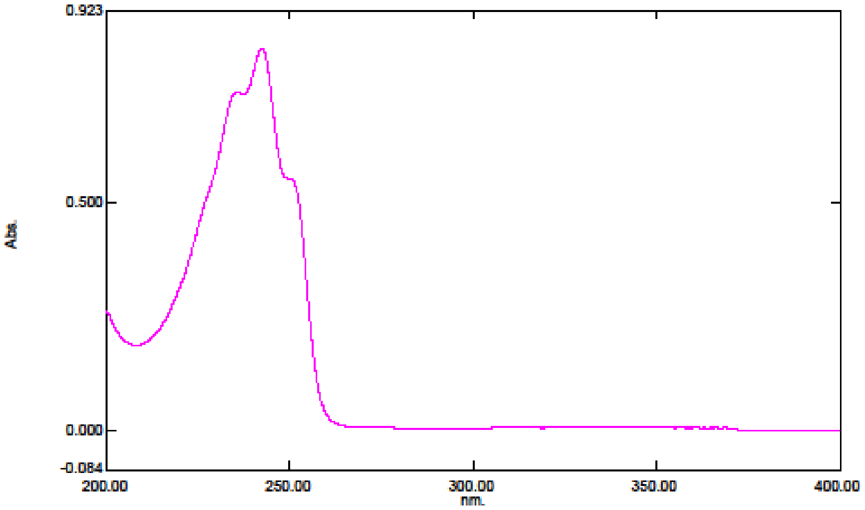

No.	P/V	Wavelength	Abs.	Description
1		370.80	0.006	
2		325.40	0.008	
3		308.00	0.009	
4		242.40	0.839	
5		235.60	0.744	

[Measurement Properties]
Wavelength Range (nm.): 200.00 to 400.00
Scan Speed: Medium
Sampling Interval: 0.2
Auto Sampling Interval: Enabled
Scan Mode: Single

[Instrument Properties]
Instrument Type: UV-2600 Series
Measuring Mode: Absorbance
Slit Width: 2.0
Accumulation time: 0.1 sec.
Light Source Change Wavelength: 323.0 nm
Detector Unit: Direct
S/R Exchange: Normal
Stair Correction: OFF

[Attachment Properties]
Attachment: None

[Operation]
Threshold: 0.0010000
Points: 4
InterPolate: Disabled
Average: Disabled

[Sample Preparation Properties]
Weight:
......

Page 1 / 1

UV spectrum of 6

8.2 1D and 2D NMR spectra of 6 in pyridine-d$_5$

8.2.1 ^{1}H NMR spectrum of 6 in pyridine-d$_5$

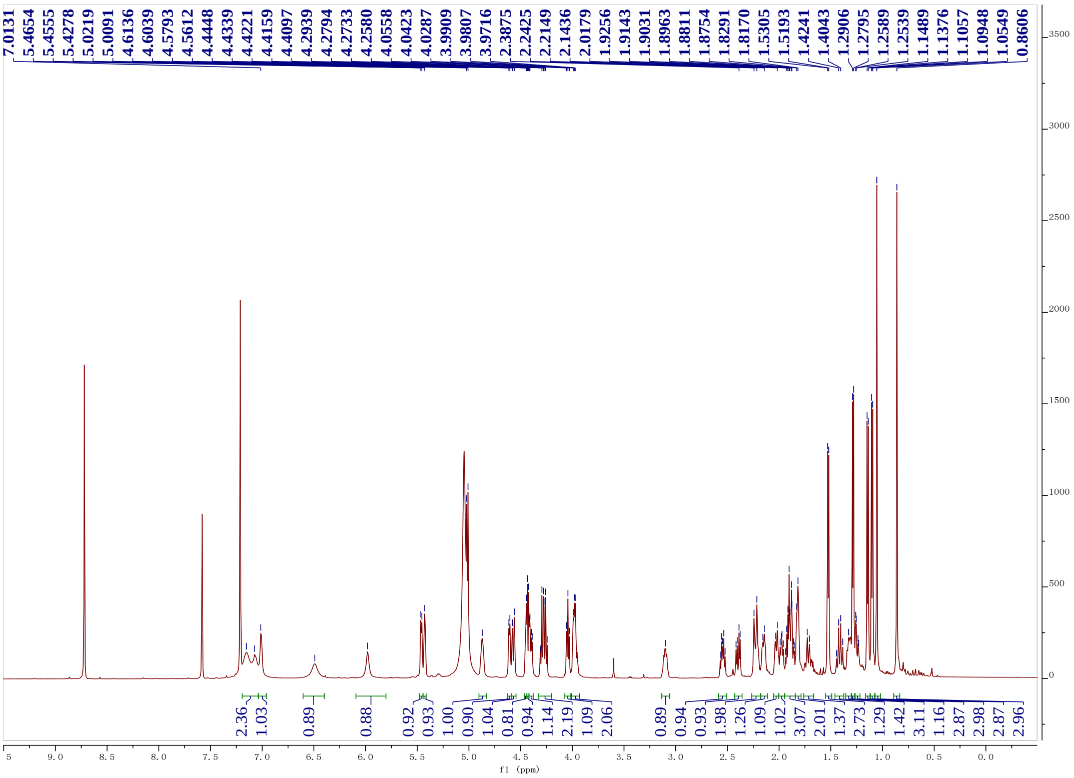

8.2.2 ^{13}C NMR spectrum of 6 in pyridine-d$_5$

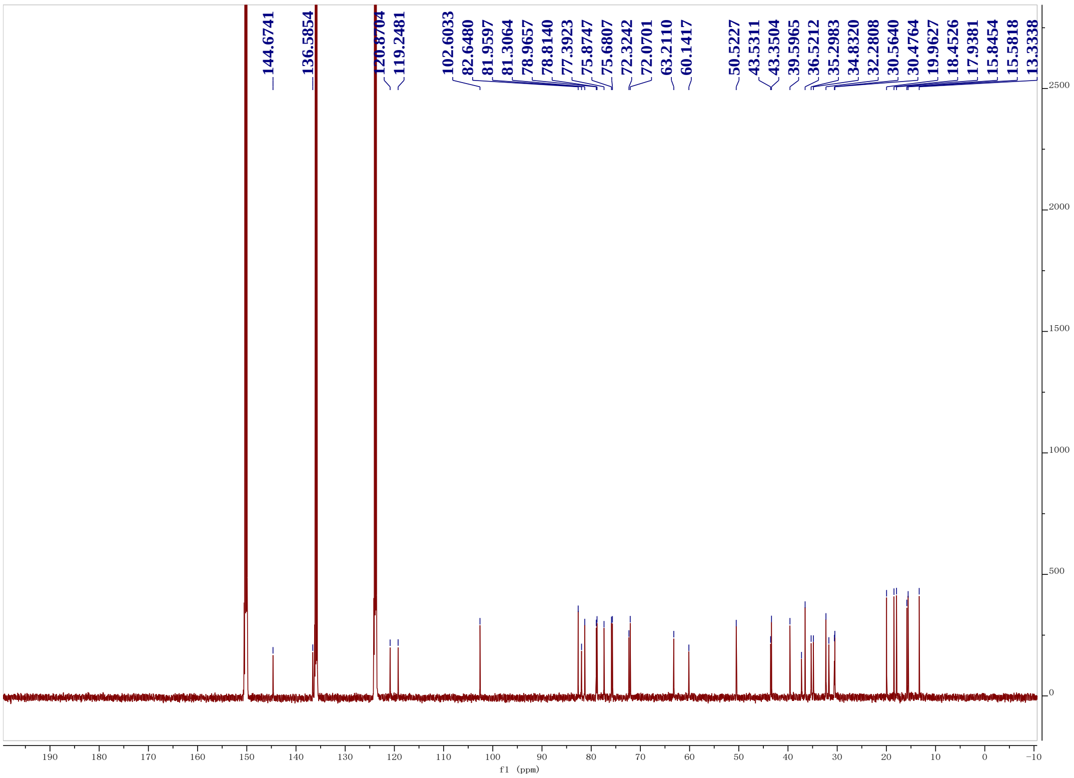

8.2.3 DEPT-135 spectrum of 6 in pyridine-d$_5$

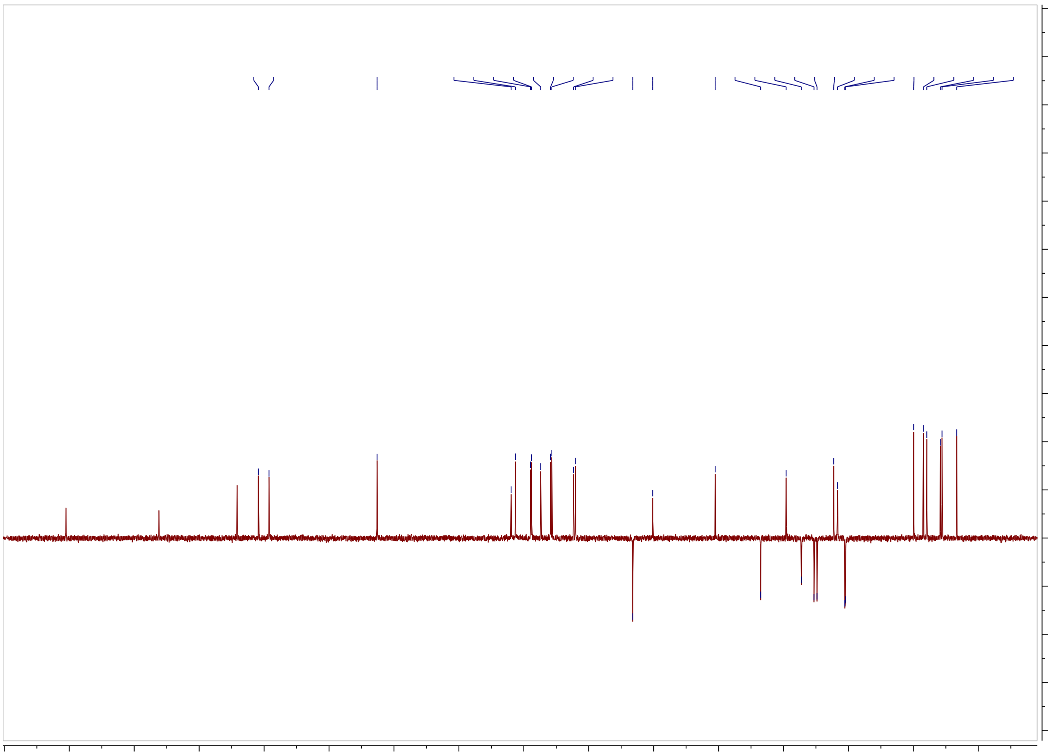

8.2.4 ^{1}H–^{1}H COSY spectrum of 6 in pyridine-d$_5$

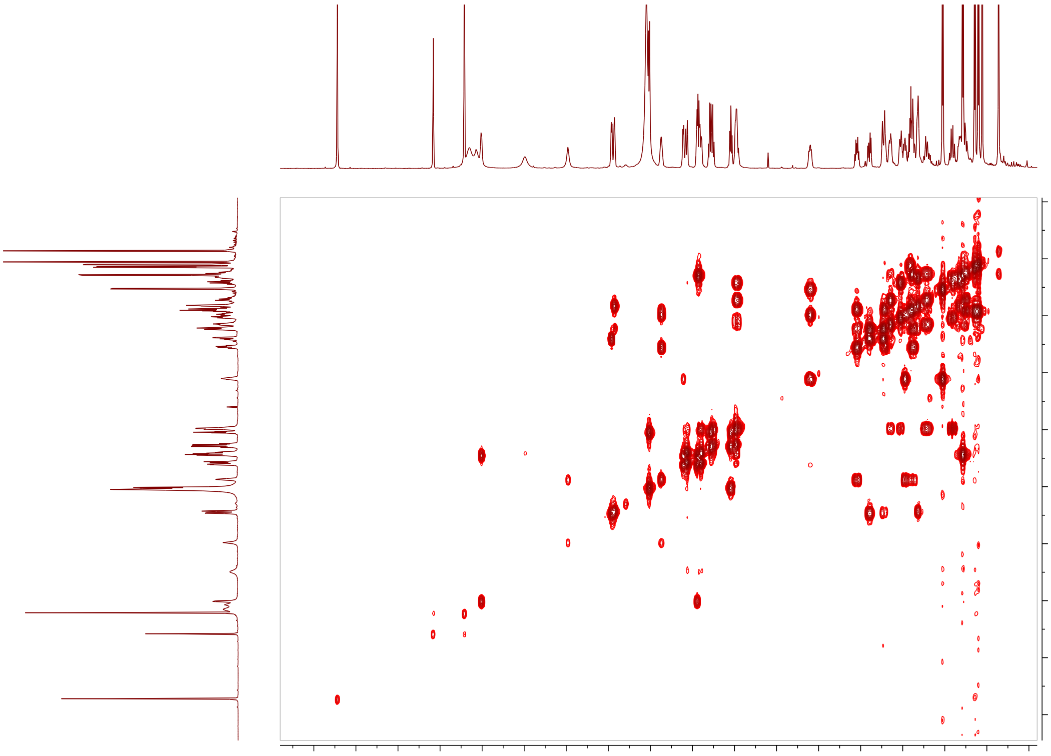

8.2.5 HSQC spectrum of 6 in pyridine-d_5

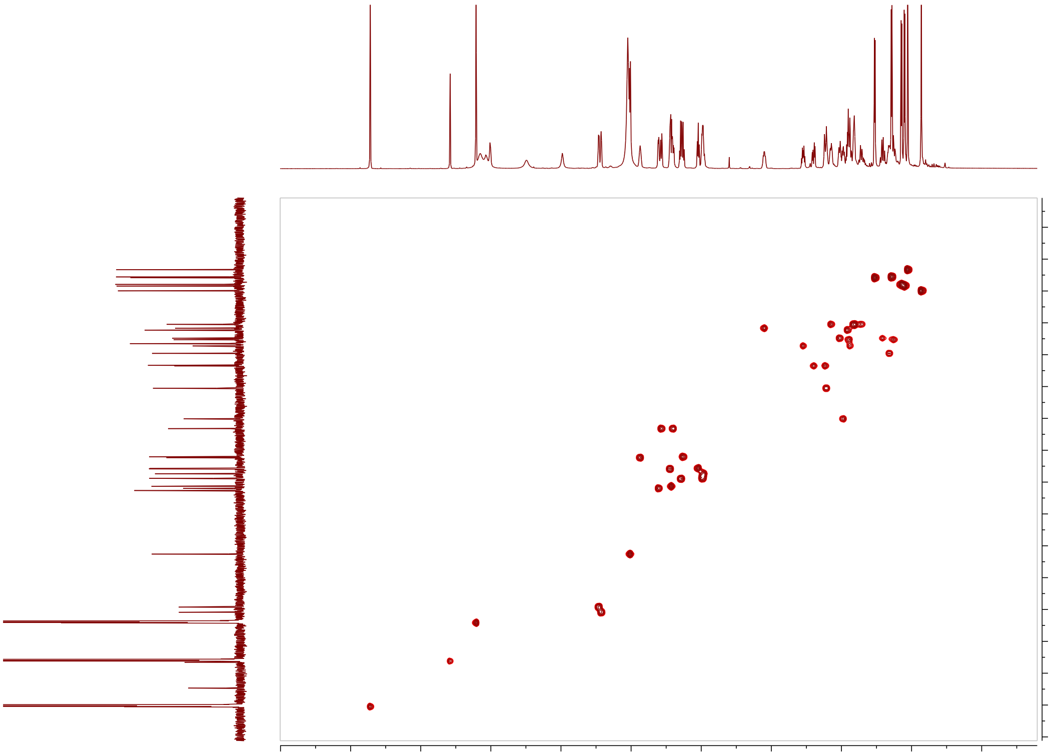

8.2.6 HMBC spectrum of 6 in pyridine-d_5

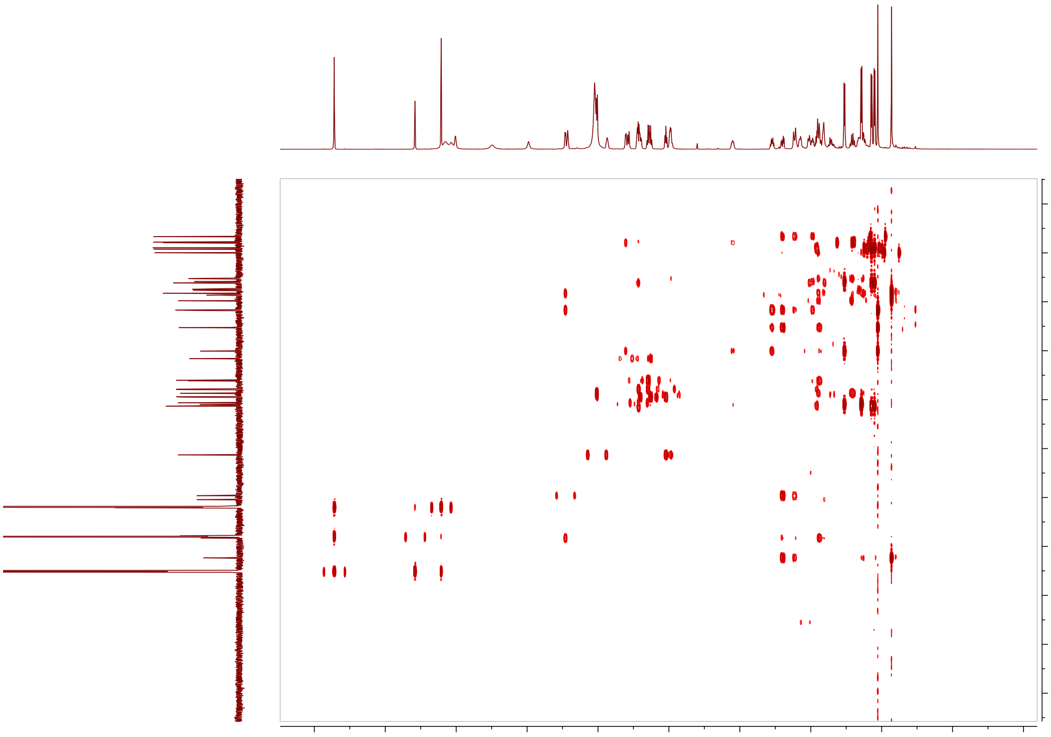

8.2.7 NOESY spectrum of 6 in pyridine-d$_5$

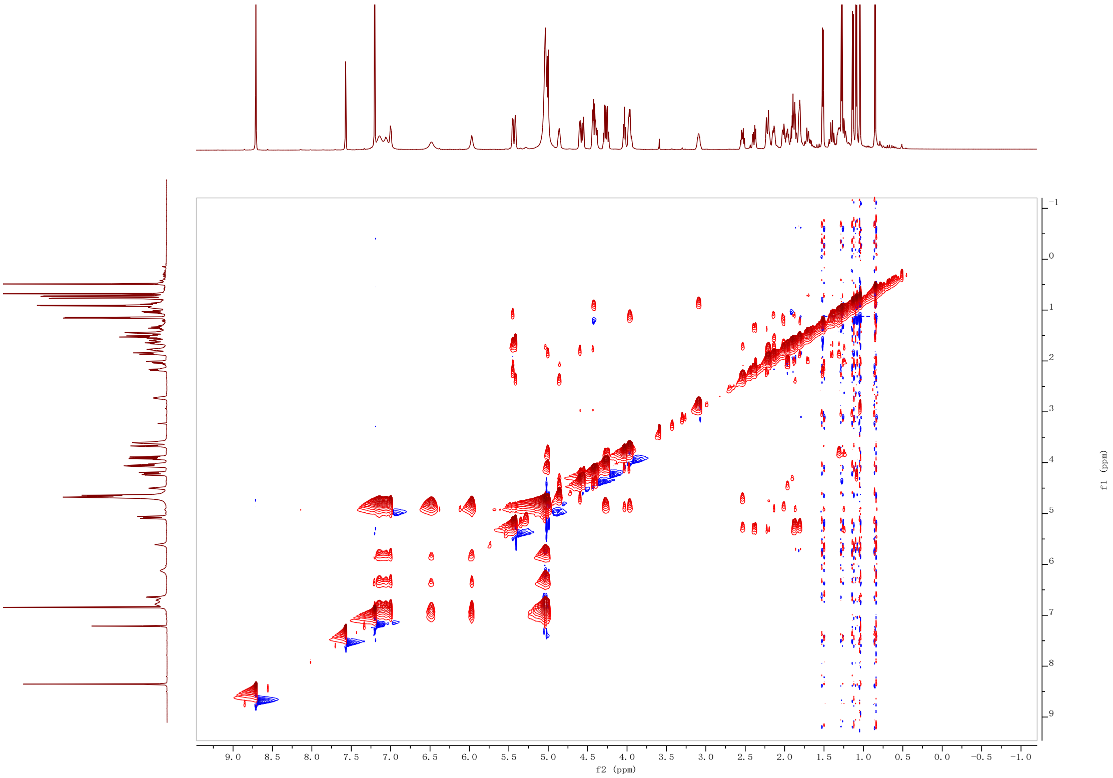

9. Spectral information of 7

9.1 HR-ESI-MS and UV (CH_3OH) spectrum of 7

HR-ESI-MS, IR and UV (CH3OH) spectrum of 7

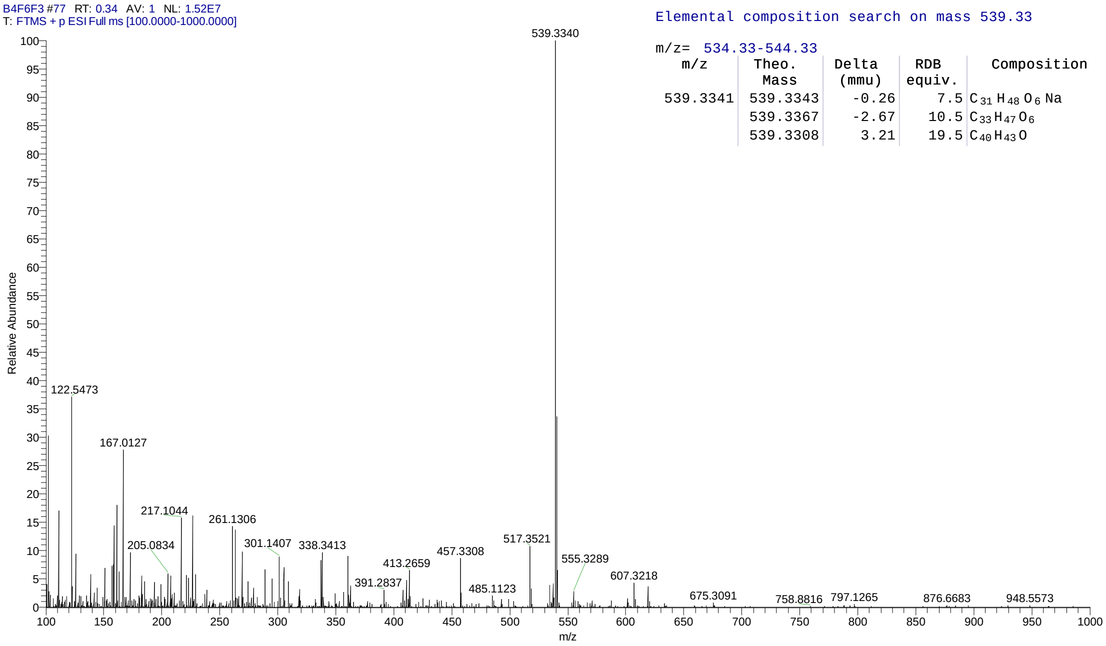

HR-ESI-MS of compound 7

Spectrum Peak Pick Report

2017-07-07 19:16:25

Data Set: B4F6F3 - RawData

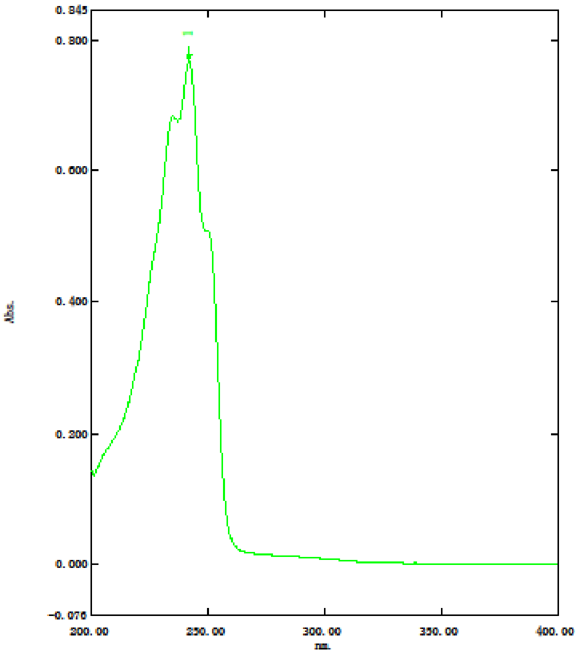

[Measurement Properties]
Wavelength Range (nm.): 200.00 to 400.00
Scan Speed: Medium
Sampling Interval: 1.0
Auto Sampling Interval: Disabled
Scan Mode: Auto

[Instrument Properties]
Instrument Type: UV-2600 Series
Measuring Mode: Absorbance
Slit Width: 2.0
Accumulation time: 0.1 sec.
Light Source Change Wavelength: 323.0 nm
Detector Unit: Direct
S/R Exchange: Normal
Stair Correction: OFF

[Attachment Properties]
Attachment: None

[Operation]
Threshold: 0.0010000
Points: 5
InterPolate: Disabled
Average: Disabled

[Sample Preparation Properties]
Weight:
Volume:
Dilution:
Path length:
Addition: Member:

No.	P/V	Wavelength (nm)	Absorbance	description
1	●	242.00	0.768	

页 1 / 1

UV spectrum of 7

9.2 1D and 2D NMR spectra of 7 in CD_3OD

9.2.1 ^{1}H NMR spectrum of 7 in CD_3OD

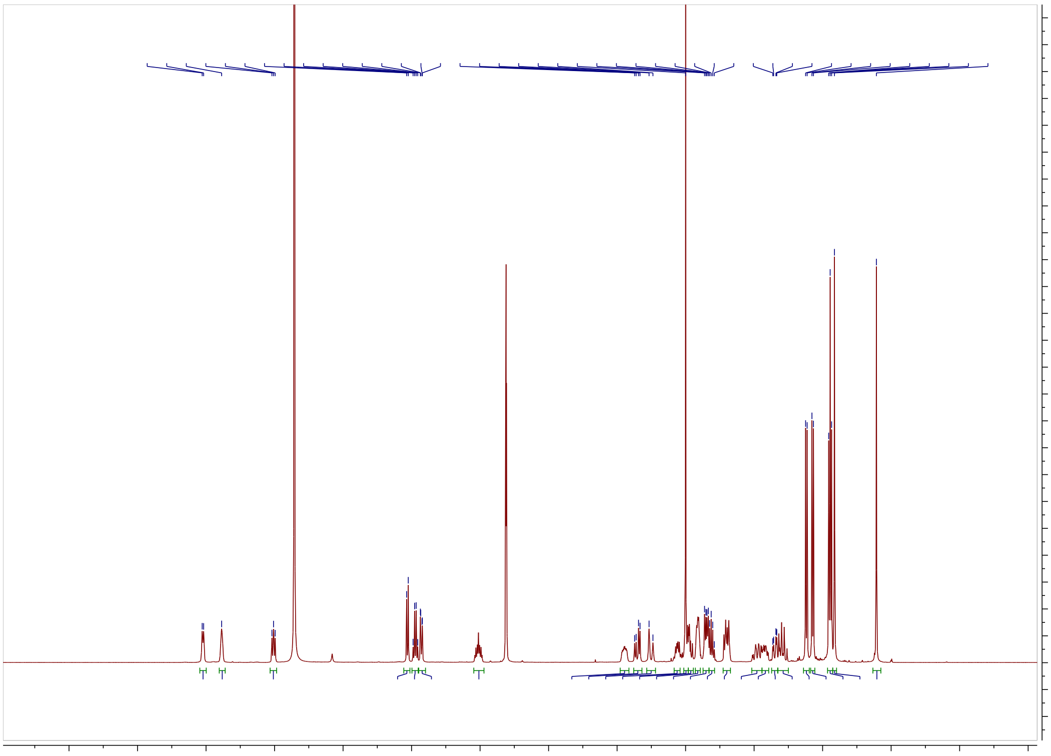

9.2.2 ^{13}C NMR spectrum of 7 in CD_3OD

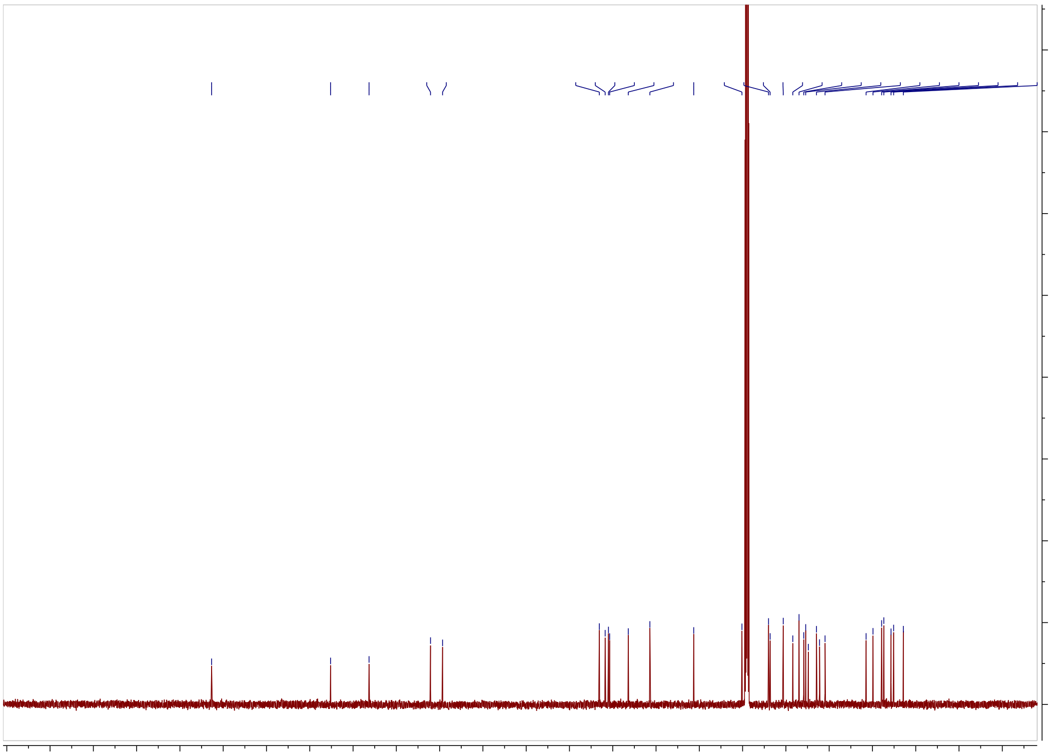

9.2.3 DEPT-135 spectrum of 7 in CD_3OD

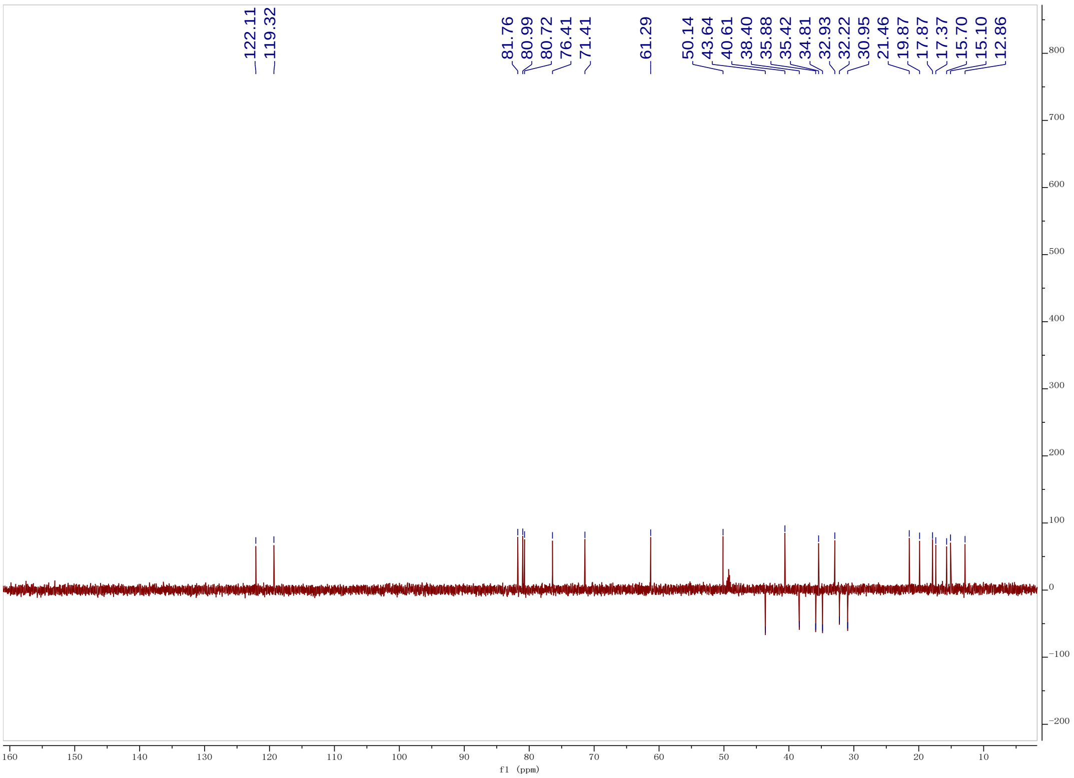

9.2.4 ^{1}H–^{1}H COSY spectrum of 7 in CD_3OD

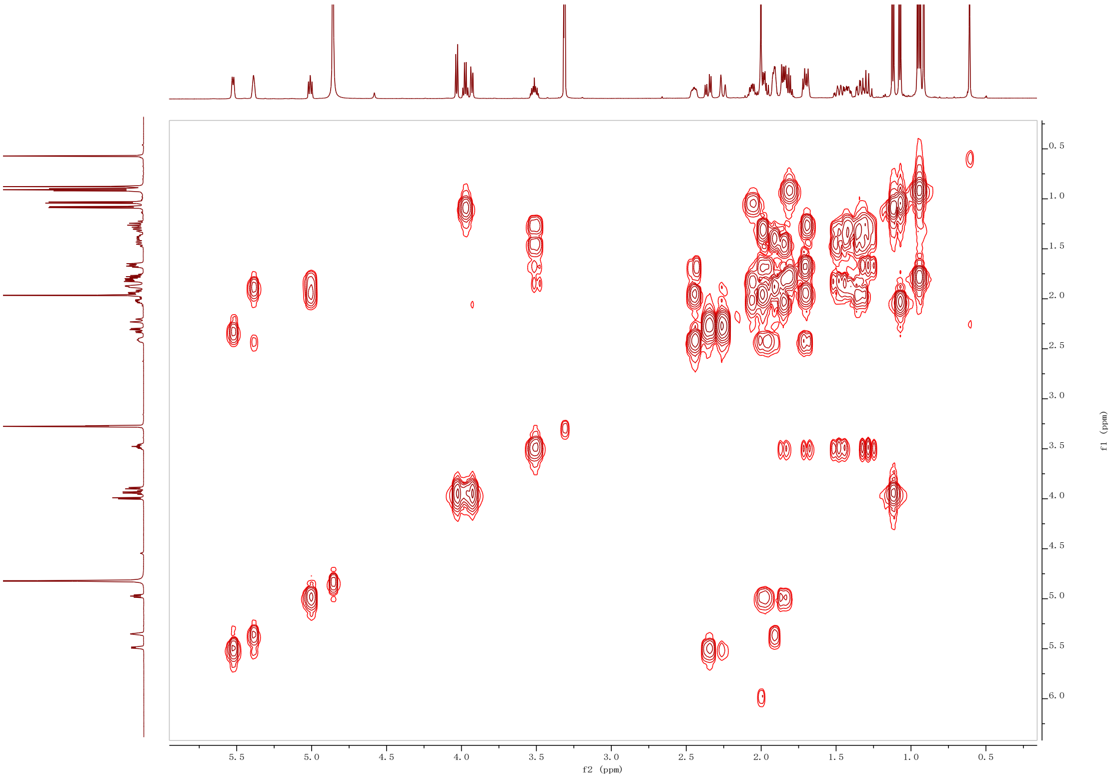

9.2.5 HSQC spectrum of 7 in CD_3OD

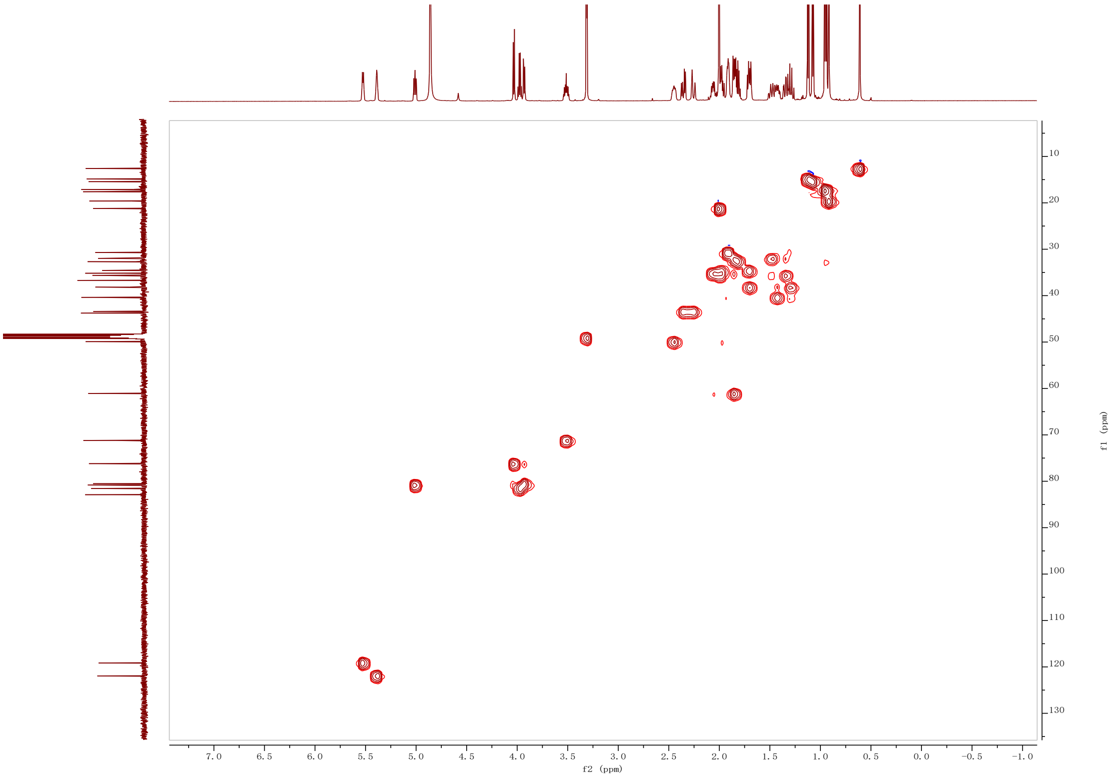

9.2.6 HMBC spectrum of 7 in CD_3OD

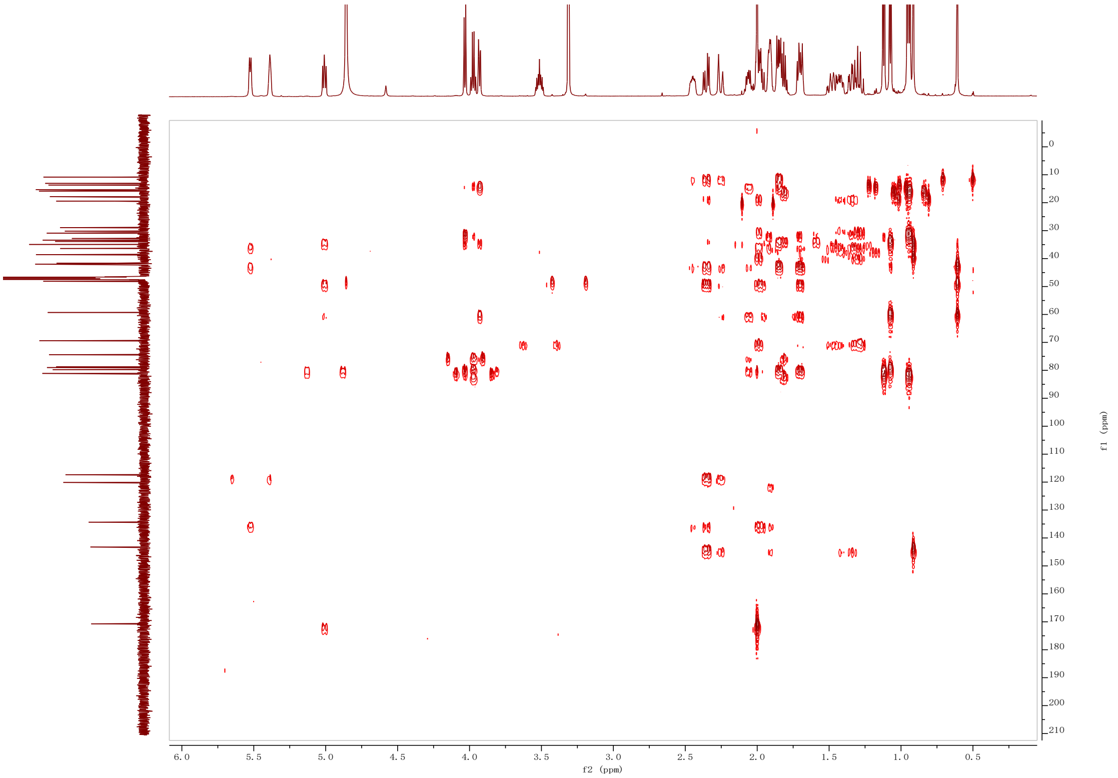

9.2.7 NOESY spectrum of 7 in CD_3OD

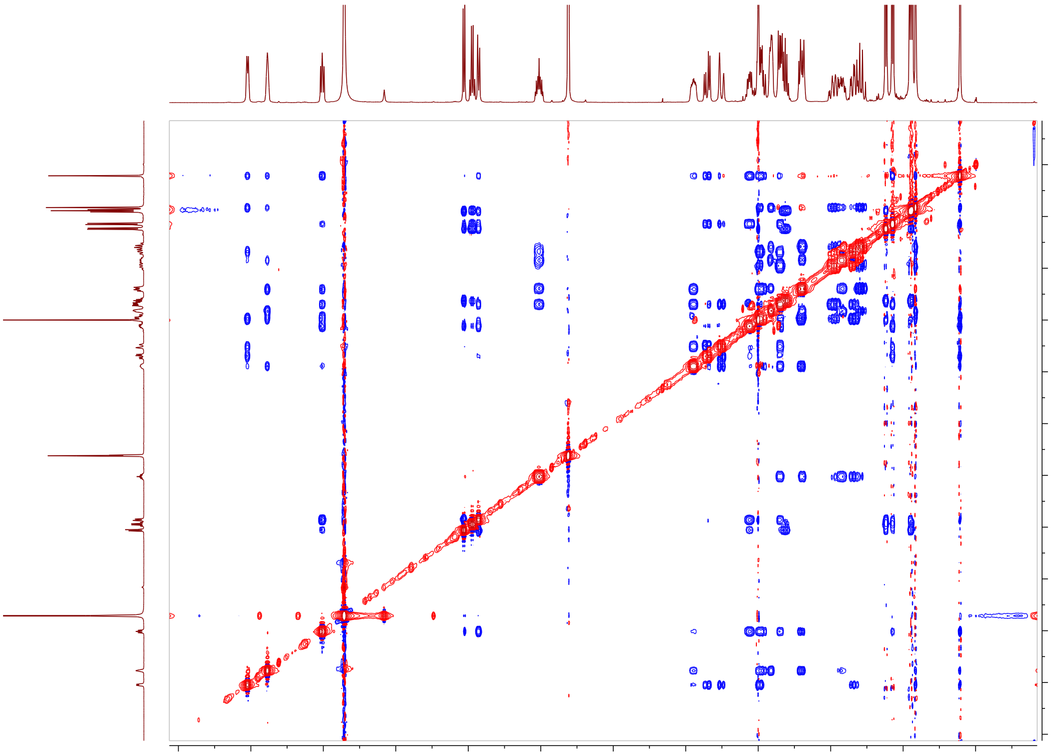

10. Spectral information of 8

10.1 HR-ESI-MS and UV (CH_3OH) spectrum of 8

HR-ESI-MS and UV (CH3OH) spectrum of 8

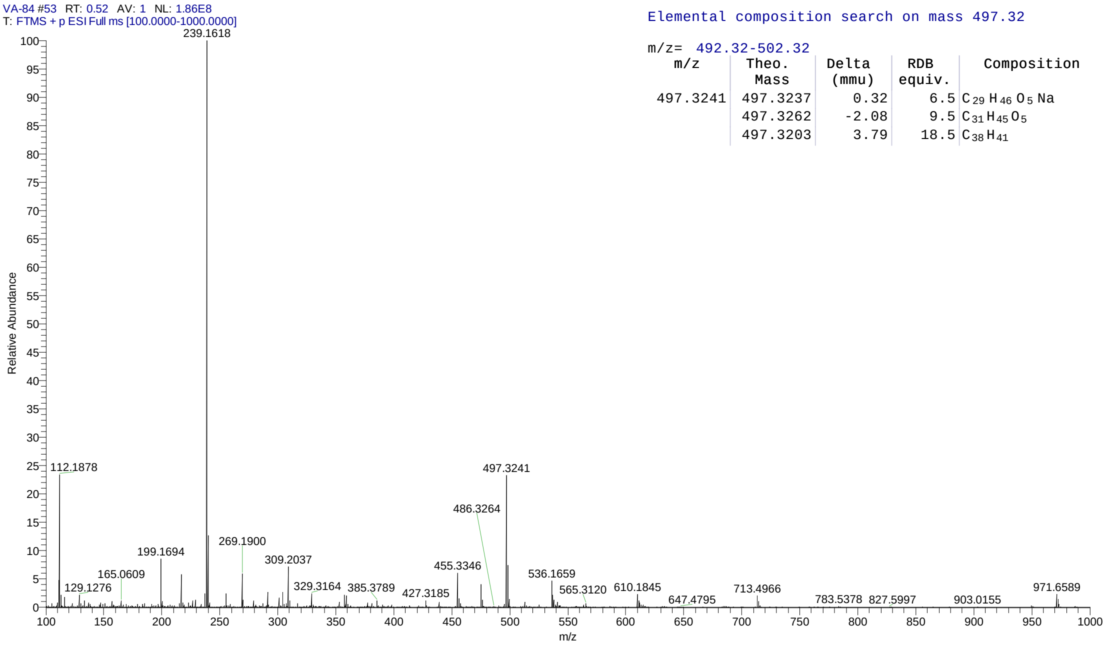

Elemental composition search on mass 497.32

m/z= 492.32-502.32

m/z	Theo. Mass	Delta (mmu)	RDB equiv.	Composition
497.3241	497.3237	0.32	6.5	$C_{29}H_{46}O_5Na$
	497.3262	-2.08	9.5	$C_{31}H_{45}O_5$
	497.3203	3.79	18.5	$C_{38}H_{41}$

HR-ESI-MS of compound 8

Spectrum Peak Pick Report

2019-11-07 21:52:27

Data Set: VA-84 0.0242 mg ml - RawData

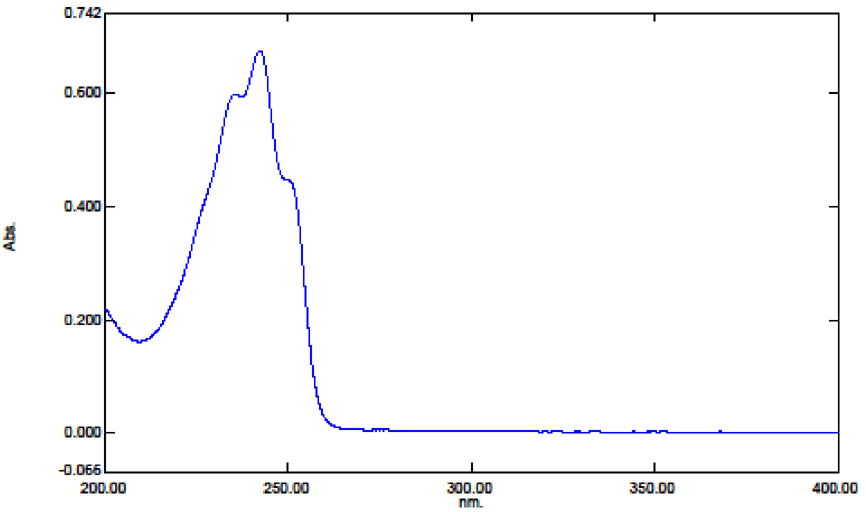

[Measurement Properties]
Wavelength Range (nm.): 200.00 to 400.00
Scan Speed: Medium
Sampling Interval: 0.5
Auto Sampling Interval: Disabled
Scan Mode: Auto

[Instrument Properties]
Instrument Type: UV-2600 Series
Measuring Mode: Absorbance
Slit Width: 2.0
Accumulation time: 0.1 sec.
Light Source Change Wavelength: 323.0 nm
Detector Unit: Direct
S/R Exchange: Normal
Stair Correction: OFF

[Attachment Properties]
Attachment: None

[Operation]
Threshold: 0.0010000
Points: 4
InterPolate: Disabled
Average: Disabled

[Sample Preparation Properties]
Weight:
......

No.	P/V	Wavelength	Abs.	Description
1	P	368.00	0.003	
2	P	333.50	0.003	
3	P	329.50	0.003	
4	P	242.50	0.674	
5	P	235.50	0.597	
6	V	331.50	0.001	
7	V	325.50	0.001	
8	V	237.50	0.593	
9	V	209.50	0.162	

Page 1 / 1

UV spectrum of 8

10.2 1D and 2D NMR spectra of 8 in pyridine-d$_5$

10.2.1 ^{1}H NMR spectrum of 8 in pyridine-d$_5$

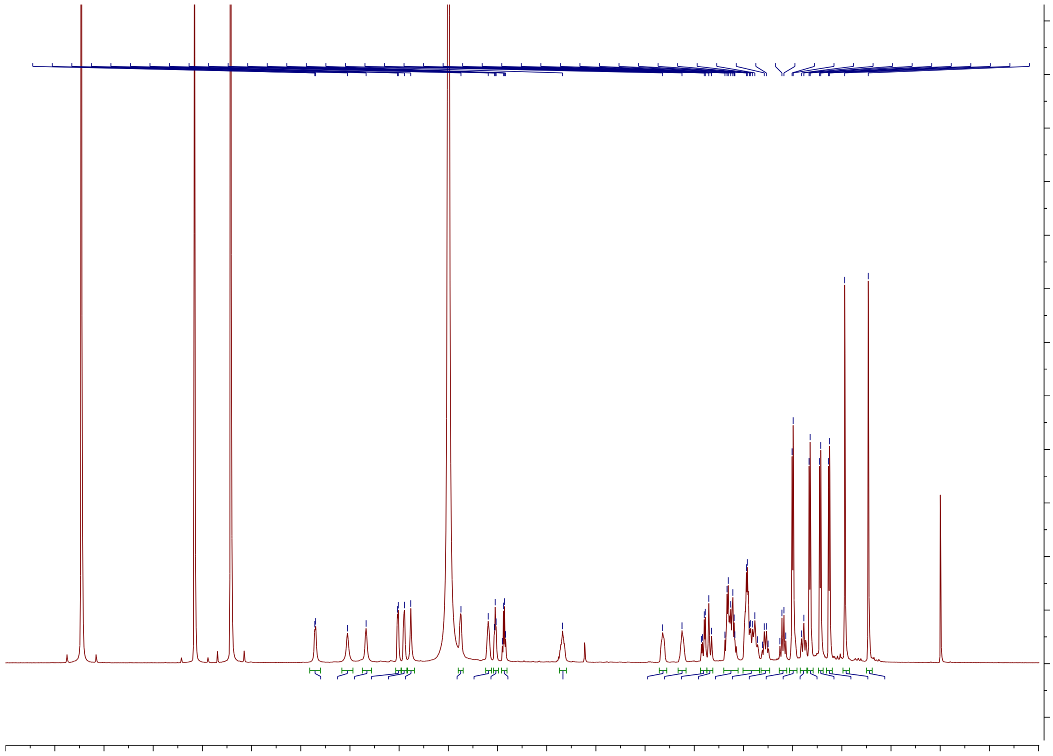

10.2.2 ^{13}C NMR spectrum of 8 in pyridine-d$_5$

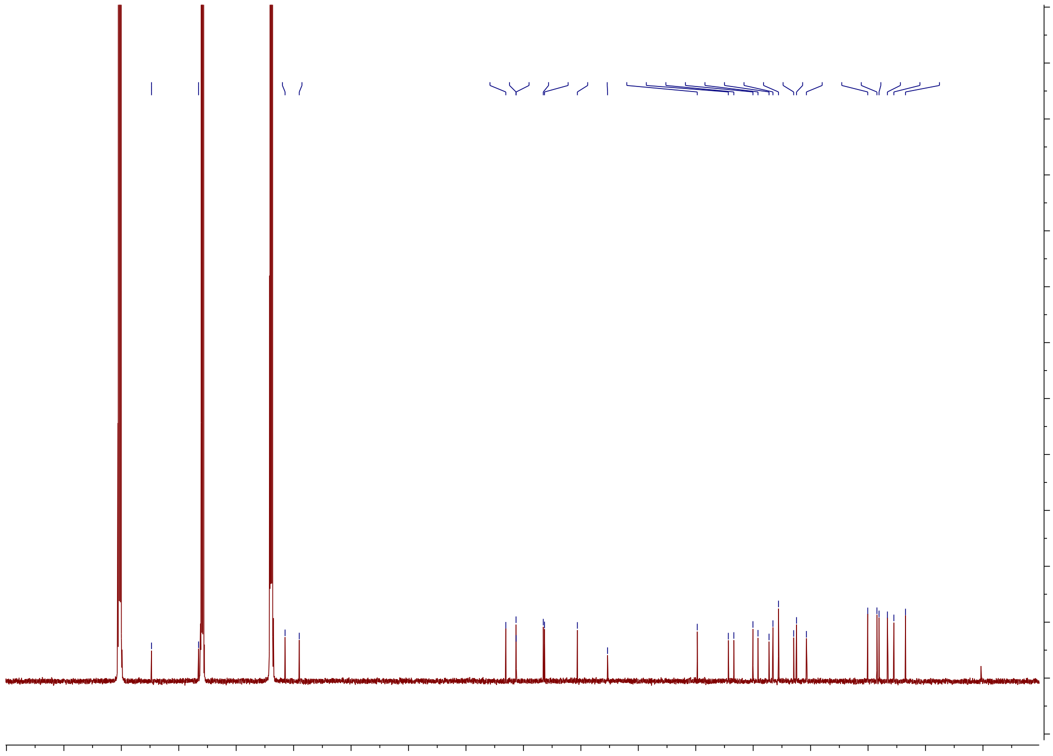

10.2.3 DEPT-135 spectrum of 8 in pyridine-d_5

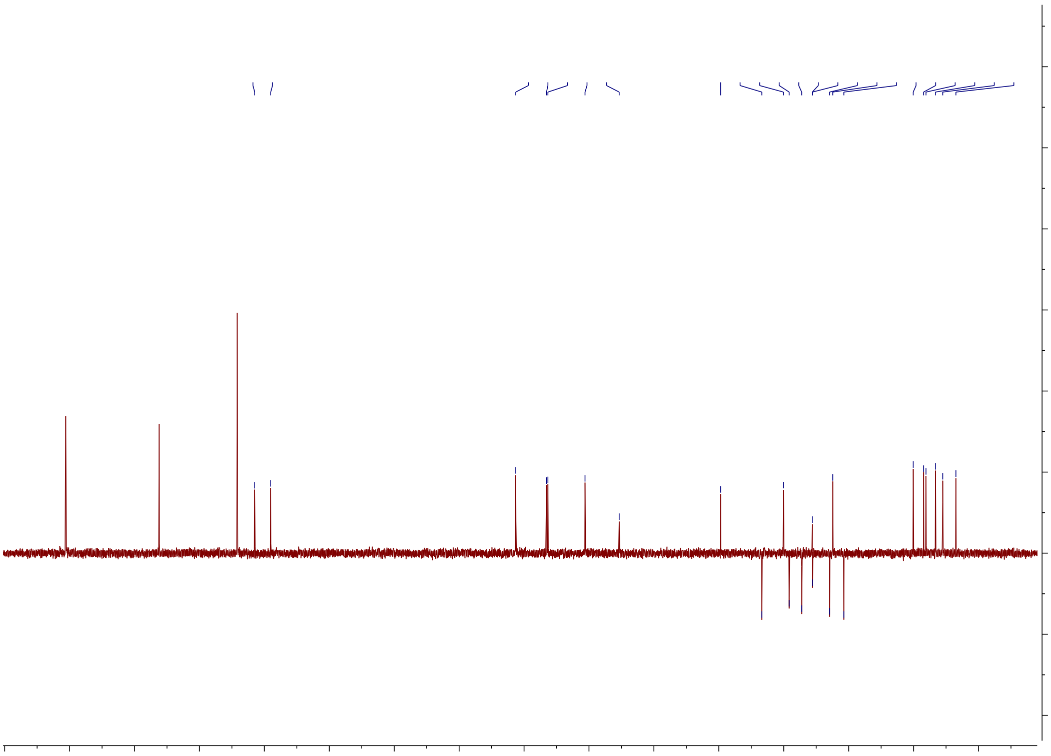

10.2.4 ^{1}H–^{1}H COSY spectrum of 8 in pyridine-d_5

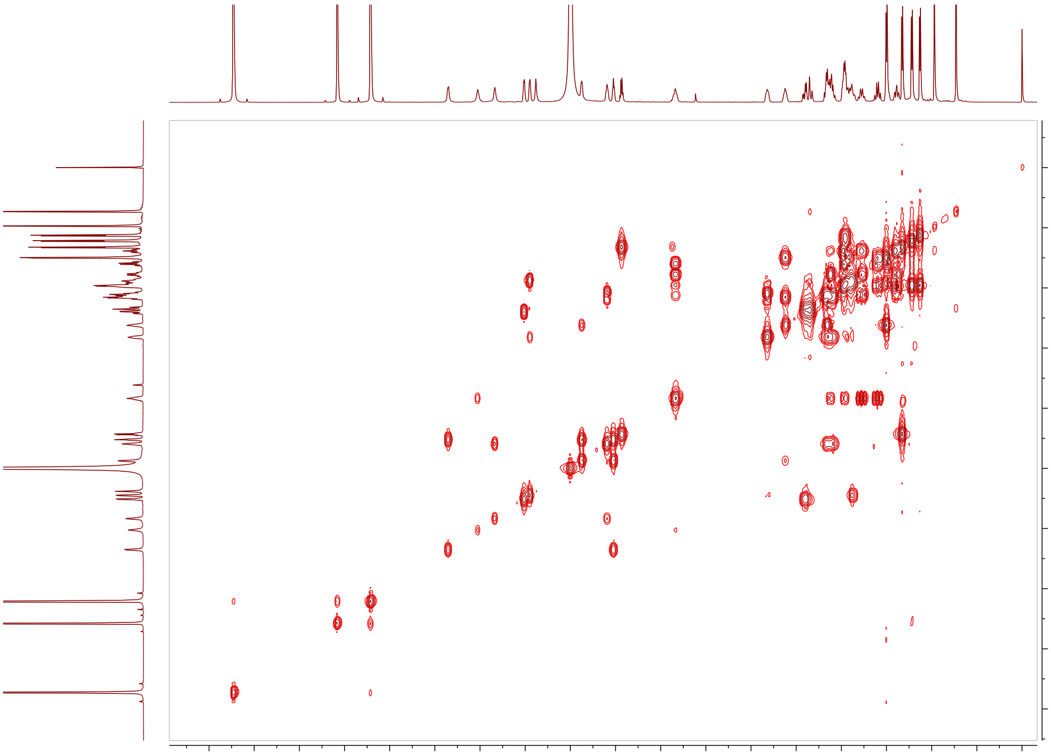

10.2.5 HSQC spectrum of 8 in pyridine-d_5

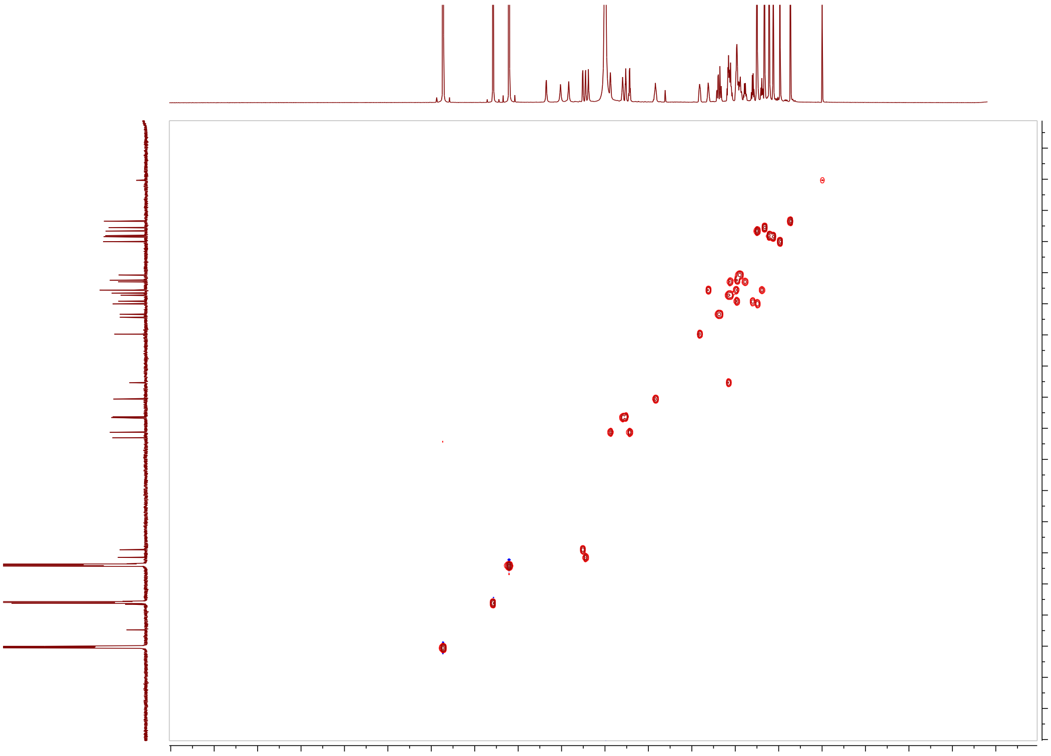

10.2.6 HMBC spectrum of 8 in pyridine-d_5

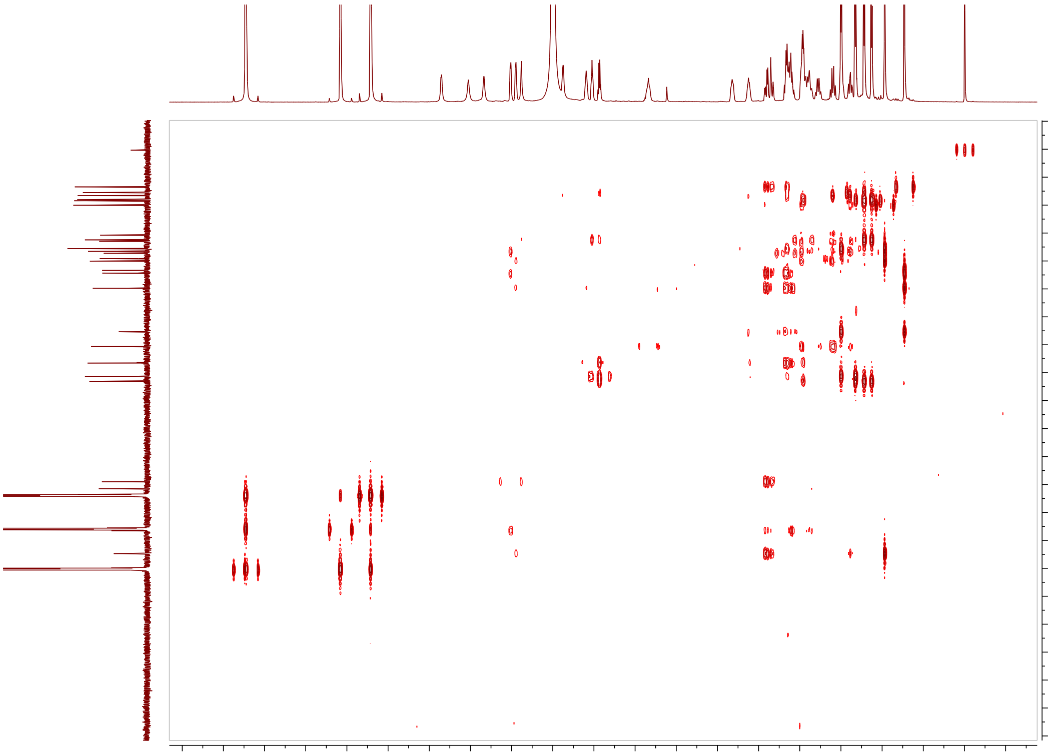

10.2.7 NOESY spectrum of 8 in pyridine-d_5

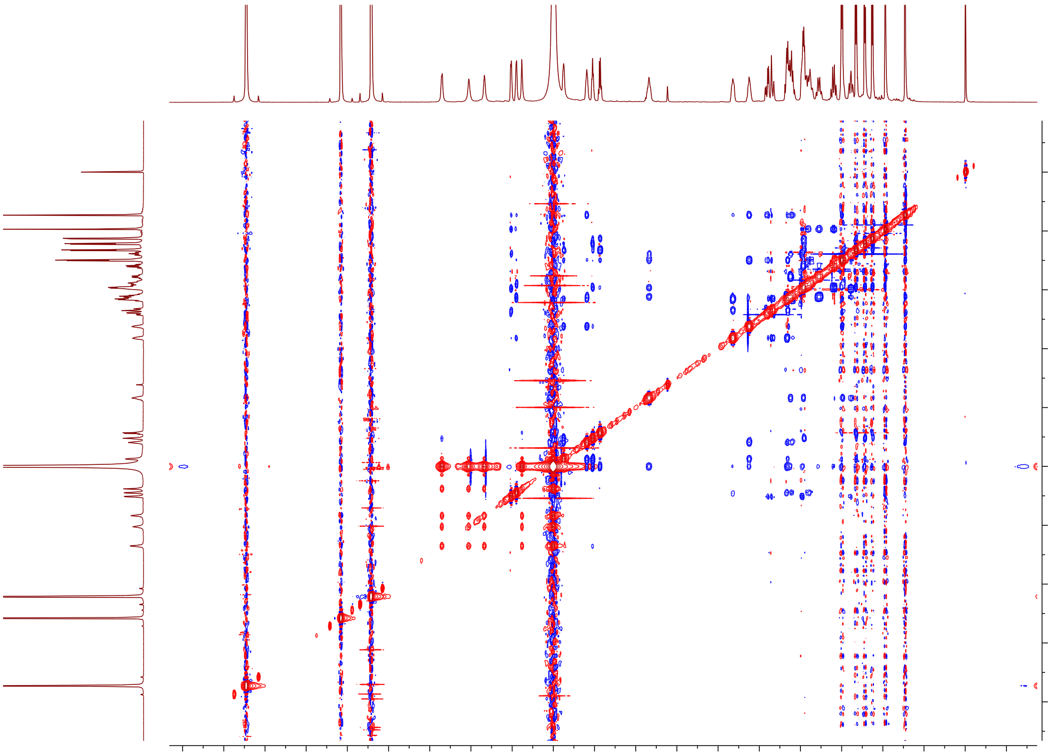

Supplement: Supplementary file 1 [file pharmaceuticals-15-01160-s001.zip › pharmaceuticals-1894175-supplementary.pdf]
